# Deprotonation suppressing via competitive proton transfer control for efficient perovskite solar cells

**DOI:** 10.1038/s41467-026-73620-0

**Published:** 2026-05-25

**Authors:** Hang Dong, Jinsong Qu, Songya Wang, Dazheng Chen, Wenming Chai, Weidong Wang, Weidong Zhu, He Xi, Long Zhou, Jincheng Zhang, Pengfei Huang, Yue Hao, Chunfu Zhang

**Affiliations:** 1 State Key Laboratory of Wide-Bandgap Semiconductor Devices and Integrated Technology, Faculty of Integrated Circuit, Xidian University, Xi’an, People’s Republic of China; 2 School of Mechano-Electronic Engineering, Xidian University, Xian, People’s Republic of China; 3 School of Advanced Materials and Nanotechnology, Xidian University, Xian, People’s Republic of China; 4 PetroChina Shenzhen New Energy Research Institute Co., Ltd., Shenzhen, People’s Republic of China

**Keywords:** Devices for energy harvesting, Solar cells

## Abstract

Despite the efficacy of the methylammonium chloride (MACl) additive strategy in stabilizing the α-phase of formamidine-based perovskite materials, a persistent and formidable challenge from the irreversible deprotonation of MA^+^ cation still bothers the fabrication of satisfactory formamidine (FA)-based perovskite photosensitive layers, thereby hindering the performance improvement of resultant perovskite solar cells. To confront this obstacle, numerous methodologies have been proposed and demonstrated their potential, particularly those leveraging additives enriched with carboxylic acid functional groups (-COOH). However, these approaches remain inherently flawed due to the deleterious competitive proton transfer from hydrogen iodide (HI) to the excess -COO^-^ species, which undermines the integrity of the entire strategy. Herein, an innovative methyl trifluoroacetate (MTFA)-assisted technique is pioneer conducted to control the competitive proton-transfer through its stepwise hydrolysis reaction and the gentle release of -COOH derived from trifluoroacetic acid (TFA) byproduct. By this way, the perovskite precursor solution deprotonation is suppressed and the shelf life of corresponding perovskite precursor solution is greatly prolonged to over 22 days. Concurrently, the robust affinity of -COOCH_3_ or byproduct -COOH groups toward uncoordinated Pb^2+^ has also been proven to enable the fine-tuning of perovskite crystallization dynamic, while the residual CF_3_COO^-^ species distributed in the buried interface of perovskite are considered to offer an additional improvement in film carrier behavior via the halogen vacancies passivation. Consequently, high-quality perovskite films with conspicuous crystalline structure, surface morphology and carrier characteristic were obtained, achieving a champion device fabricated from 22 days aging perovskite precursor solution (PPS) with a power conversion efficiency of 26.35% and retaining 94.75% of its initial efficiency after 1464 h of humidity exposure.

## Abstract (web summary)

Formamidine-based perovskite solar cells promise high efficiency, but additive-induced deprotonation disrupts precursor stability and device performance. Dong et al. use a hydrolyzing ester additive to regulate proton transfer, stabilizing the precursor and achieve efficient, durable devices.

## Introduction

Formamidinium lead iodide (FAPbI_3_) have gradually emerged as a prominent candidate among various active materials for high-efficiency perovskite solar cells (PSCs) owing to their unparalleled advantages, particularly their ideal bandgap of 1.48 eV, propelling the certified power conversion efficiency (PCE) of PSCs to exceed 27% within just over a decade^1–4^. This remarkable progress has underscored their significant industrialization potential and demonstrated promising substitution possibilities for their well-established silicon solar cell counterpart. It is well-known that FAPbI_3_ is primarily exists in two phases, a non-absorptive hexagonal phase (δ-phase) and a photoactive cubic phase (α-phase)^5–7^, yet the fabrication of photoactive α-FAPbI_3_ still poses substantial challenges due to its spontaneous phase transition from the black α-phase to the yellow δ-phase, which originates from the thermodynamic instability of α-FAPbI_3_ caused by the overlarge ionic radius of FA^+^ cations^8–10^. Fortunately, partial ion substitution strategies (replacing FA and/or I with Cs, methylammonium (MA) cation and Cl anion) have consistently been proven to optimize the tolerance factor value of FA-based perovskite. Consequently, they are expected to enable the preparation of stable α-phase FAPbI_3_^11–13^. Notably, the incorporation of methylammonium chloride (MACl) into the perovskite precursor solution is aimed at reducing the formation energy of the perovskite structure, promoting the preferential growth of visible-light absorbing α-phase FAPbI_3_ at room temperature, and thereby ensuring the fabrication of high-quality FA-based perovskite^14–18^. However, two critical shortcomings associated with the MACl incorporation strategy restrict the performance enhancement of resultant PSCs: i) Compared to other non-volatile chloride containing additives, the majority of MACl generally evaporates from the films during the initial stage of annealing process, this rapid sublimation is likely to induce the formation of undesirable halogen vacancy defects within resultant perovskite films due to the asynchronous volatilization of methylamine and chloride, leading to unacceptable non-radiative recombination loss^19,20^. ii) The FAPbI_3_ perovskite precursor solution (PPS) containing the MACl dopant is demonstrated to undergo inevitable irreversible degradation through MA^+^ deprotonation and associated side reactions, especially after prolonged storage^21–23^. Recent literature has established that reversible acid-based decomposition will trigger the deprotonation of MA^+^, resulting in the release of neutral methylamine (MA^0^) and volatile hydrogen iodide (HI)^24–27^. Notably, the liberated MA^0^ can readily undergo an amine-cation side reaction with FAI, leading to the formation of problematic MA-FA adducts such as non-volatile byproducts including N-methyl formamidinium (MFA^+^) and *N,N’*-dimethyl formamidinium (DMFA^+^)^22,28^. This detrimental side reaction is expected to cause a relative depletion of FA^+^ and potentially lead to a disruptive stoichiometric ratio in the PPS. Furthermore, the aforementioned MFAI byproducts are specified to combine with PbI_2_ to form trigonal MFAPbI_3_, which preferentially associates with δ-FAPbI_3_ to create a solid solution while being incompatible with α-FAPbI_3_, thereby impairing both phase purity and device performance^29–31^. In conjunction with the above analysis, the quality of MACl assisted FA-based perovskite films can be further amplified upon suppressing deprotonation, ascribed to its substantial influence on deteriorating the phase purity and reproducibility of α-FAPbI_3_.

Partial progressive efforts have therefore been implemented toward deprotonation inhibition as well as extending the shelf life of PPS, including precursor solvent allocation, low-temperature/anhydrous/inert environment storage, interface optimization technology and additive assistance approaches^21,32–36^. Therein, screening appropriate additives into the precursor solvent has been historically reported as a realizable strategy and indicates promising potential for inhibiting the synthesis of DMAI and stabilizing the precursor solution due to their well-leveraged properties and compatibility. Upon inspecting holotype additives, those containing acidic additives or functional groups have been widely recognized as effective corrosion inhibitors that suppress deprotonation in PPS, such as peroxyacetic acid, 4-hydrazinobenzenesulfonic acid (4-HBSA), 3-mercaptobenzoic acid (3-MBA), 1-(4-sulfophenyl)−3-methyl-5-pyrazolone (SMP)^37^, 4-(trifluoromethyl) phenylhydrazine (TFPH)^38^ and acidic PPS environment elicited by benzene-1,3-dithiol (BDT)^34,39–42^. Notably, additives with carboxyl acid functional groups (-COOH) exhibit exceptional efficacy in mitigating deprotonation and side reactions within PPS by forming robust two-point hydrogen bond with cations^43^.

Notwithstanding the aforementioned advancement, -COOH containing additives remain incapable of addressing the passivation of halogen vacancy defects derived from the asynchronous volatilization of methylamine and chloride during the annealing stage. Synergistic combinations of -COOH with other functional groups have demonstrated significant promise in several reports. Especially, trifluoroacetic acid (TFA), featuring a hydrophobic CF_3_COO^-^ species, can effectively passivate these vacancy defects by both passivating undercoordinated Pb^2+^ and suppressing moisture-induced iodide loss^44–48^. Nevertheless, it has been reported that the interaction between protons and TFA^-^ is stronger compared to that with I^-^, indicating that the superfluous TFA with an acid dissociation constants (p*K*_*a*_) of ~0.5 may increase the potential risk of competitive proton-transfer from HI (with a p*K*_*a*_ of −10.7) to TFA^-^, thereby accelerating MAI deprotonation by scavenging protons within HI^49,50^. Therefore, the development of a strategy to address proton-transfer becomes essential.

Herein, capitalizing on its gradual hydrolysis into TFA and volatile CH_3_OH, we employed methyl trifluoroacetate (MTFA) as a TFA alternative. This controlled decomposition enables not only moderated TFA release for sustained deprotonation inhibition, but also accelerated moisture depletion through methanol volatilization, thereby collectively suppressing cation deprotonation and prolonging PPS shelf life^51,52^. Additionally, the MTFA is also assigned to modulate the crystallization dynamic of relevant perovskite films through the formation of robust binding affinity between -COOCH_3_ (or its accompanying -COOH groups) and uncoordinated Pb^2+^ ions. This optimized PPS enables the fabrication of high-quality perovskite films exhibiting superior crystallinity, exceptional surface morphology and optimized carrier dynamic. Consequently, highly efficient and stable PSCs were successfully assembled, achieving a champion PCE of 26.35% and maintaining 94.75% of its initial efficiency after prolonged humidity erosion. This pioneering endeavor has unequivocally demonstrated the immense potential of anti-aging PPS for the fabrication of superior PSCs, paving the way for its future commercialization.

## Results

### Evolution of perovskite precursor solutions deprotonation

**Fig. 1:**
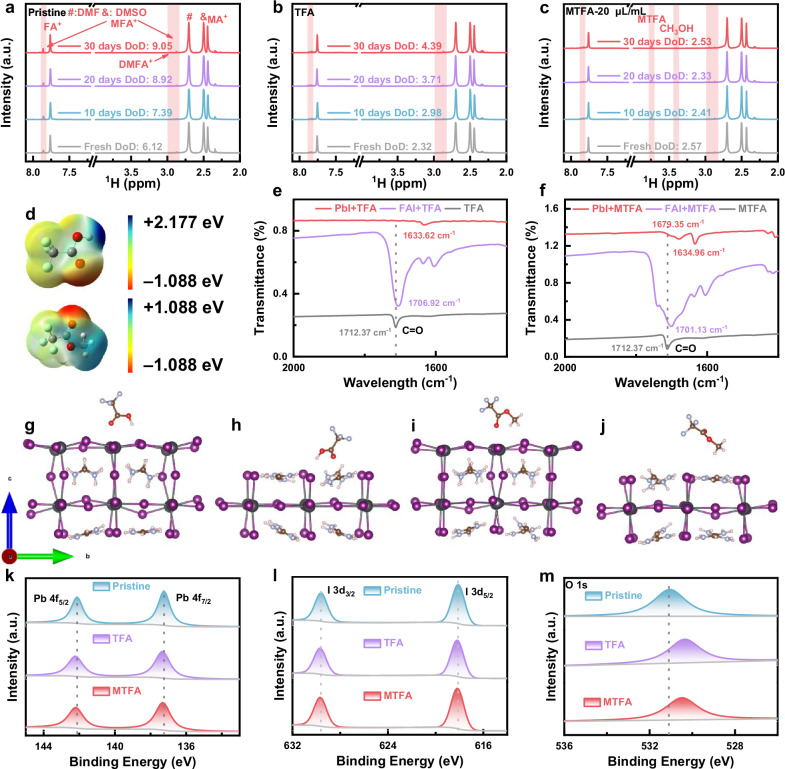
Chemical interactions and deprotonation suppression mechanisms of TFA and MTFA in perovskite precursor solutions. ^1^H NMR spectra of (**a**) pristine, (**b**) TFA and (**c**) 20 μL/mL MTFA PPSs at different aging periods, with all spectra normalized to the intensity of the DMSO-d_6_ signal as an internal reference. **d** Electrostatic potential distribution (ESP) of the TFA (above) and MTFA (below). FTIR spectra of (**e**) pure TFA, TFA + FAI and TFA+PbI_2_ powders; (**f**) pure MTFA, MTFA + FAI and MTFA+PbI_2_ powders. Cell structure of TFA interacted with (**g**) Pb-I framework and (**h**) FA as well as MTFA interacted with (**i**) Pb-I framework and (**j**) FA. XPS spectra of (**k**) Pb 4 f, (**l**) I 3 d and (**m**) O1s signals for perovskite films with different corrosion inhibitors.

The evolution of chemical species in MTFA-assisted PPSs was initially elucidated in this study through 30 days of continuous ^1^H nuclear magnetic resonance (NMR) spectroscopy detecting, with data extraction interval of 10 days and all samples were stored in the atmospheric environment ( ~ 25 °C and ~35% RH). As illustrated in Fig. 1a–c and Supplementary Fig. 1–7, comparative analyses were conducted on the fresh and aged PPS samples with and without TFA/MTFA, where TFA as a comparison additive is proposed to govern a more precise exploration and the efficacy of MTFA in suppressing deprotonation were systematically elucidated across discrepant doping concentrations (10/20/30 μL/mL). In Fig. 1a, Supplementary Fig. 1 and Supplementary Fig. 7a, two dominant signals appeared at ≈2.87 ppm and ≈7.86 ppm for the fresh pristine PSS sample are related to the MFA^+^ species, indicating that the MA^+^ in pristine PPS is deprotonated to form MA^0^ via chemical formula (1), followed by a concomitant amine-cation reaction^53,54^. Subsequently, as the aging time extended, ^1^H signals associated with the MFA^+^ species become more pronounced and the characteristic peak at ≈2.95 ppm were also visualized gradually, confirming the ongoing MA^+^ deprotonation process and the formation of DMFA^+^ species via detrimental side reactions trigger by chemical formula (2)^40,55^.

1 $${MAI}\rightleftarrows {{MA}}^{0}+{HI}$$

2 $${{MA}}^{0}+{{FA}}^{+}\to {{MFA}}^{+}+{{MA}}^{0}\to {{DMFA}}^{+}$$

Notably, signal intensities of both MFA^+^ and DMFA^+^ were climbed continuously with the passage of aging period, demonstrating sustained and uncontrolled deprotonation behavior within the pristine PPS. Concurrently, given that the content of H-containing molecules is correlated directly with their ^1^H signals area, the degree of deprotonation (DoD) in different PPSs was quantitatively determined by the precise calculation of byproducts molecular percentage on the basis of an area ratio equation: DoD = (A_MF_ + A_DM_)/(A_MF_ + A_DM_ + A_MA_ + A_FA_) in Supplementary Table 1^41^. It is apparent that DoD values of the pristine PPS have experienced a significant increase from 6.12% to 9.05% over the entire aging cycle, thereby disrupting the elemental stoichiometric ratios of corresponding PPS accompanied with exacerbating crystalline quality of relevant perovskite films^55,56^. Additionally, corresponding growth rate of the DoD was extracted in Supplementary Table 2.

From this, TFA and MTFA as corrosion inhibitors assembled with different groups (-COOH and -COOCH_3_) were incorporated for stabilizing PPSs. In the visual representation from Fig. 1b, c, Supplementary Fig. 2–7 and Supplementary Table 1, all corrosion inhibitors have eventually stabilized PPSs to varying extents during the initial stage, especially for the TFA-doped PPS (20 μL/mL). Notably, synthetic analysis revealed that the deprotonation processes of MA^+^ and FA^+^ have been markedly suppressed in the freshly prepared TFA-doped PPS sample, which acquired a profoundly reduced DoD value of merely 2.32%. In contrast, DoDs of all freshly prepared MTFA-doped PPSs (2.79%, 2.57% and 2.30% for 10/20/30 μL/mL respectively) were slightly higher than that of the TFA-doped one and declining gradually with enhanced MTFA doping concentration, implying a more pronounced efficacy of TFA during the initial stage in inhibiting the deprotonation of PPS compared to MTFA. However, as the aging time extended, similar to that of the pristine PPS, DoD of TFA-doped PPS underwent a continuous ascent with a dramatically growth rate from 2.32% to 4.39% over the whole aging period. Whereas, deprotonation evolutionary trend of all MTFA-doped PPSs was distinct from that of both pristine and TFA-doped PPSs, demonstrating a marked DoD values decline during the aging period from 0th to 20th days followed by an upturn, obtaining the minimum DoD values on the 20th days before rebounded. Moreover, DoD values of the MTFA-doped PPSs were inversely proportional to the concentration of MTFA at each sampling point, indicating a mirror ascending relationship between deprotonation efficacy and MTFA concentration.

This strikingly distinct deprotonation inhibition processes observed for corrosion inhibitors of TFA and MTFA is derived from their fundamentally different mechanisms of action in deprotonation suppressing. Therein, the robust deprotonation inhibition capability exhibited by the freshly prepared TFA-doped PPS is attributed to the acidic environment provided by -COOH of TFA as depicted in Supplementary Fig. 8a and chemical formula (3)^53^. However, isolated and magnified HI signal of the pristine/TFA/MTFA-doped PPS in Supplementary Fig. 9 demonstrated that the byproduct of volatility HI is only detected during the initial stage of the TFA-doped PPS, indicating a violently deprotonation reaction and implying the occurrence of competitive proton-transfer from HI to superfluous TFA^-^ induced by the stronger binding energy between TFA^-^ and H^+^, as illustrated in chemical formula (4), thereby deteriorating the composition of TFA-doped PPS after prolonged storage^36,57^. Which is further confirmed by the higher DoD growth rate in Supplementary Table 2 of the TFA-doped PPS compared to the pristine one. Ultimately, the superior deprotonation inhibition state is acquired by the MTFA-doped PPS sample as aging time extended capitalizing on its gradually released corrosion inhibitor of TFA via hydrolysis reaction in chemical formula (5). Therein, the trace amounts of water essential for MTFA hydrolysis are predominantly introduced through the highly hygroscopic nature of polar aprotic solvents (Dimethyl Sulfoxide (DMSO) and N,N-dimethylformamide (DMF)) as depicted in Supplementary Table 3, both the DMF and DMSO declared inelegant hygroscopicity after 10 days atmospheric storage ( ~ 25 °C and ~35% RH).

3 $$C{F}_{3}{COOH}\,\rightleftharpoons \,C{F}_{3}{{COO}}^{-}\,+{H}^{+}$$

4 $$C{F}_{3}{{COO}}^{-}+{HI}\to C{F}_{3}{COOH}\,+{I}^{-}$$

5 $$C{F}_{3}{COOC}{H}_{3}+{H}_{2}O\to C{F}_{3}{COOH}+C{H}_{3}{OH}$$

This could facilitate the suppressed deprotonation of PSS while simultaneously preventing the undesirable competitive proton-transfer induced by superfluous TFA^-^, as evidenced by the progressively increasing $C{H}_{3}{OH}$ signal over aging duration in Supplementary Fig. 10, the correspondingly mirrored decreasing MTFA signals in Supplementary Fig. 11, and the reducing PH values in Supplementary Fig. 8b. Notably, an upward reversal in DoD values for all MTFA-doped PPS samples were occurred between the 20th and 30th days derived from the approaching depletion of MTFA, as confirmed by the partial enlarged ^1^H NMR spectra in Supplementary Fig. 11. Overall, these finding determined that despite the TFA initially achieved a conspicuous deprotonation inhibition, the competitive proton-transfer from HI to superfluous TFA^-^ remains a critical factor in prolonging the duration and violence of deprotonation process, thereby resulting in a relatively higher DoD in relevant aging PPS. Whereas, replacing TFA with MTFA has effectively mitigated this unacceptable prolongation of deprotonation by enabling the gradual released TFA, which avoiding the unpopular competitive proton-transfer induced by superfluous TFA^-^ as well as extending the deprotonation suppression period of MTFA-doped PPS to over 20 days.

Furthermore, X-ray diffraction (XRD) characterization of perovskite films prepared with fresh or aged PPSs was also performed under the same sampling schedule to inquire into the influence of PPSs deprotonation degree on film crystalline characteristics, as well as confirming the accuracy of our above analysis. As illustrated in Supplementary Fig. 12, 13, the dominant diffraction signal observed at approximately 14.06° across all patterns is widely recognized as the (100) facet of the α-phase perovskite, serving as a key indicator for assessing the crystallinity of perovskite films. In contrast, the diffraction peak emerging at 12.70° is generally assigned to the unreacted PbI_2_ within the perovskite films. Notably, both the pristine and TFA samples exhibited a steady decline in the (100) diffraction intensity, accompanied by a gradual yet unmistakable increase in the PbI_2_ signal throughout the entire aging period, consistent with their time-dependent increased deprotonation degree as well as stepwise depletion of organic cation as revealed by our NMR analysis. However, all MTFA-doped samples established a steady ascend in the (100) peak intensity over the initial 20 days, along with a mirror decline in the PbI_2_ signal, followed by a reversal thereafter, according with their deprotonation degree evolutionary trend. Especially, upon comprehensive screening of all XRD patterns, the perovskite film fabricated from MTFA-doped PPS that underwent 20 days of aging has ultimately attained optimal crystalline, exhibiting the highest (100) peak intensity and a barely perceptible PbI_2_ residue derived from the effective suppression of deprotonation and exquisite modulation of crystallization dynamics, which collectively contribute to superior device performance.

Subsequently, the evolution of PCE extracted from the current density-voltage (*J-V*) curves of PSCs assembled with aforementioned perovskite films were plotted in Supplementary Fig. 14. This analysis validated both the aging time-dependent deprotonation extent of PPSs incorporating different corrosion inhibitors and crystalline of relevant perovskite films, corroborating the findings without any contradiction as well as precisely pinpointed the peak performance of MTFA-doped PSCs was achieved on the 20th, 22nd, and 24th days as the MTFA doping concentration of 10/20/30 μL/mL. Furthermore, *J-V* curves of PSCs fabricated with freshly prepared and 22 days aging PPSs were displayed in Supplementary Fig. 15, 16, with corresponding photoelectric performance parameters enumerated in Supplementary Table 4-5, indicating that the champion PCE for both the fresh and aged PSCs were achieved by the 20 μL/mL MTFA-doped device with values 26.24% and 26.35% respectively.

Therefore, chemical species evolution tests were further conducted on a bromide-rich wide-bandgap (WB) PPS with the composition of Cs_0.15_FA_0.65_MA_0.20_Pb(I_0.80_Br_0.20_)_3_ to further verify the universality of the MTFA (20 μL/mL) doping strategy in PPS deprotonation suppressing, the bandgap of this perovskite materials is approximately 1.68 eV^58^. As depicted in Supplementary Fig. 17-19 and Supplementary Table 6, DoD values of both the pristine and MTFA-doped WB PPS were strictly adhered to an evolutionary history analogous to that of the normal-bandgap PPS. Concretely, DoD values of the pristine WB PPS exhibited a monotonic gradual increase across the entire aging period while those of the MTFA-doped WB PPS decreased initially and then subsequently increased with day 20th as the dividing line. The mirror ascending and descending signals of MTFA and CH_3_OH in Supplementary Fig. 20 have further confirming the hydrolysis reaction of MTFA within PPS and affirming the superiority of MTFA-doping strategy in deprotonation suppression via controlled TFA release.

Briefly, the foregoing analysis declared that both corrosion inhibitors demonstrated remarkable and universal efficacy in suppressing the deprotonation of PPS, especially in the MTFA-doped PPSs and the optimal PSCs performance was acquired with the 20 μL/mL MTFA-doped PPS on the 22th days. Accordingly, the subsequent research focus will be limited to the pristine, TFA, and 20 μL/mL MTFA-doped samples to systematically distinguish the effects of the two corrosion inhibitors on perovskite film quality and PSC performance. Subsequently, for the sake of clarity and consistency, the 20 μL/mL MTFA-doped sample/device will henceforth be referred to as the MTFA sample/device to improve the readability of subsequent discussion.

### Molecule interaction mechanisms

An extraordinary and striking observation that demanded emphatic attention in aforementioned analysis is the apparent conflict between the superior deprotonation suppression capability of the 30 μL/mL MTFA-doped PPS and the exceptional crystallinity as well as higher photovoltaic performance exhibited by the 20 μL/mL MTFA-doped precursor or corresponding device. Which is probably derived from the more standardized crystallization dynamics belonged to the moderate MTFA-doping concentration. Consequently, the coordination affinity between two corrosion inhibitors and perovskite films was firstly investigated comprehensive. Molecular dynamic calculation was initially implemented to shedding light on the intricate charge distributions of TFA, MTFA and HI through electrostatic surface potential (ESP) maps in Fig. 1d and Supplementary Fig. 21. The electron depletion region of TFA is prominently centered around the hydroxyl (-OH) hydrogen accompanied by a distinctly positive potential of approximately + 2.177 eV, whereas the carbonyl (C = O) oxygen and hydroxyl oxygen within the carboxyl group display pronounced electronegativity, underscoring their strong coordination affinity with Pb^2+^ in Pb-I framework or FA^+^ as well as electron-accepter defects capability. In contrast, the electron depletion region of MTFA is evenly distributed around the methoxy group (-OCH_3_), while the electron cloud is aggregated at the carbonyl oxygen along with a slightly lower negative potential of −1.088 eV. Notably, the ESP map of HI in Supplementary Fig. 21 displayed an evidently polarized distribution pattern, where the hydrogen atom manifested a typically electropositivity characteristics and the electronegativity region was predominantly localized around the iodine atom. The conspicuously greater potential difference exhibited by TFA in comparison to HI maps to H atom binding energies of −20.29 eV for CF_3_COO^-^ and −4.75 eV for I^-^, offering indirect yet compelling evidence for the occurrence of competitive proton-transfer from HI to excess TFA^-^. Furthermore, the adsorption energies (*E*_*ads*_) between TFA/MTFA and either the Pb-I framework or FA^+^ were systematically computed through density functional theory (DFT) as depicted in Fig. 1g–j and Supplementary Fig. 22. The calculated values indicated a progressive enhancement in *E*_*ads*_ with respect to the Pb-I framework from −4.31 eV for MTFA to −4.67 eV for TFA. Since these values were decreased rapidly when associated with FA^+^ cation, yielding *E*_*ads*_ of −1.72 eV and −2.21 eV for TFA and MTFA respectively. Declaring that all corrosion inhibitors are likely to coordination with Pb-I framework rather than with FA^+^, particularly in the case of TFA.

Subsequently, Fourier transform infrared (FTIR) spectroscopy illustrated in Fig. 1e, f was further employed to substantiate the aforementioned simulation results as well as clarify the molecular interaction between the TFA/MTFA and perovskites materials. To this end, each corrosion inhibitor was separately combined with either the PbI_2_ or FAI powders, and the characteristic peak in range of 1720–1630 cm^−1^ were related to the C = O stretching vibrations^59^. Remarkably, all observed characteristic peak exhibited a distinct red shift toward lower wavelengths, indicting the formation of coordination bands between the C = O with uncoordinated Pb^2+^ or FA^+^^60^. As depicted in Fig. 1e, upon mixing TFA with PbI_2_, the C = O signal was shifted from 1712.37 cm^−1^ to 1633.62 cm^−1^, while the C = O signal of TFA + FAI mixed powders was spotted at 1706.92 cm^−1^, with displacements of 78.75 and 5.45 cm^−1^. Turning in Fig. 1f, MTFA displayed a typical C = O signal at 1712.37 cm^−1^ and moved to 1701.13 cm^−1^ upon the addition of FAI (shift of 11.24 cm^−1^). Notably, two distinct vibrational peaks (1679.35 and 1634.96 cm^−1^) emerged prominently in the MTFA + PbI_2_ spectrum, which were assigned with C = O → Pb^2+^ coordination modes originating from MTFA and TFA respectively. This provides direct evidence for the coexistence of both MTFA and its hydrolysis product TFA, as well as their simultaneous coordination to the Pb-I framework, laterally confirming the occurrence of its unique hydrolysis reaction. Overall, this phenomenon suggests that the coordination interactions between TFA/MTFA and Pb^2+^ were significantly stronger than those with FA^+^, following the order of TFA + PbI_2_ > MTFA + PbI_2_ > MTFA + FAI > TFA + FAI, which was in excellent agreement with the DFT simulation results.

The intricate chemical interplay between corrosion inhibitors and perovskite was further inquired through X-ray photoelectron spectroscopy (XPS) analysis. As depicted in Fig. 1k–l and Supplementary Fig. 23, two dominant diffraction peaks positioned at 142.20 and 137.33 eV were associated with the Pb 4f_5/2_ and Pb 4f_7/2_ signals of pristine films respectively. Upon the incorporation of TFA and MTFA, both peaks underwent a distinct blue shifted toward higher binding energy, indicating a notable depletion in the electron cloud surrounding the Pb^2+^ derived from the coordination interactions between TFA/MTFA and Pb^2+^ as well as the inherently greater electronegativity of O atom relative to the Pb atom. Notably, the substantially enlarged shifting extent of TFA sample comparted to the MTFA one implies the heightened chemical affinity of TFA to inorganic slabs for enhanced interaction with Pb^2+^. This inference is further substantiated by the analogous shift pattern discerned in the I 3 d spectrum. In contract, diffraction peaks of the O 1 s in Fig. 1m were red shifted toward lower binding energy upon the mixing of TFA and MTFA, which is chemically correlated with the opposing shifts observed for the Pb 4 f and I 3 d core levels. Especially, combined with analysis from DFT simulations and FTIR spectroscopy, this red-ward shift is specifically attributed to the C = O group of corrosion inhibitors upon coordinating with the Pb-I lattice, rather than potential ambient adsorbates or impurities in perovskite films. This provided compelling evidence for the formation of C = O → Pb^2+^ coordination motifs between the inhibitors and Pb-I lattice, underscoring the superior chemical engagement exhibited by the TFA-based corrosion inhibitor.

Additionally, X-ray absorption spectroscopy (XAS) was further conducted to inquire the atomic environment around Pb^2+^ and to confirm the molecular interaction mechanisms elucidated by FTIR, XPS, and DFT calculations. Therein, X-ray absorption near-edge structure (XANES) spectra of perovskite films with various corrosion inhibitors were illustrated in Supplementary Fig. 24a, b, revealing a clear blue shifted Pb L_3_-edge absorption edge for both the TFA and MTFA samples compared to the pristine one, with the most pronounced blueshift observed for the TFA sample. This blue shift indicated a reduction in electron density around Pb^2+^, arising from coordination interactions between TFA/MTFA and Pb^2+^ sites and further amplified by the higher electronegativity of oxygen. Notably, the local bonding environment was further acquired using Fourier transform extended X-ray-absorption fine structure (FT-EXAFS) spectra in Supplementary Fig. 24c, d. Two dominant signals appeared at ≈1.91 Å and ≈2.97 Å in R-space for all samples are related to the Pb-O band and Pb-I band respectively. All corrosion inhibitors samples assumed a markedly attenuated Pb-I peak intensity accompanied by an enhanced Pb-O signal, especially for the TFA sample. Undoubtedly, both the simulating calculation and experimental analyses collectively revealed that all inhibitors exhibit a pronounced capacity for deprotonation suppression, concomitant with the formation of C = O → Pb^2+^ coordination bonds according to the Pb-I lattice. Given that the nuclear formation process of perovskite materials predominantly initiates within the Pb-I lattice, while the rate of crystal growth is dictated by the efficiency with which organic cations integrate into the Pb–I octahedral framework during the thermal annealing phase. It is essential to inquiring the untapped potential of corrosion inhibitors in regulation the crystallization behavior of perovskite.

### Investigation on perovskite films crystallization behavior

**Fig. 2:**
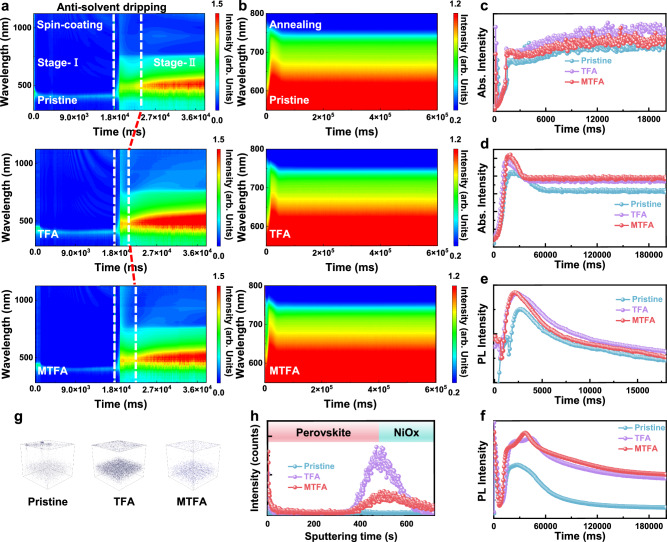
Crystallization dynamics of perovskite films treated with/without corrosion inhibitors. In situ UV-Vis images of the perovskite films during the (**a**) spin-coating and (**b**) annealing process. Evolution of absorption peak intensity at 480 nm for perovskite films during (**c**) spin-coating and (**d**) annealing process. Evolution of PL peak intensity at 760 nm for perovskite films during (**e**) spin-coating and (**f**) annealing process. (**g**) The 3D spatial distribution of CF_3_COO^-^ for the pristine/TFA/MTFA films. (**h**) ToF-SIMS depth-profile analysis of CF_3_COO^-^ in the pristine/TFA/MTFA films.

Moreover, given the pronounced sensitivity of perovskite crystallization dynamic to both the inhibited deprotonation within PPS and the stronger coordination interactions between inhibitors and the Pb-I lattice framework. In-situ ultraviolet-visible (UV-Vis) and in-situ photoluminescence (PL) measurements were also conducted to elucidate the nucleation and crystal growth evolution of perovskite films with various inhibitors throughout the spin-coating and annealing processes. It should be emphasized that all subsequent investigations are based on the aged PPS. As depicted in Fig. 2a, all samples suggested bifurcated phase evolution stages with the dividing line of the CB quenching operation. Thereinto, stage-Ⅰ consisted in the 0 ms to approximately 18000 ms is related to the initial PPS drop-coating and spinning step with the prominent absorption peak located at ~400 nm. Notably, a consistent blue shift in peak position was detected over time within this stage, which can be attributed to the rapidly solvent depletion induced by the centrifugal force, indicating the relatively analogous optical properties of perovskite films containing different corrosion inhibitors during this early stage. Subsequently, stage-Ⅱ was emerged rapidly upon the injection of chlorobenzene (CB), with an exponentially expanding cutoff edge to ~800 nm for all samples along with an enhancement in absorption intensity, indicating the solvent supersaturation triggered nuclear aggregation. However, it is essential to emphasize that all corrosion inhibitors injection samples have acquired a significantly enhancement in light absorption intensity compared to that of the pristine one, with the TFA sample demonstrating the most pronounced effect. Moreover, phase transition point of these samples was abided by a distinct sequential order of TFA, MTFA, and finally the pristine sample. All aforementioned analysis was further substantiated by curves displayed in Fig. 2c, where the absorbance evolutions at the peak wavelength of 500 nm was precisely extracted to clearly illustrate the optical properties and the associated nucleation behavior of the perovskite films during stage-Ⅱ. Additionally, in-situ PL spectrum illustrated in Supplementary Fig. 25a and Fig. 2e were also proposed to inquire the phase evolution of perovskite films, all samples declared a substantial enhancement in emission peak accompanied by the gradual red-shifted cutoff edge once the CB was injected onto the pre-cast films. The ranking of emission peak intensity and phase transition point is fully consistent with the results we extracted from the in-situ UV-Vis spectrums. These findings declare that the incorporation of corrosion inhibitors could certainly promoting the nuclear aggregation within the precast perovskite films, especially for the TFA-doped sample, which is likely ascribed to its stronger interaction with the Pb-I lattice.

Furthermore, in-situ UV-Vis and in-situ PL spectrograms during the annealing process were further proposed after transferring the precast films to a hotplate, with the aim of gaining insights into the crystal growth behaviors of perovskite films containing various corrosion inhibitors. As depicted in Fig. 2b, two sequential absorption phase evolutions were acquired across all samples throughout the whole monitoring period: an initial absorption enhancement stage and the subsequent plateau stage. Relevant absorbance evolutions extracted at the peak wavelength of 500 nm were illustrated in Fig. 2d, thereby comprehensively inquiring into the crystal growth behaviors of perovskite films during the annealing process. During the ascending stage, both the TFA and MTFA samples demonstrated the highest initial absorbance coupled with a skyrocket enhanced in absorbance compared to the pristine sample, with the TFA sample showing the most pronounced effects. This compelling observation aligns remarkably well with the nucleation density ranking presented in Fig. 2a, indicating a potential functionality of TFA/MTFA in modulating the crystal growth of perovskite films owe to the enhanced interaction between the TFA/MTFA and Pb-I lattice as well as the resulting drastic nucleation aggregation. Subsequently, all samples were transitioned into a plateau stage as the annealing duration extended, while the champion absorption intensity in this region is belonged to the MTFA sample, thereby underscoring its superior crystalline characteristic induced by the synergistic action of suitable crystallization dynamic regulation and balanced stoichiometric ratio within PPS derived from suppressing cation deprotonation. A similar tendency was also detected in the in-situ PL spectrograms as shown in Supplementary Fig. 25b and Fig. 2f, substantiating the credibility of the aforementioned analysis. Synthesizing these in-situ images, it is concluded that the crystallization behavior regulation of both the TFA and MTFA samples are not only function in the adjusting initial absorbance, but also the crystal growth rate of relevant perovskite films.

Additionally, we also investigated the spatial distribution of TFA and MTFA within the final perovskite films using the time-of-flight secondary-ion mass spectrometry (ToF-SIMS) in Fig. 2g, h and Supplementary Fig. 26, 27. The results unveiled that both the TFA and MTFA were preferentially enriched at the buried interface of the perovskite films and the residual concentration of TFA is approximately threefold higher than that of MTFA. This unique distribution pattern of the corrosion inhibitors can be primarily attributed to their robust binding affinity with the Pb-I lattice^61^, leading to impeded volatilization of them. Consequently, this favorable localization facilitates efficient passivation of deep-level defects and significantly enhances carrier transport properties^62^.

### Morphology, structural and optoelectronic properties of perovskite films

**Fig. 3:**
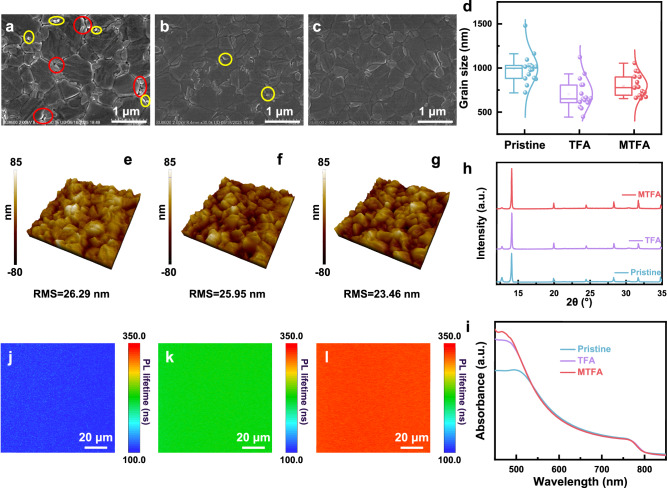
Characterization of perovskite films treated with/without corrosion inhibitors. Top view SEM images of (**a**) the pristine sample, (**b**) the TFA sample and (**c**) the MTFA sample. Scale bar, 1 μm. (**d**) Statistical grain sizes of final perovskite films. AFM images and corresponding RMS values of (**e**) the pristine sample, (**f**) the TFA sample and (**g**) the MTFA sample. (**h**) XRD patterns and (**i**) UV–vis absorption spectrum of final perovskite films. PL lifetime in PL mapping images of (**j**) the pristine sample, (**k**) the TFA sample and (**l**) the MTFA sample. Scale bar, 20 μm.

Synergistically optimized by the suppression of cation deprotonation and the precise regulation of crystallization dynamic, top-view scanning electron microscopy (SEM) and atomic force microscopy (AFM) measurements were ordinal inspected to evaluate the crystalline quality and surface morphology of resultant perovskite films. For the purpose of comprehensive comparative analysis, SEM images of perovskite films were prioritized enumerating as a key approach to reveal the surface morphology evolution induced by various corrosion inhibitors. As displayed in Fig. 3a–c, profiting from the enhanced nucleation density and precisely controlled crystal growth, all corrosion inhibitors containing samples demonstrated significantly more compact and smoother surface textures relative to the pristine one, albeit with a slight reduction in grain size in Fig. 3d (the average grain sizes were calculated to be 994.32 nm, 705.66 nm and 801.77 nm for the pristine/TFA/MTFA samples respectively). Notably, the densely arranged cracks, typically distributed along grain boundaries (as marked by red ovals), are absent in the corrosion inhibitor samples attributed to the enhanced nucleation density, signifying a considerable decrease in non-radiative recombination centers and a significant improvement in carrier dynamic^63–65^. Additionally, it is widely recognized that white flakes (highlighted by yellow ovals) adorning the surface of the pristine and TFA samples are classified as unreacted PbI_2_ species, originating from the disruptive stoichiometric ratio within the PPS induced by the uncrossed cation deprotonation^66,67^. The reliability of SEM images was further substantiated by AFM images presented in Fig. 3e–g, which enabled a precise quantitative comparison of the root-mean-squared (RMS) roughness exhibited by the perovskite films. Notably, significant reduced RMS values of 23.46 nm and 25.95 nm were assigned to the MTFA sample and TFA sample respectively, whereas the RMS value of the pristine sample was defined as 26.29 nm. This reduction in RMS values is positively correlated with the compact and dense surface morphology observed in the corrosion inhibitor samples through SEM imaging. Furthermore, the water contact angle extracted from the water-repellent of perovskite films in Supplementary Fig. 28 reveal a markedly enhance hydrophobicity in the corrosion inhibitor samples is significant enhanced compared to the pristine sample. Overall, the corrosion inhibitors assisted strategy has paved the way for fabricating perovskite films with eliminated grain boundary cracks, smooth surface texture and improved resistance moisture erosion capacity.

As shown in Fig. 3h, crystalline characteristics of perovskite films were meticulously analyzed through XRD analysis, revealing a predominant (100) facet of α-phase perovskite across all samples as evidenced by the presence of prominent diffraction signals at 14.08°^68^. The partially magnified (100) diffraction peak was thereby displayed in Supplementary Fig. 29 to enable a more refined and accurate comparison of crystalline variations. Which determined that all corrosion inhibitor samples have realized a robust (100) diffraction peak in contrast to the pristine counterpart ascribed to their superior crystalline quality, especially for the MTFA sample. Notably, it is essential to highlight that the sight peak protrusion observed at 12.75° in both the pristine and TFA pattern is assigned to the unreacted PbI_2_, which is almost absented in MTFA samples. This finding aligns remarkably well with the quantity of white flakes presented in SEM images, indicating a more complete reaction within corrosion inhibitor samples, especially for the MTFA film attributed to the suppression of cation deprotonation and the regulation of crystallization dynamics.

Additionally, the unignored diffraction peak red-shift of both the TFA and MTFA samples is likely induced by the releasing tensile strain which is further assessed through the grazing-incidence X-ray diffraction (GIXRD) technique with 2*θ*-sin^2^*ψ* method^69–72^. As illustrated in Supplementary Fig. 30, both the pristine and MTFA samples requested a prominent leftward scattering peak shift along the extended *ψ* from 10° to 50°, with the magnitude of the shift following the sequence: the pristine sample > the MTFA sample. In contrast, shift direction of the TFA sample was turned to the rightward under identical experimental condition. These distinct scattering peaks behaviors unveil the intricate variations in lattice spacing along the in-plane direction at enhancing detection depth, indicting the presence of diverse residual strain ($\sigma$) states within the perovskite films. Which were quantitative by the following equation according to Bragg’s Law and generalized Hooke’s Law:

6 $$\sigma=-\frac{E}{2\left(1+\nu \right)}\frac{\pi }{180}\cot {\theta }_{0}\frac{\partial \left(2\theta \right)}{\partial {\sin }^{2}\psi }$$

Where $E$ and $\nu$ assign to the Young’s modulus (ranging from 10–20 GPa) and Poisson’s ratio (approximately 0.3) of perovskite films respectively^73,74^, ${\theta }_{0}$ denotes the half of the scattering angle 2${\theta }_{0}$ associated with a specific diffraction peak position in the strain-free perovskite structure, $\theta$ (31.68°) corresponds to the diffraction peak of the perovskite film tested in this work. Notably, the section of $\partial \left(2\theta \right)/\partial {\sin }^{2}\psi$ is artificially extracted and defined as *k* to facilitate the assessment of strain magnitude and classification. Since the positive slopes (*k* > 0) of the fitted lines signify compressive strain, whereas negative slopes (*k* < 0) imply the presence of tensile strain in the perovskite films. While the *k* values of perovskite films obtained from the linear fitting curves of $2\theta$ versus ${\sin }^{2}\psi$ are calculated as −0.092, 0.037 and −0.016 for the pristine/TFA/MTFA samples as presented in Supplementary Fig. 31 and Supplementary Table 7. These results underscored the potential efficacy of corrosion inhibitors in alleviating undesirable residual strain, especially evident in the case of the MTFA sample, whose residual strain was drastically diminished to +7.71 MPa, compared to +44.30 MPa for the pristine sample and −17.82 MPa for the TFA sample. This favorable strain relaxation is likely triggered by synergistic effects, particularly suppressed protonation, which helps maintain the stoichiometric balance of PPS and reduce defect-induced lattice distortion, along with moderated crystallization dynamics that promote a more homogeneous and strain-balanced perovskite lattice^69,71,75,76^. Notably, the near-strain-relaxed state acquired within the MTFA sample warrants special attention owing to its progress in optimizing carrier dynamic, preserving an undistorted lattice structure and boosting intrinsic stability.

Moreover, UV-Vis spectra presented in Fig. 3i reveal that the MTFA sample have acquired a significantly enhanced absorbance in the short wavelength region compared to both the pristine and TFA samples, which is assigned to its optimized surface morphology and superior crystalline quality. Strikingly, according to the Tauc plot curves in Supplementary Fig. 32, narrow bandgaps were detected for the corrosion inhibitor samples, aligning well with the relaxed tensile strain within relevant perovskite films^77^. Subsequently, steady-state PL excited from the front side (perovskite film) and back side (glass), PL mapping images and time-resolved photoluminescence (TRPL) decay profiles were proposed successively to evaluate the carrier dynamic of the three samples. As illustrated in Supplementary Fig. 3a, b, the incorporation of corrosion inhibitors directly caused dramatically enhanced emission peaks in the PL spectra acquired from both the front-side and back-side of relevant samples compared to the pristine one, declaring their optimized crystalline characteristic and reduced carrier recombination centers. Notably, a more pronounced peak intensity gap between the MTFA and TFA samples was emphasized in the front-side excited PL spectra, indicating superior suppression of non-radiative recombination at the buried interface of the TFA sample. This improvement is likely attributed to the effective passivation of deep-level defects at its buried interface triggered by the aggregated TFA, as shown in Fig. 2g. Additionally, microscopically red-shifted emission peaks (especially for the TFA sample) were also observed for corrosion inhibitor samples. This phenomenon is mainly ascribed to their effective compensation of tensile strain as well as the elimination of non-radiation carrier recombination centers. Sequentially, the optimized defects inhibition mechanism was further substantiated by the PL mapping images presented in Fig. 3j–l, suggesting that the PL lifetime extracted from the TFA sample is extended than that of the pristine sample, and is further enhanced in the MTFA sample. Additionally, the PL intensity histogram (Supplementary Fig. 34), derived from analyzing randomly selected data points along a vertical axis within the PL mapping dataset, was proposed to elucidate the spatial distribution of emission intensity across the sample. Notably, consistent and superior surface charge characteristics of the MTFA sample was evidenced by the elevated median PL intensity (328.02) and the narrower full width at half maximum (14.07). TRPL decay profiles illustrated in Supplementary Fig. 3c was fitted by a bi-exponential decay equation to elucidate the average photo-generation carrier lifetime (${\tau }_{{avg}}$) within perovskite films:

7 $${\tau }_{{avg}}=\frac{{A}_{1}{\tau }_{1}^{2}+{A}_{2}{\tau }_{2}^{2}}{{A}_{1}{\tau }_{1}+{A}_{2}{\tau }_{2}}$$

Herein, ${A}_{1}$ and ${A}_{2}$ represent the respective amplitudes, while ${\tau }_{1}$ and ${\tau }_{2}$ denote the PL decay times. As enumerated in Supplementary Table 8, the average lifetime indexed to the TFA and MTFA samples was extended to 259.88 ns and 301.90 ns respectively compared to 191.17 ns for the pristine sample. This indicates the capacity of the corrosion inhibitor samples in promoting charge extraction and transfer behavior, with the MTFA sample achieving the most pronounced improvement. Collectively, these results compellingly imply the extraordinary efficacy of MTFA in suppressing cation deprotonation, passivating halogen vacancy as well as orchestrating crystallization dynamic, which are indispensable for the fabrication of high-quality perovskite film with conspicuous crystalline characteristic and surface texture characteristic, accompanied by satisfactory carrier dynamic.

### Photovoltaic performance evaluation of PSCs

**Fig. 4:**
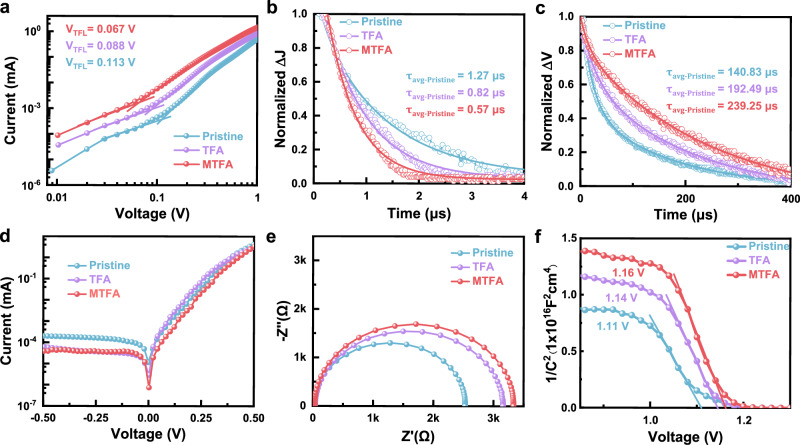
Carrier dynamics and defect characterization of perovskite solar cells. (**a**) SCLC analyses, (**b**) TPC curves, (**c**) TPV curves, (**d**) dark *J-V* curves, (**e**) Nyquist plots and (**f**) M-S plots of final PSCs.

Further investigation was governed by the conduction of a space-charge-limited current measurements based on a hole-only device with the architecture of ITO/NiO_X_/Perovskite/Spiro-OMeTAD/Ag (refer to Supplementary Fig. 35), which were performed under a dark condition and the defect densities (${n}_{t}$) within perovskite films were quantitatively evaluated by the formula of ${n}_{t}=2\varepsilon {\epsilon }_{0}{V}_{{TEL}}/e{L}^{2}$. Here, $\varepsilon$ and ${\epsilon }_{0}$ stand for the relative dielectric constant and the vacuum permittivity of the perovskite material, respectively. ${V}_{{TEL}}$ indicates the trap-filled voltage extracted from the SCLC curve, $e$ denotes the fundamental charge, while $L$ indexes to the thickness of relevant perovskite films. As displayed in Fig. 4a, ${V}_{{TEL}}$ values for corrosion inhibitor films (0.088 V for the TFA film and 0.067 V for the MTFA film) was notably inferior to that of the pristine film (0.113 V). The quantified value of ${n}_{t}$ were therefore reduced from 3.24 × 10^14 ^cm^−3^ for the pristine film to 2.53 × 10^14 ^cm^−3^ and 1.92 × 10^14 ^cm^−3^ for the TFA and MTFA films respectively. This underscores the remarkable efficacy of corrosion inhibitors in eliminating defects within perovskite films, rooted in their dual capacity to suppress cation deprotonation, passivate halogen vacancies and delicately modulate crystallization dynamics, thereby orchestrating a profound enhancement in film morphology, particularly in the case of the MTFA sample.

Additionally, transient photocurrent (TPC) and transient photovoltage (TPV) measurements were meticulously carried out to systematically elucidate the enhanced carrier dynamic conferred by corrosion inhibitors at the operational level of the device. Therein, the photocurrent decay process is correlated with the charge extraction and transport capability, while the photovoltage decay rule is generally used to inquire the charge recombination characteristics within PSCs. As depicted in Fig. 4b, c, a sharply shortened photocurrent lifetime (0.57 μs) is assigned to the MTFA device compared to both the TFA device (0.82 μs) and the pristine device (1.27 μs), indicting a notable enhancement in carrier extraction and transport for devices upon the incorporation of MTFA. Concurrently, the photovoltage decay behavior in the MTFA device is markedly slower than that observed in the TFA device and the pristine device, implying a prolonged carrier lifetime within the relevant PSC. This enhanced lifetime indexes to a reduction in trap densities, coupled with a substantial suppression of non-radiative recombination processes. Subsequently, guided by the fundamental principles of the diode law in solar cells, dark *J-V* curves of the aforementioned PSCs were further performed to evaluate their carrier recombination properties and defect states through the evaluation of dark saturation current density (*J*_*dark*_). As illustrated in Fig. 4d, the substantially reduced *J*_*dark*_ value of the MTFA device (7.31 × 10^−7 ^mA/cm^2^) aligns remarkably well with its retarded charge recombination behavior, whereas the *J*_*dark*_ values for the pristine and TFA devices are determined to be 1.06 × 10^−5 ^mA/cm^2^ and 2.77 × 10^−6 ^mA/cm^2^ respectively.

Additionally, electrical impedance spectroscopy (EIS) curves acquired under the dark conditions with an open-circuit potential of 1.0 V were also proposed in Fig. 4e to assess the carrier properties of PSCs. The corresponding equivalent circuit model of the complete solar cells were illustrated in Supplementary Fig. 36, while the fitted series resistance (*R*_*s*_) and recombination resistance (*R*_*rec*_) were enumerated in Supplementary Table 9. Apparently, the MTFA device has achieved a lower *R*_*s*_ of 30.85 Ω and a higher *R*_*rec*_ of 3304 Ω compared to the pristine device of 38.78 Ω and 2491 Ω, these results reconfirm the enhanced carrier extraction and transport capability as well as suppressed charge recombination of the MTFA device that we detected from TPC, TPV and dark *J-V*. Whereafter, as shown in Fig. 4f, the Mott-Schottky (M-S) plots unveiled a significantly enhanced built-in potential (*V*_*bi*_) in corrosion inhibitor devices relative to their pristine counterpart (1.11 V), with the MTFA device achieving a more pronounced value of 1.16 V. Thereby, providing an expanded depletion region and a formidable driving force for the efficient separation of photo-generated charge carriers, leading to diminished non-radiative recombination losses, which is an indispensable factor underpinning the achievement of elevated open-circuit voltage (*V*_*OC*_) and fill factor (*FF*) in PSCs. This elevated *V*_*bi*_ in corrosion inhibitor devices is primarily attributed to the superior surface potential of the relevant perovskite films as declared by Kelvin probe force microscope (KPFM) images in Supplementary Fig. 37. While the surface potential of TFA (287.34 mV) and MTFA (346.17 mV) films were significantly outclass that of the pristine film (2.93 mV). Indicating a lower effective work function at the perovskite surface as well as a more pronounced upward band bending at the perovskite/electron transport layer interface. Thereby, providing a greater driving force for charge carrier separation and extraction, ultimately leading to the observed increase in *V*_*bi*_.

**Fig. 5:**
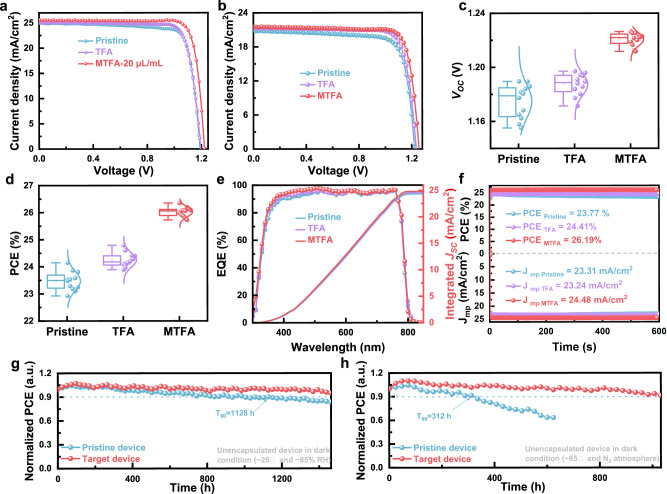
Photovoltaic performance and stability of perovskite solar cells. **a** *J-V* curves of champion PSCs with active area of 0.09 cm^2^. (**b**) *J-V* curves of champion WB PSCs with active area of 0.09 cm^2^. **c** PCE and (**d**) *V*_*oc*_ statistic distributions of 12 independent pristine device and corrosion inhibitor devices. **e** EQE spectrum and (**f**) Steady-state output performance of the pristine device and corrosion inhibitor device. **g** moisture stability and (**h**) thermal stability test result of the pristine device and MTFA device.

In summary, the corrosion inhibitor assessed strategy under investigation has unequivocally pioneered a promising avenue for the preparation of a high-quality perovskite films, distinguished by their compact and dense surface morphology, superior crystallinity, enhanced light absorption properties, and significantly mitigated residual strain, which is realized through the synergistic interplay of cation deprotonation suppression and precisely regulated crystallization dynamic. Thereby, facilitating the achievement of optimized photo-generation carrier extraction and transport as well as hindered non-radiative recombination within the MTFA device. An inverted device featuring the architecture of ITO/NiO_x_/Me-4PACz/Perovskite/PCBM/BCP/Ag was meticulously assembled to comprehensively evaluate the efficacy of corrosion inhibitors on photovoltaic performance optimizing of PSCs. Photovoltaic parameters derived from the *J-V* curves (Fig. 5a) of champion devices are summarized in the Supplementary Table 5, asserting that a supreme PCE of 26.35% was achieved for the MTFA device with short-circuit current (*J*_*SC*_) of 25.62 mA/cm^2^, *V*_*OC*_ of 1.23 V and FF of 83.87%. In contrast, the champion PCE of the pristine and TFA devices were only endowed with 24.15% and 24.79% respectively. Additionally, the statistic distributions of key parameters across 12 independently pristine and MTFA devices are proposed in Fig. 5c, d and Supplementary Fig. 38 to inspect the reproducibility of MTFA in optimizing photovoltaic performance, revealing that all devices have demonstrated commendable repeatability, especially for the corrosion inhibitor assisted devices. Notably, the MTFA device declared the most pronounced parameter aggregation and improvement, with the average PCE of 26.02%. Additionally, external quantum efficiency (EQE) spectra with the cutoff edge of ~800 nm were presented in Fig. 5e to estimate the integrated current of relevant devices, asserting that the integrated current of the pristine/TFA/MTFA devices are estimated as 24.32 mA/cm^2^, 24.57 mA/cm^2^ and 24.81 mA/cm^2^ respectively, aligned well with the values enumerated in Supplementary Table 4. Notably, the MTFA-assisted strategy for optimizing PSCs performance is also applicable to the WB device. As illustrated in Fig. 5b and Supplementary Table 10, a satisfactory PCE of 22.49% was acquired by the MTFA assisted WB PSCs, surpassing that to both the pristine (19.89%) and TFA assisted (21.44%) WB PSCs, confirming the universality of MTFA-assisted strategy in PPS deprotonation suppression and perovskite film crystallization dynamics regulation.

Subsequently, given its pivotal role in hindering commercial viability, the reliability of PSCs in this work was priority elucidated across three perspectives (operational stability, humidity stability and thermal stability). As depicted in Fig. 5f, all corrosion inhibitor assisted devices declared a consistent steady-state output results upon the uninterrupted illumination soaking for 600 s, while the steady-state output performance of the pristine device gradually deteriorated over the extended test period ascribed to the unmitigated trap-induced ion migration. Consequently, the stabilized PCEs were recorded at 23.77% for the pristine device, 24.41% for the TFA device and 26.19% for the MTFA device. Concurrently, humidity stability tests of PSCs in Fig. 5g were also performed under the ISOS-D protocols, with the MTFA device maintaining 94.75% of its initial efficiency after 1464 h, compared to a significant decrease in efficiency of only 83.60% for the pristine one. Similarly, as illustrated in Fig. 5h, the thermal stability of PSCs detected according to the ISOS-T protocols also implies a prominent thermostability of the MTFA device, retaining 91.28% of its initial efficiency after 1032 h of continuous thermal erosion at 85 °C, overwhelming the pristine device, which is disabled after 624 h thermal erosion. Ultimately, operational stability of both the pristine and MTFA devices in Supplementary Fig. 39 was elucidated under the unremitting maximum power point (MPP) tracking in accordance with the ISOS-L protocols for 1032 h. The remarkable operational stability was assigned to the MTFA device, which retained 91.64% of initial efficiency after 1000 h illumination, whereas the value for the pristine device deteriorated to only 77.84%. Additionally, it is worth emphasizing that all stability curves exhibited an initial improvement in PCE during the initial stage of stability testing, followed by a gradual and sustained decline. The underlying mechanisms of this tendency are complex and multifaceted, potentially involving strain relaxation, light/thermal field-induced ion migration, and structural rearrangements^78,79^. Anyway, these findings collectively affirmed the efficacy of MTFA in enhancing device stability through improved crystal quality and increased hydrophobicity.

## Discussion

In summary, a pioneering PPS anti-aging strategy was unveiled in this paper through the innovative introduction of MTFA, which has been rigorously substantiated to inhibit the cation deprotonation within PPS through a moderately hydrolytic process, thereby extending the shelf life of PPSs to exceeding 22 days. Notably, the carboxyl functional groups present in the byproduct of TFA exhibit a pronounced propensity to impede the deprotonation of organic cations as well as accompanied side reactions, thereby realizing a significantly prolonged shelf life of PPS. Additionally, the remarkable binding affinity occurring between -COOCH_3_ or its accompanying -COOH groups and uncoordinated Pb^2+^ ions also unveiled a compelling and highly effective efficacy in the regulation of perovskite crystallization dynamic. This synergistic interplay has pioneered a promising avenue for the preparation of high-quality perovskite films with compact and dense surface morphology, superior crystallinity, enhanced light absorption properties, significantly mitigated residual strain and optimized carrier dynamic. Therefore, it leads to a splendid PCE of 26.35% with conspicuous perovskite stability for the MTFA device, which retained 94.75% of its initial efficiency after 1464 h of humidity erosion.

## Methods

### Materials

Organic compound of formamidinium iodide (FAI, 99.99%, trace elements basis), methylammonium chlorine (MACl, 99.99%, trace elements basis), methylammonium bromine (MABr, 99.99%, trace elements basis) and methylammonium iodide (MAI, 99.99%, trace elements basis); inorganic compound of Lead (II) iodide (PbI2, 99.99%, trace metals basis) and Cesium iodide (CsI, 99.999%, AB 109298) were all purchased from Xi’an Yuri Solar Co., Ltd and used without further purification. Additive materials of trifluoroacetic acid (TFA, 99.0%) and methyl trifluoroacetate (MTFA, 99.0%); Charge transport material of self-assembly molecule [4-(3,6-dimethoxy-9H-carbazole-9-yl)butyl] phosphonic acid (Me-4PACz, >98.0%) was purchased from Shanghai Macklin Biochemical Technology Co., Ltd; Nickel oxide (NiO_x_, 99.999%), Lead thiocyanate (Pb (SCN)_2_, 99.98%), [6,6]-phenyl-C61-butyric acid methyl ester (PC61BM, 99%) and Bathocuproine (BCP, 99.8%) were purchased from Advanced Electron Technology Co., Ltd; Chlorobenzene (CB, 99.8%), 2-propanol (IPA, 99.5%), N-methylpyrrolidone (NMP, 99.9%), Dimethyl sulfoxide (DMSO, 99.9%) and *N*,*N*-dimethylformamide (DMF, 99.9%) were purchased from Sigma Aldrich. Silver shots (Ag, 2-3 mm, 99.999%) was bought from Alfa.

### Precursor Solution Preparation

The perovskite precursor solution was prepared by dissolving 774.48 mg PbI_2_, 256.17 mg FAI, 20.78 mg CsI, 4.83 mg MAI, 7.75 mg MABr, 32.41 mg MACl in 1 mL mixed solvent of DMF/DMSO (4:1 in v:v). Additives contain TFA and MTFA are also incorporated in this stage with the concentration of 20 μL/mL respectively. During this process, the mixed solutions were stirred overnight in a shaker with 75 °C. Resultant solutions were filtered through a 0.45 μm hydrophobic PTFE filter before use.

### Wide bandgap precursor solution preparation

The wide bandgap perovskite precursor solution was prepared by dissolving 10.00 mg Pb (SCN)_2_, 479.42 mg PbI_2_, 145.37 mg FAI, 50.75 mg CsI, 29.16 mg MABr, 95.43 mg PbBr_2_ in 1 mL mixed solvent of DMF/NMP (4:1 in v:v). Additives contain MTFA are also incorporated in this stage with the concentration of 20 μL/mL. During this process, the mixed solutions were stirred overnight in a shaker with 75 °C. Resultant solutions were filtered through a 0.45 μm hydrophobic PTFE filter before use.

### Perovskite solar cell fabrication

The planer PSCs were fabricated on pre-patterned ITO glass substrates (10 Ω per square, around 2 × 2.5 cm^2^ in size). The ITO glass substrates were cleaned with 5% decon-90 solution, de-ionized water, alcohol at 50 °C for 20 min, respectively. Then the ITO substrates were dried with nitrogen and cleaned in a UV-ozone for 30 min. Typically, The NiO_x_ solution (10 mg/mL in deionized water and isopropanol (3:1 v/v)) was spin-coated on the ITO glass substrates at 3000 rpm/s for 30 s. 100 μL Me-4PACz (0.335 mg/mL) dissolved in absolute ethanol was then spin-coated using a speed of 3000 rpm/s for 30 s with an acceleration of 500 rpm/s, followed by annealing at 100 °C. 100 μL perovskite precursor was applied into Me-4PACz layer for subsequently, the dispersed perovskite solution was spin-coated with 1000 rpm/s for 10 s and then 5000 rpm/s for 30 s. At 8 s before the end of the procedure, 200 μL chlorobenzene as the antisolvent was dripped into the precast film surface respectively. After that, the substrates were quickly transferred to a hot plate with 100 °C for 30 min annealing. Then, starting the program with 3000 rpm/s for 30 s with a ramp of 3000 rpm/s, 20 mg/mL PC_61_BM in chlorobenzene was spin-coated on the obtained perovskite layers after 3 s to acquire the electron selective contact. Sequentially, 8 nm BCP controlled by temperature and 100 nm Ag electrode via controlling power were thermally deposited on the PC_61_BM layer with a shadow mask, using a thin-film evaporation system under a high vacuum lower than 1 × 10^−6^ mbar. Ultimately, the PSCs with defined areas of 9 mm^2^ on each pixel were obtained.

### Device characterization and theoretical calculations

The TOF-SIMs was performed on TOF-SIMS 5-100 (ION-TOF GmbH). Bi^3+^ (30 keV) was used to analyze deeply about the sample with an analysis area of 150 × 150 μm^2^. NMR spectra were recorded with a Bruker AVANCE 400 instrument operating at 400 MHz at 298 K. In-situ UV-Vis absorption and In-situ PL mappings are tested by an in-situ dynamic spectrometer (Shaanxi Puguangweishi Technology Co.,Ltd, STU-300). AFM and KPFM images were measured by a Nanoscope V Dimension Icon AFM system (Bruker, Santa Barbara, CA). UV–vis absorption/transmittance spectra were acquired from a spectrophotometer (U-4100, Hitachi). XPS tests were performed on a surface analysis system (ESCALAB 250Xi, Thermo Fisher). The X-ray absorption spectroscopy (XAFS) study was performed at the BL14B2 of SPring-8 (2.5 GeV, 150 mA), Japan, the X-ray beam was mono-chromatized with InSb (111) double-crystal monochromator, to obtain X-ray adsorption fine structure (XAFS) spectra both in near and extended edge. SEM images were tested by a Helios NanoLab G3 SEM system. XRD patterns were collected by a x′pert3 powder X-ray diffractometer (PANalytical, Netherland), and GIXRD measurements were conducted using a PANalytical Empyrean, which both operated at 40 kV and 40 mA with a Cu Kα X-ray source. Steady-state PL and TRPL curves were measured using a FluoTime 300 spectrometer (PicoQuant, German) with wavelength of 532 nm, fluence of 2.38 μJ/cm^2^ and repetition rate of 80 MHz. PL mapping was performed on an inVia confocal Raman microscope (Renishawplc.). Light *J-V* curves were measured under ambient air conditions: ~101.3 kPa, 20 ~ 25°C and ~40% RH, by a Keithley 2450 source meter under simulated AM 1.5 G illumination (100 mW cm^−2^) generated by a sunlight simulator (Oriel 92251A-1000), which was calibrated by a NREL-traceable KG5 filtered silicon reference cell. The scan range was set from 1.3 V to −0.2 V with a 0.02 V bias step and a 20 ms delay time. EQE spectra were characterized by a 150 W xenon lamp (Oriel) equipped with a monochromator (Cornerstone 74004). Dark *J-V* curves were measured by a Keithley 2636 source meter. TPC measurements were fulfilled by a home-built system. The sample was excited by a 532 nm pulse laser (1000 Hz, 3.2 ns), and the photocurrent decay process was recorded by a digital oscilloscope (Tektronix, D4105) with a sampling resistor of 50 Ω. TPV tests were carried out on the same system, while the samples were excited by a 405 nm pulse laser (100 Hz, 10 ms) and the sampling resistor of the digital oscilloscope was adjusted to be 1 MΩ. EIS and M–S plots were measured by an electrochemical workstation (CHI660B) under dark condition. During the EIS tests, a forward voltage bias of 1 V was applied to the samples with the frequency sweeping from 1 MHz to 100 Hz. Karl Fischer moisture analysis was performed by a Karl Fischer moisture Titrator (Metrohm-852) with coulometric method and volumetric method under ~25 °C and ~35% RH. A 2 × 2 × 2 periodic supercell model with a 20 Å vacuum layer was constructed to model the (001) Pb–I and FA–I terminated surfaces of cubic-phase FAPbI_3_. Density functional theory (DFT) calculations were performed using the Vienna Ab initio Simulation Package (VASP). The generalized gradient approximation (GGA) with the Perdew–Burke–Ernzerhof (PBE) functional was adopted as the exchange-correlation potential. Both lattice parameters and atomic positions were fully relaxed until the energy convergence criterion of 10^−6 ^eV and the force threshold of 10^−3 ^eV/Å were satisfied. A kinetic energy cutoff of 500 eV was used within the projector augmented wave (PAW) method to model the electron-ion interactions. The Brillouin zone was sampled using a *Γ*-centered *k*-point mesh with a spacing of approximately 0.15 Å^−1^.

### Reporting summary

Further information on research design is available in the Nature Portfolio Reporting Summary linked to this article.

## Supplementary information

Supplementary Information

Reporting Summary

Transparent Peer Review file

## Source data

Source data

## Data Availability

All data supporting the findings of this study are available within the article and its Supplementary Information files.  Source data are provided with this paper.
