# Supplementary material for: Deprotonation suppressing via competitive proton transfer control for efficient perovskite solar cells
Source: Nat Commun. 2026 May 25;17:6829. doi: 10.1038/s41467-026-73620-0 (PMC13389051; doi:10.1038/s41467-026-73620-0)
Supplement: Supplementary file 1 — Supplementary Information [file 41467_2026_73620_MOESM1_ESM.pdf]

Deprotonation Suppressing via Competitive Proton Transfer Control for Efficient Perovskite Solar Cells

Hang Dong[1,2], Jinsong Qu[1], Songya Wang[1], Dazheng Chen[1*], Wenming Chai[1], Weidong Wang[2], Weidong Zhu[1], He Xi[3], Long Zhou[3], Jincheng Zhang[1], Pengfei Huang[4*], Yue Hao[1] and Chunfu Zhang[1*]

1. State Key Laboratory of Wide-Bandgap Semiconductor Devices and Integrated Technology, Faculty of Integrated Circuit, Xidian University, Xi'an 710071, People's Republic of China
2. School of Mechano-Electronic Engineering, Xidian University, Xian 710071, People's Republic of China
3. School of Advanced Materials and Nanotechnology, Xidian University, Xian 710071, People's Republic of China
4. PetroChina Shenzhen New Energy Research Institute Co., Ltd., Shenzhen 518063, People's Republic of China

Corresponding authors: dzchen@xidian.edu.cn, hpf01@petrochina.com.cn, cfzhang@xidian.edu.cn

Electronic Supplementary Information (ESI) available: ^{1}H NMR spectra, pH test results of PPSs, Statistical results, *J-V* characteristics, Tables, etc.

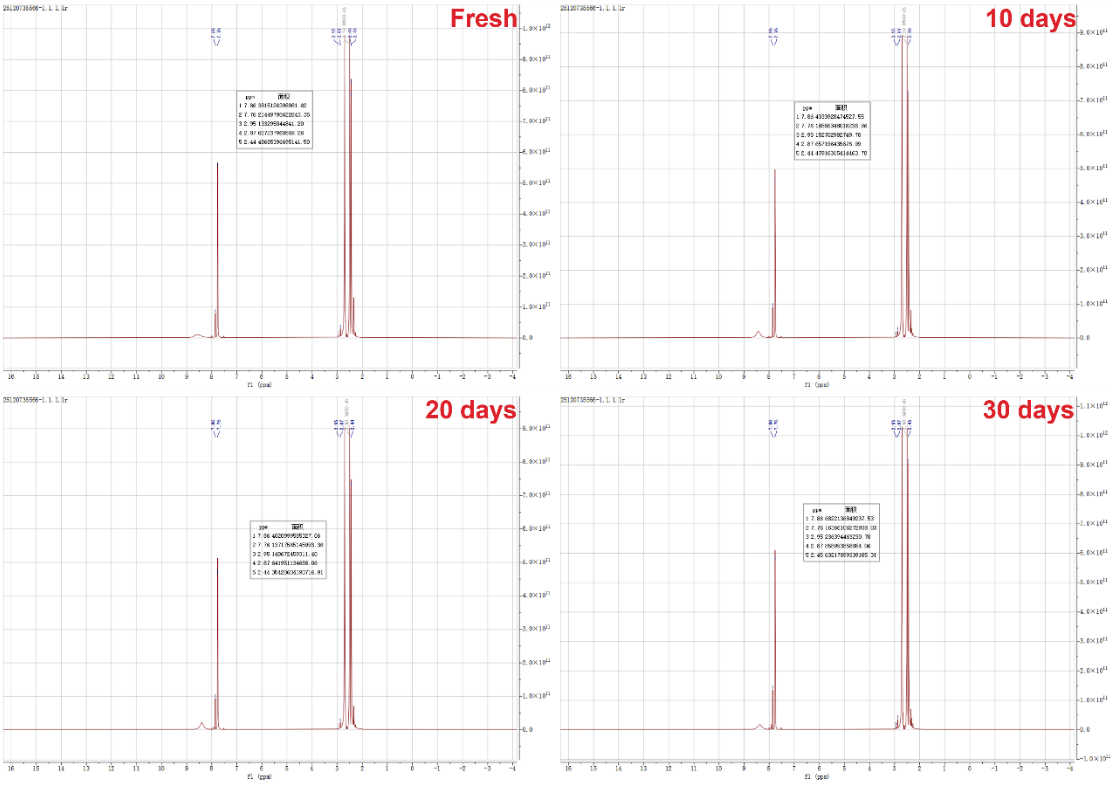

Supplementary Figure 1. ^{1}H NMR spectra of the fresh and aged pristine PPS (DMSO-d_6) at different aging periods.

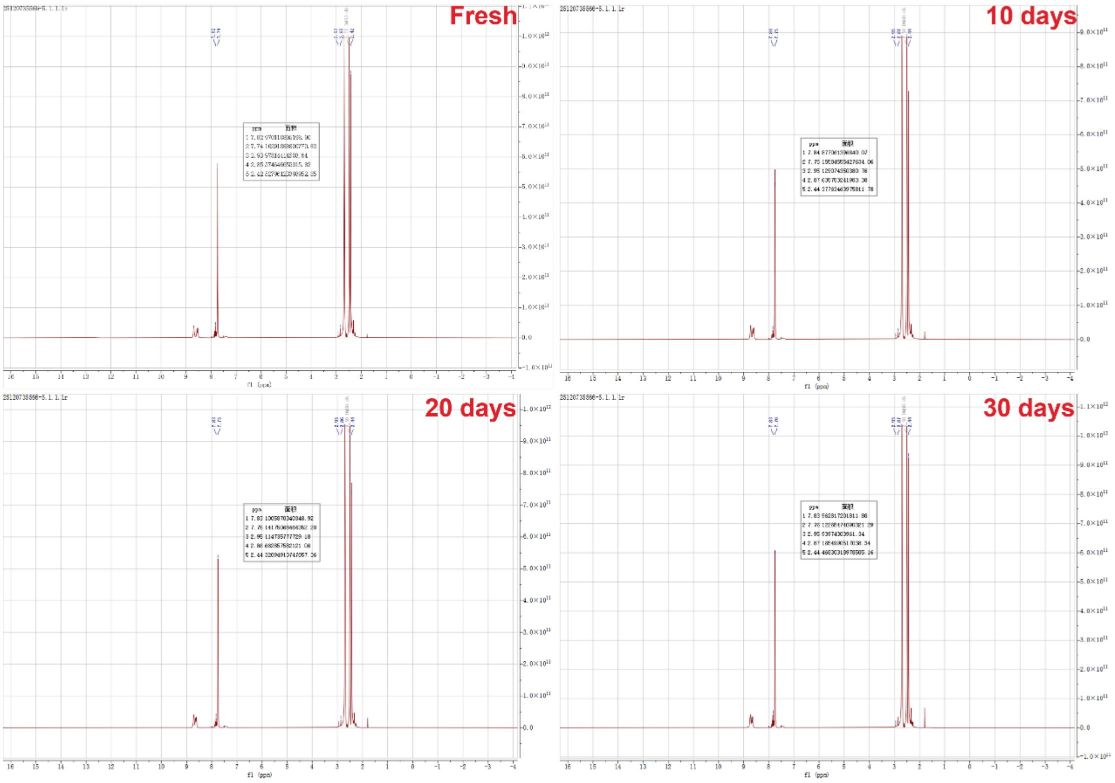

Supplementary Figure 2. ^{1}H NMR spectra of the fresh and aged TFA PPS (DMSO-d_6) at different aging periods.

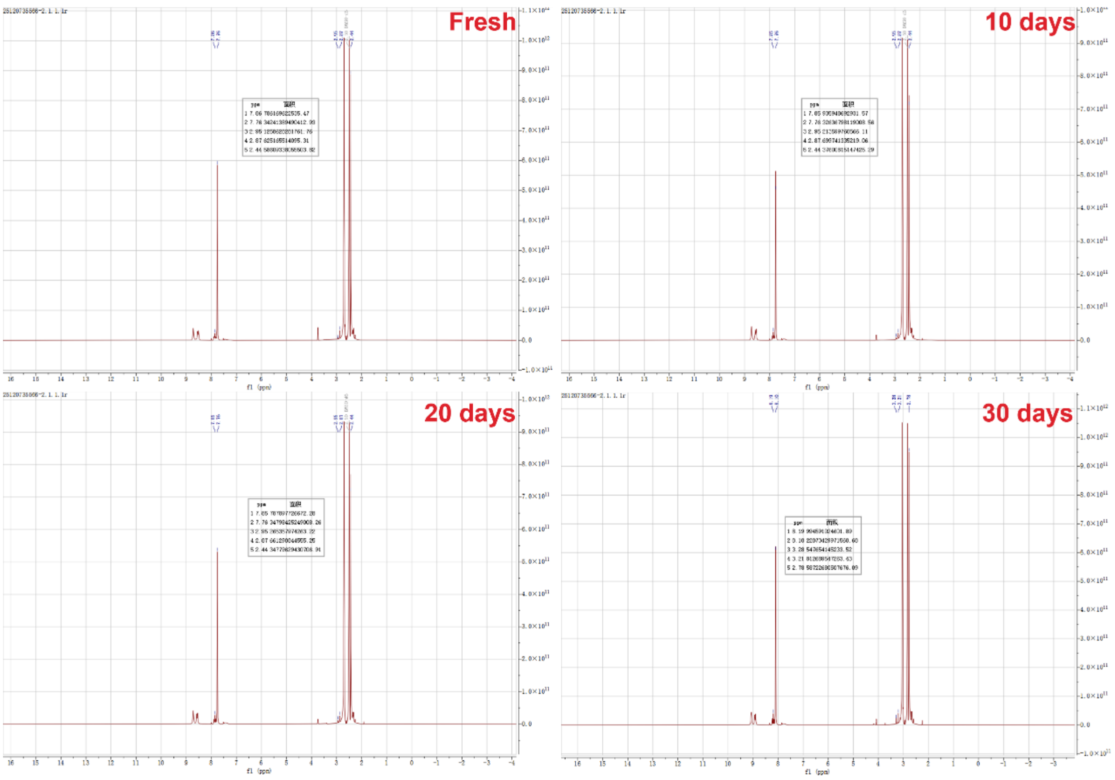

Supplementary Figure 3. ^{1}H NMR spectra of the fresh and aged 10 μL/mL MTFA PPS (DMSO-d_6) at different aging periods.

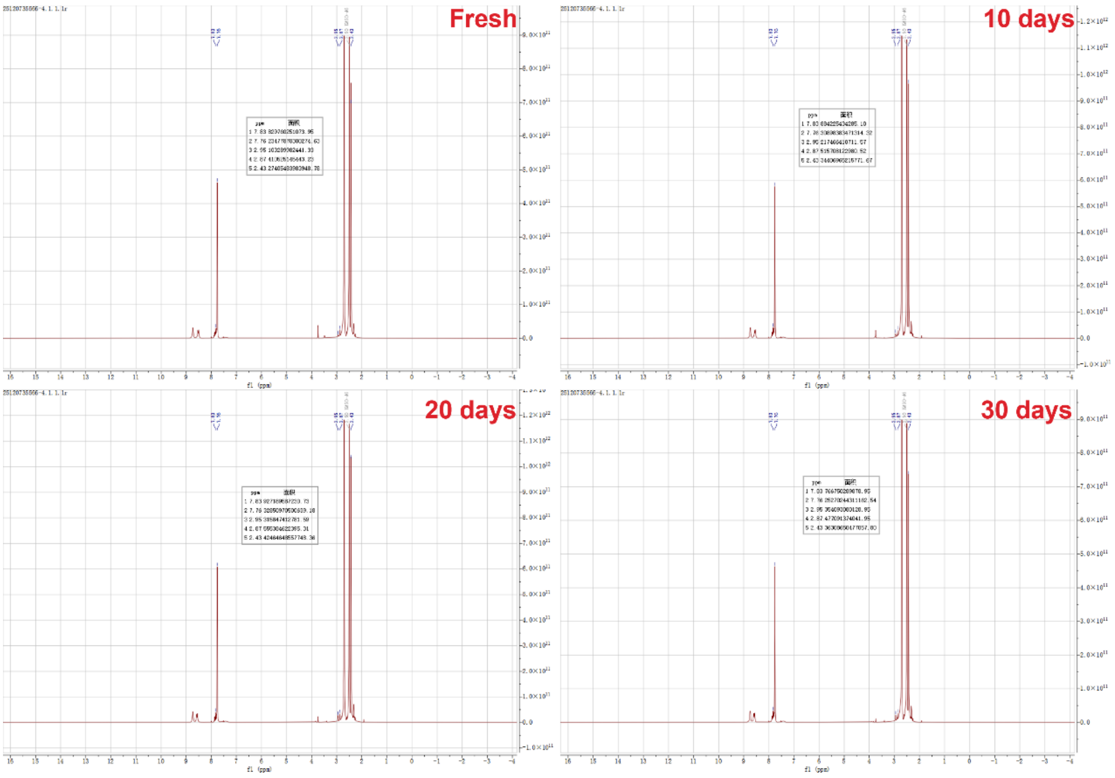

Supplementary Figure 4. ^{1}H NMR spectra of the fresh and aged 20 μL/mL MTFA PPS (DMSO-d_6) at different aging periods.

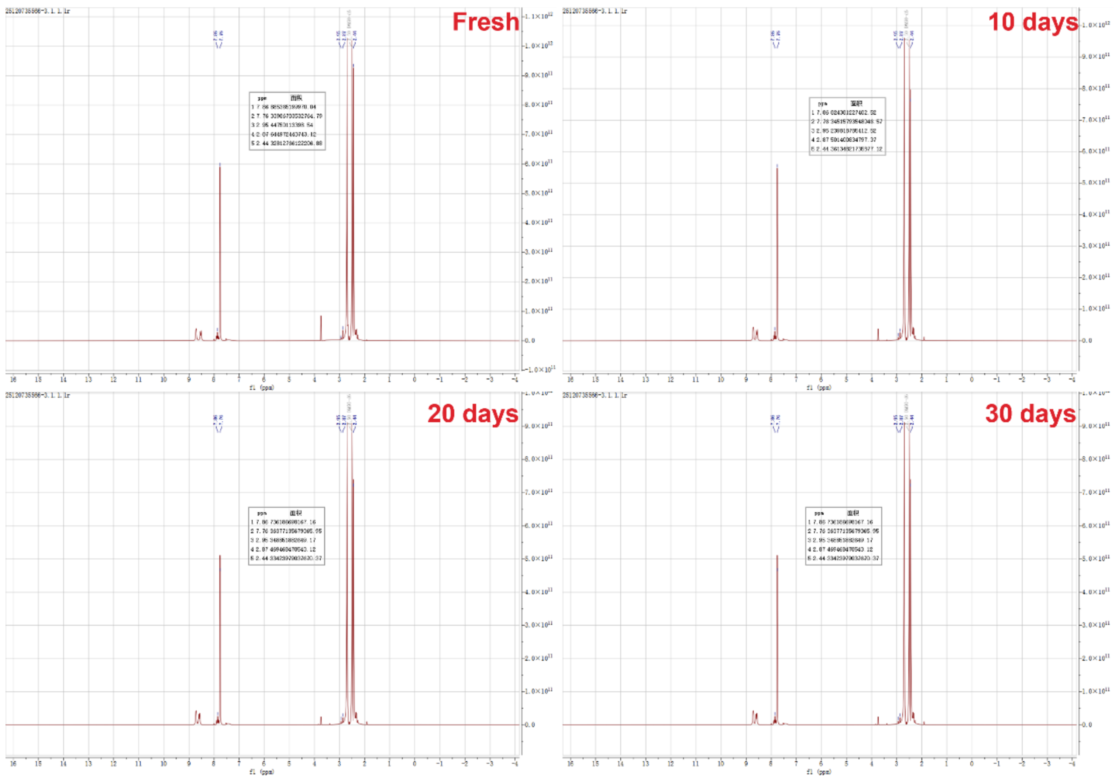

Supplementary Figure 5. ^{1}H NMR spectra of the fresh and aged 30 µL/mL MTFA PPS (DMSO-d_6) at different aging periods.

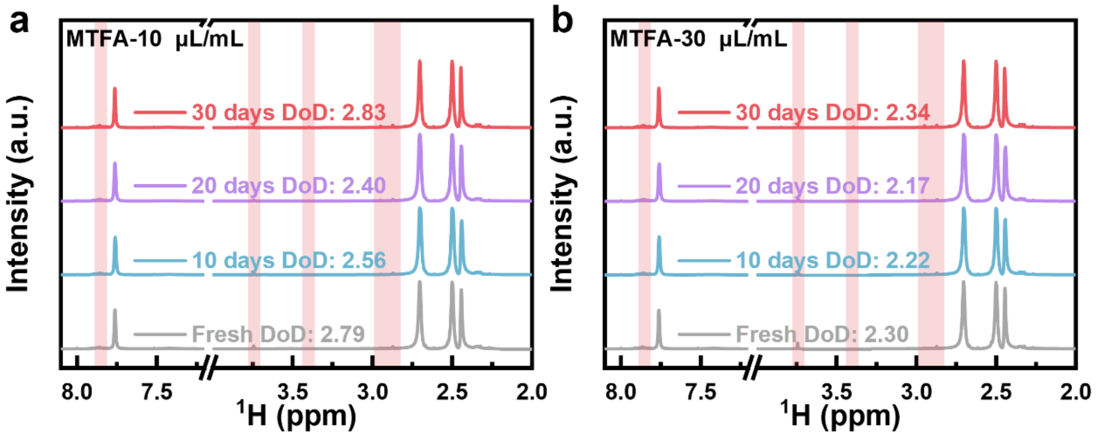

Supplementary Figure 6. ^{1}H NMR spectra of **(a)** 10 µL/mL MTFA and **(b)** 30 µL/mL MTFA PPSs at different aging periods, with all spectra normalized to the intensity of the DMSO-d_6 signal as an internal reference.

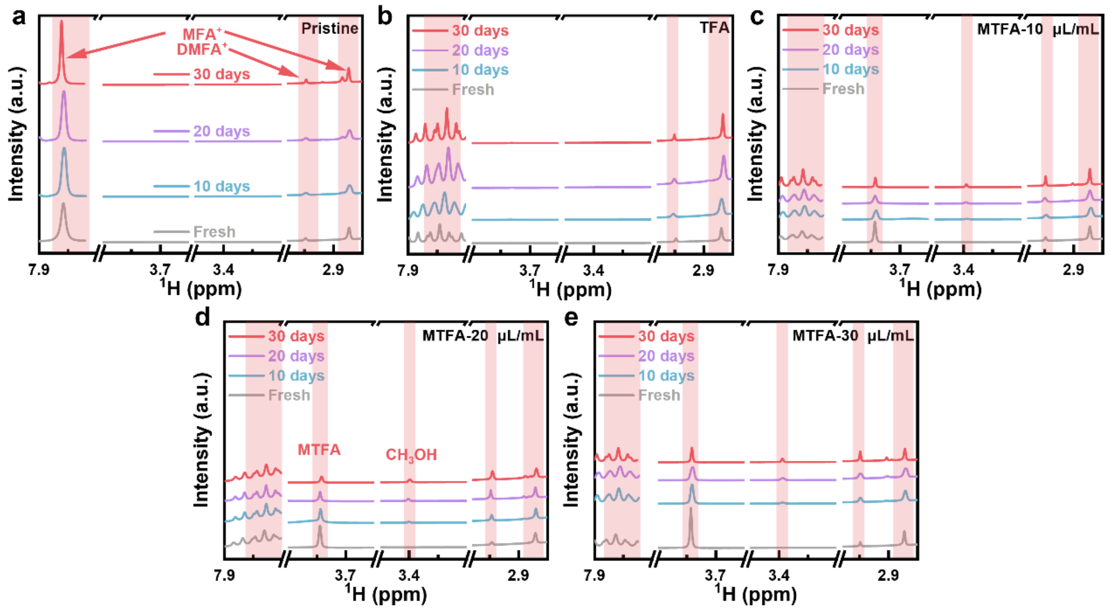

Supplementary Figure 7. The partial enlarged ^{1}H NMR spectra indexed to MFA^{+}, $DMFA^{+}$, MTFA and CH_3OH signals of the fresh and aged **(a)** pristine, **(b)** TFA-doped, **(c)** 10 μL/mL MTFA-doped, **(d)** 20 μL/mL MTFA-doped and **(e)** 30 μL/mL MTFA-doped PPSs (DMSO-d_6), with all spectra normalized to the intensity of the DMSO-d_6 signal as an internal reference.

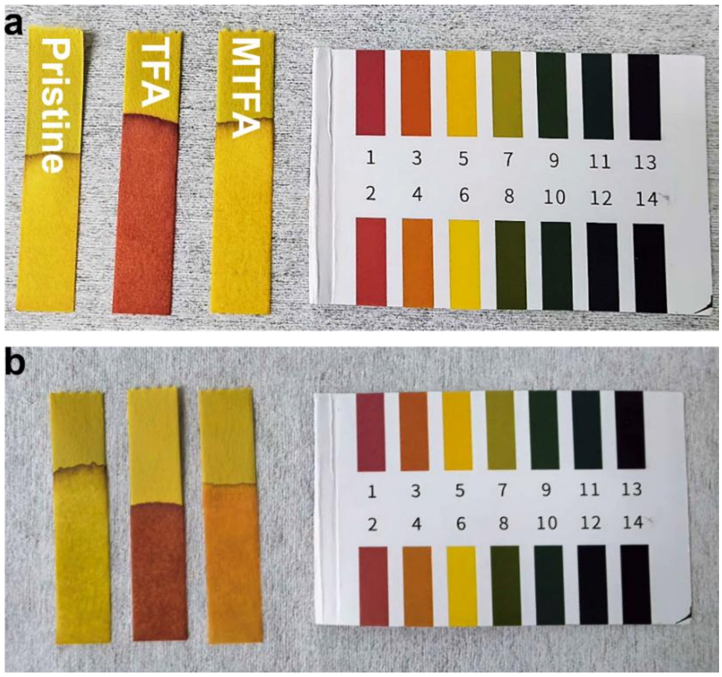

Supplementary Figure 8. The pH tests of the **(a)** fresh and **(b)** aged pristine/TFA/MTFA-doped PPS, with both TFA and MTFA doped at a concentration of 20 μL/mL.

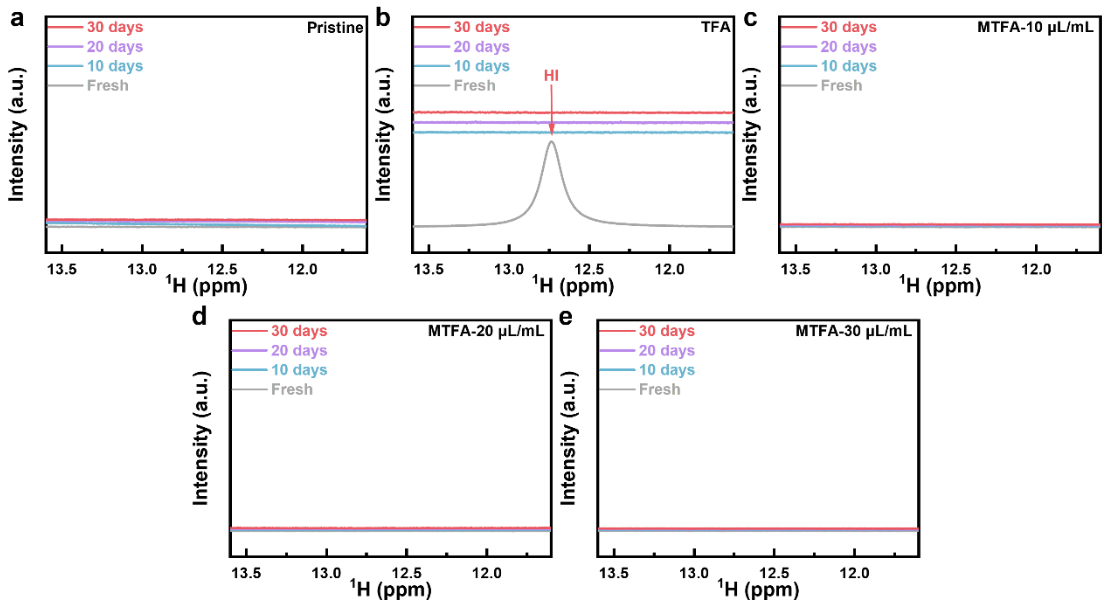

Supplementary Figure 9. The partial enlarged ^{1}H NMR spectra indexed to the HI signal of the **(a)** pristine, **(b)** TFA-doped, **(c)** 10 μL/mL MTFA-doped, **(d)** 20 μL/mL MTFA-doped and **(e)** 30 μL/mL MTFA-doped PPSs (DMSO-d_6).

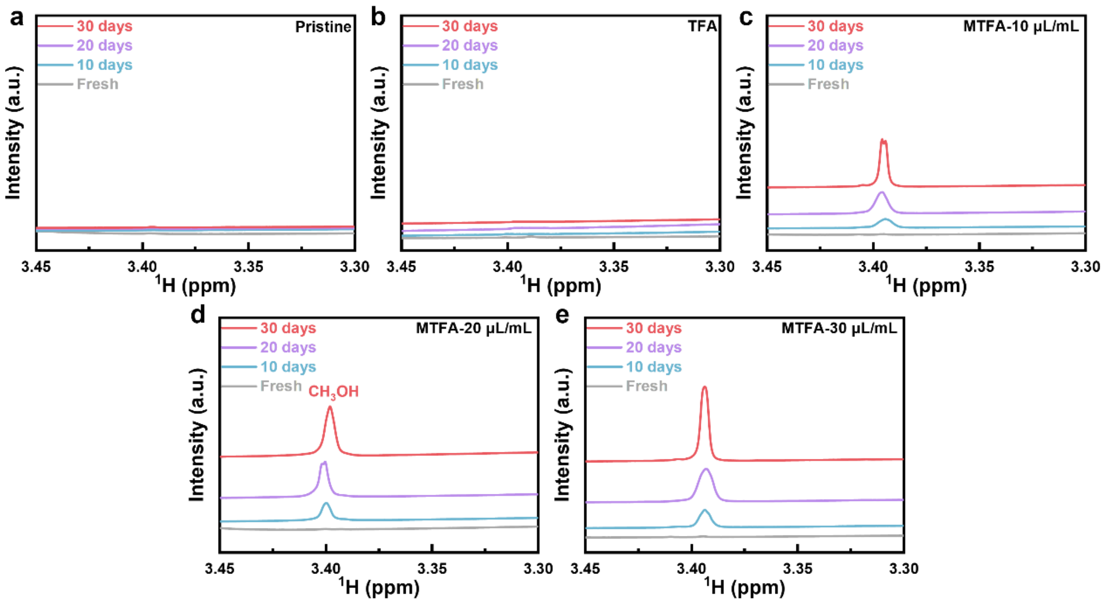

Supplementary Figure 10. The partial enlarged ^{1}H NMR spectra indexed to the CH_3OH of the **(a)** pristine, **(b)** TFA-doped, **(c)** 10 μL/mL MTFA-doped, **(d)** 20 μL/mL MTFA-doped and **(e)** 30 μL/mL MTFA-doped PPSs (DMSO-d_6).

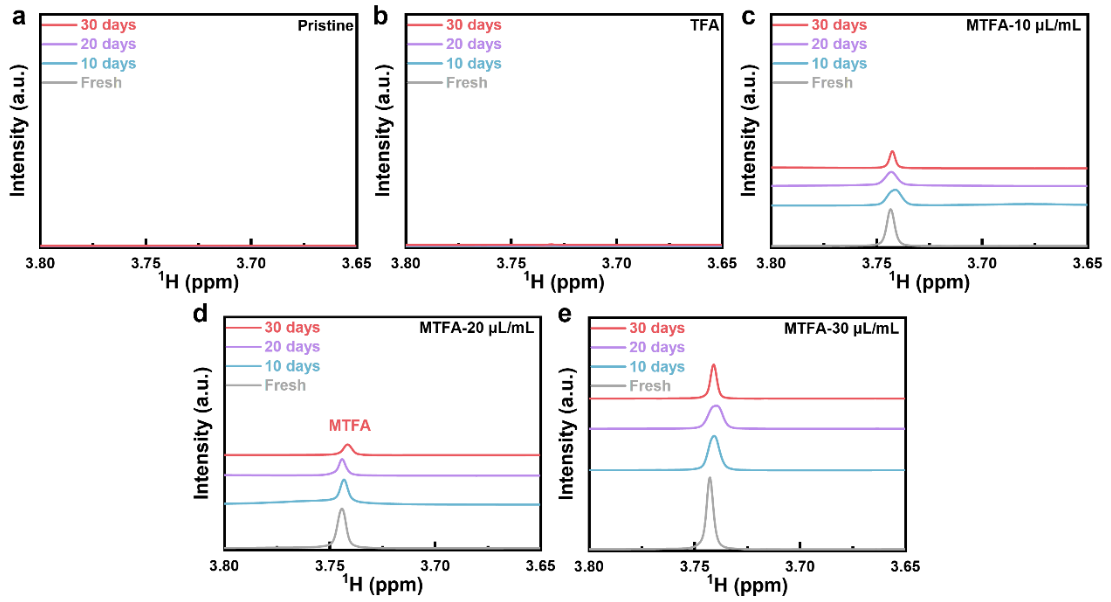

Supplementary Figure 11. The partial enlarged ^{1}H NMR spectra indexed to the MTFA signal of the **(a)** pristine, **(b)** TFA-doped, **(c)** 10 μL/mL MTFA-doped, **(d)** 20 μL/mL MTFA-doped and **(e)** 30 μL/mL MTFA-doped PPSs (DMSO-d_6).

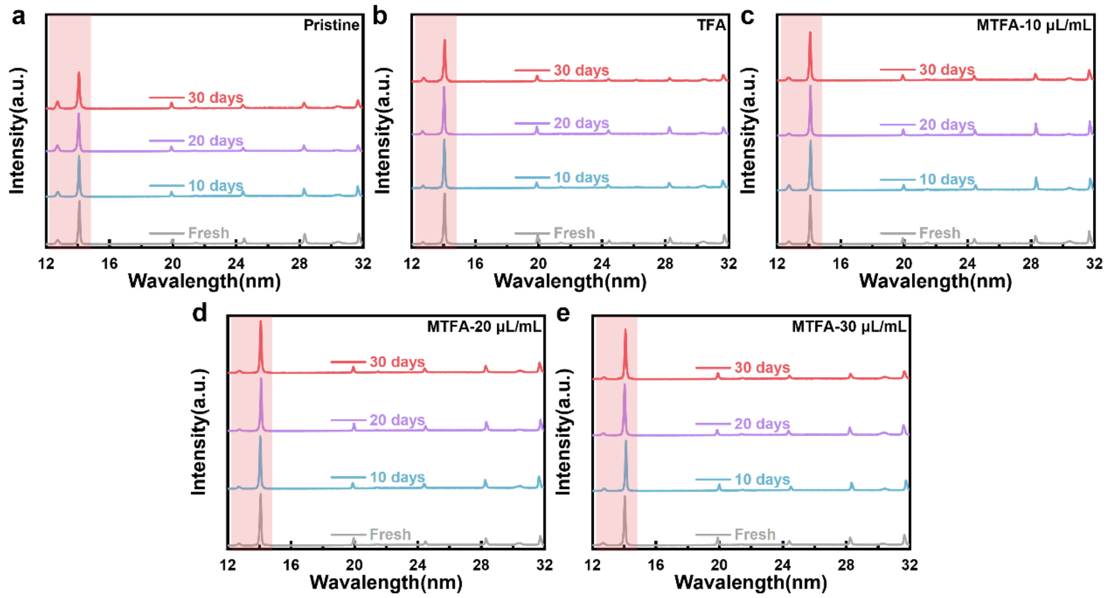

Supplementary Figure 12. XRD patterns evolution of perovskite films prepared from fresh or aged **(a)** pristine, **(b)** TFA-doped, **(c)** 10 μL/mL MTFA-doped, **(d)** 20 μL/mL MTFA-doped and **(e)** 30 μL/mL MTFA-doped PPSs as a function of aging time.

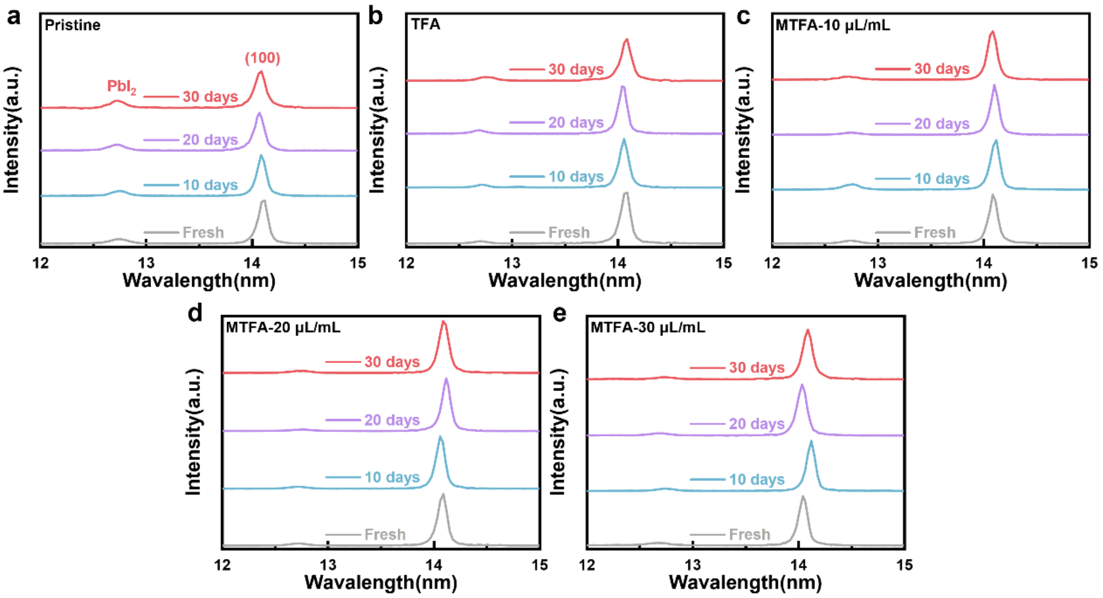

Supplementary Figure 13. Partially enlarged PbI_2 and (100) diffraction peaks of perovskite films prepared from fresh or aged **(a)** pristine, **(b)** TFA-doped, **(c)** 10 µL/mL MTFA-doped, **(d)** 20 µL/mL MTFA-doped and **(e)** 30 µL/mL MTFA-doped PPSs as a function of aging time.

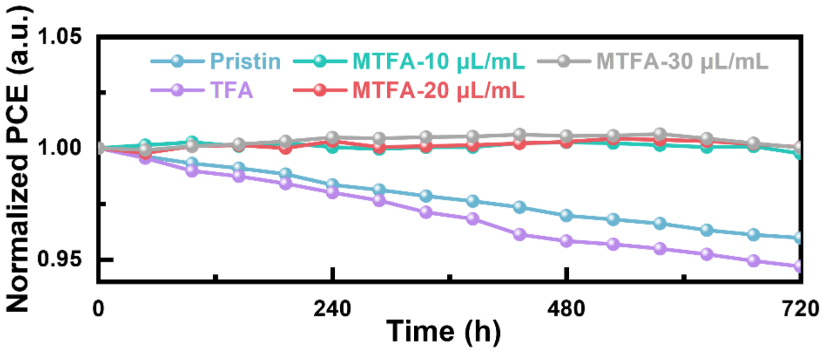

Supplementary Figure 14. The PCE evolution trend of PSCs with the extend of aging time.

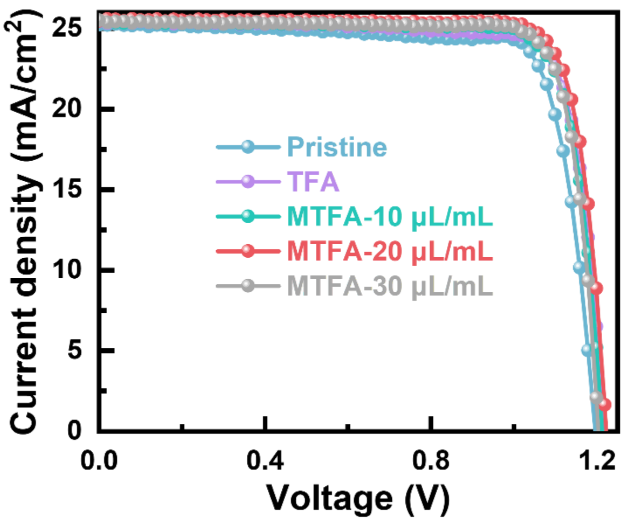

Supplementary Figure 15. *J-V* curves of champion PSCs with freshly prepared PPSs.

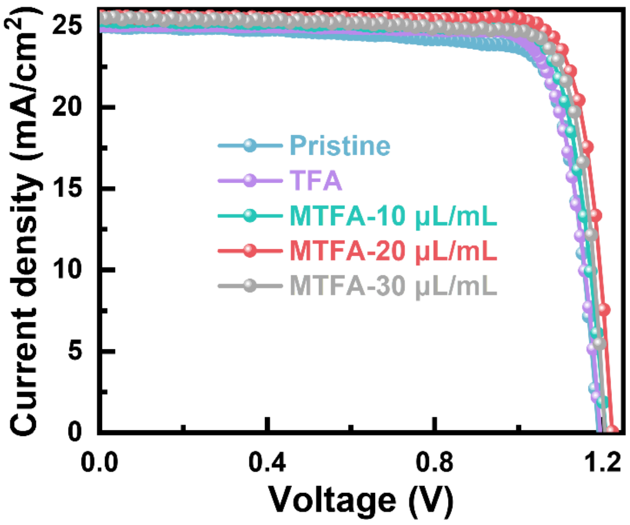

Supplementary Figure 16. *J-V* curves of champion PSCs with aging PPSs.

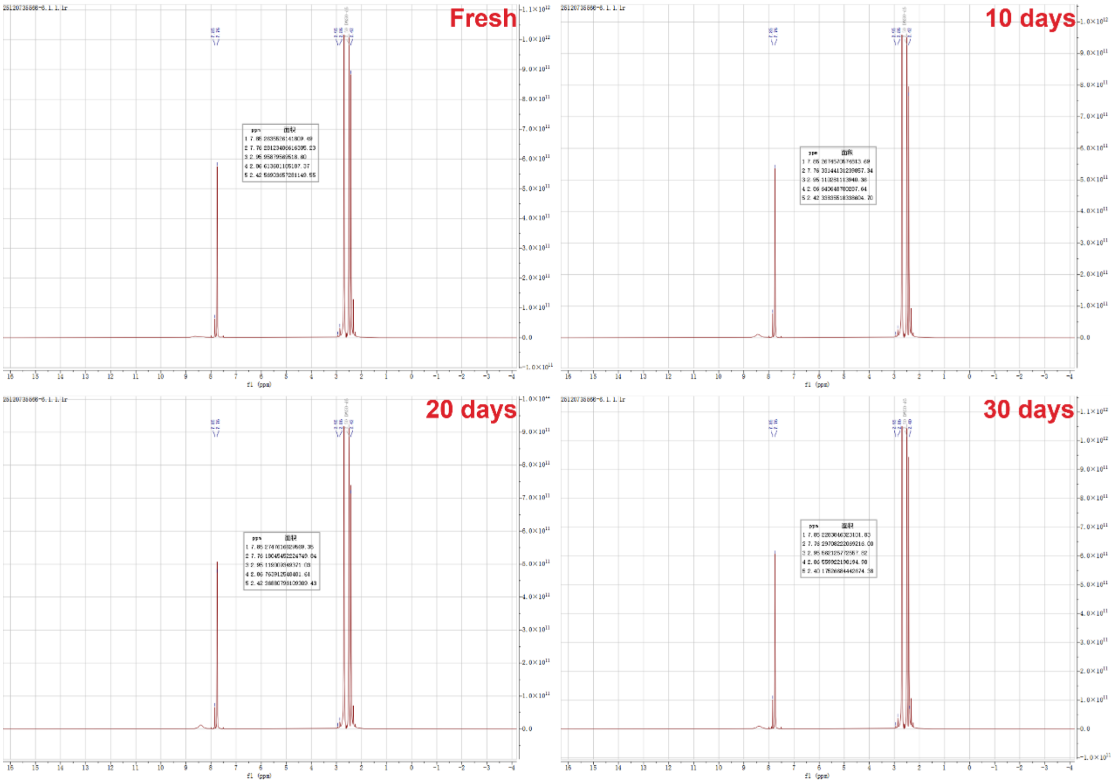

Supplementary Figure 17. ^{1}H NMR spectra of the fresh and aged pristine WB PPS (DMSO-d_6) at different aging periods.

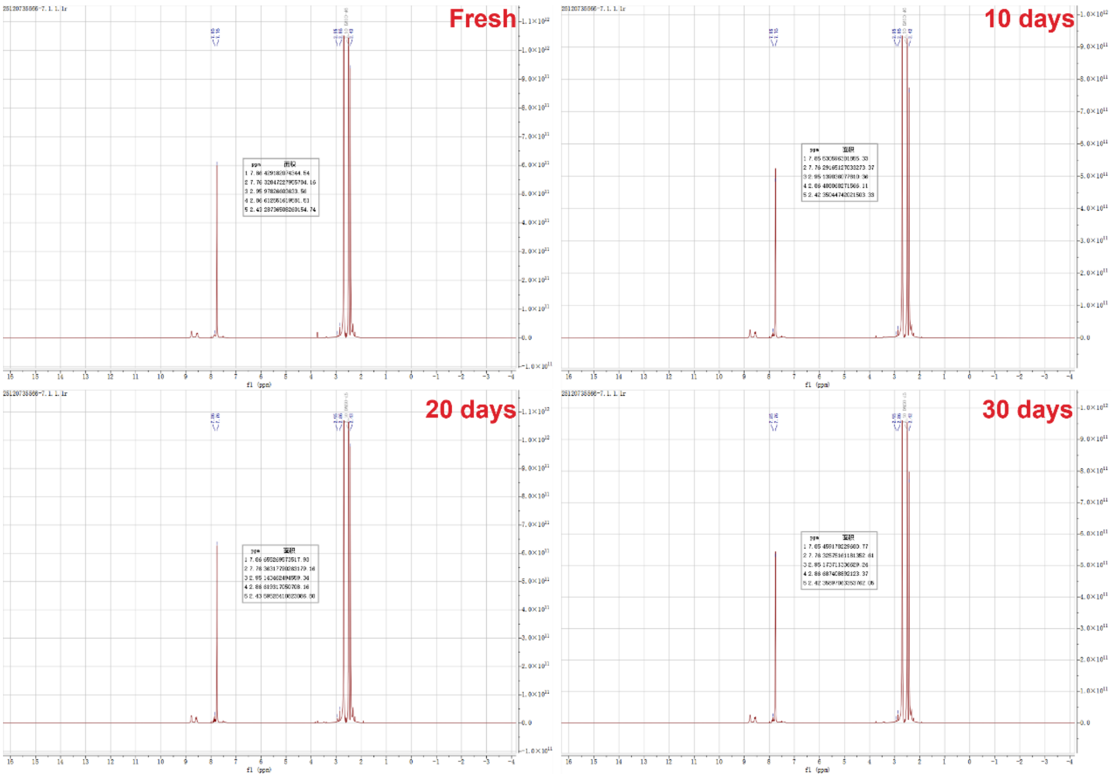

Supplementary Figure 18. ^{1}H NMR spectra of the fresh and aged 20 μL/mL MTFA-doped WB PPS (DMSO-d_6) at different aging periods.

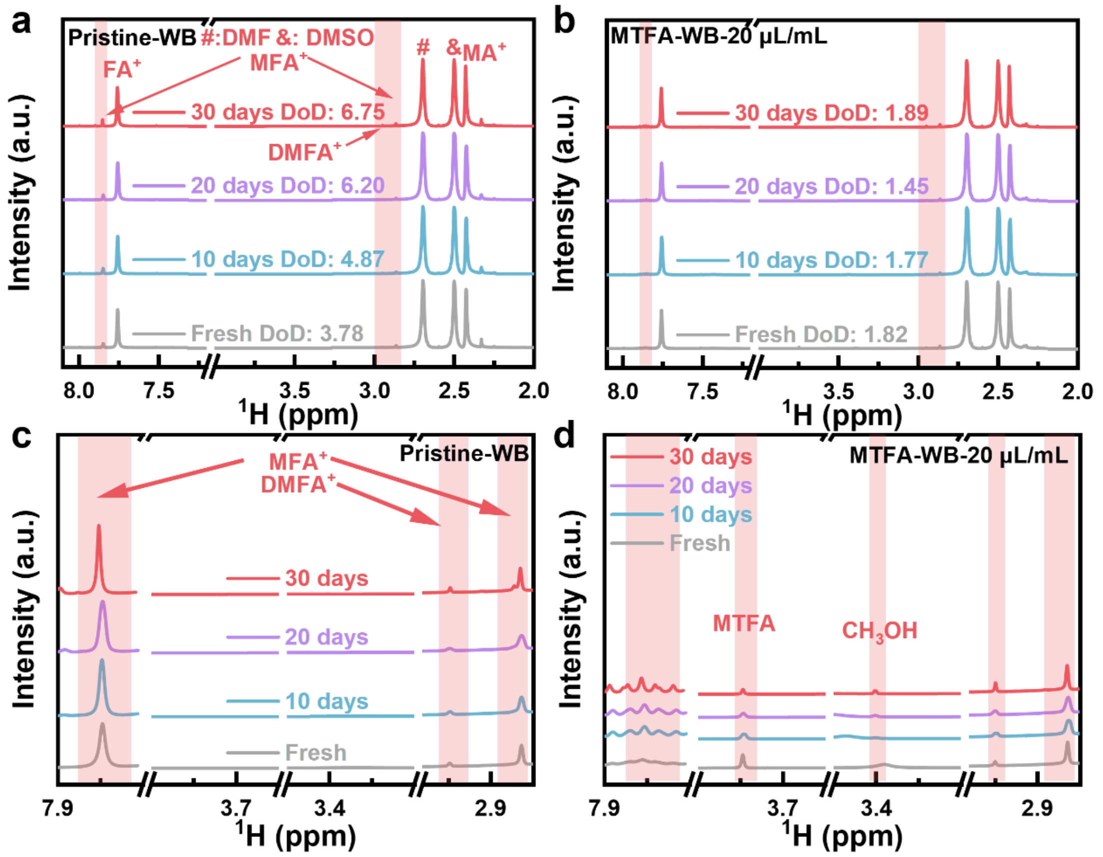

Supplementary Figure 19. ^{1}H NMR spectra of **(a)** pristine and **(b)** 20 µL/mL MTFA-doped WB PPSs at different aging periods, with all spectra normalized to the intensity of the DMSO-d_6 signal as an internal reference. The partial enlarged ^{1}H NMR spectra indexed to MFA^+, $DMFA^+$, MTFA and CH_3OH signals of the fresh and aged **(c)** pristine and **(d)** 20 µL/mL MTFA-doped WB PPSs (DMSO-d_6), with all spectra normalized to the intensity of the DMSO-d_6 signal as an internal reference.

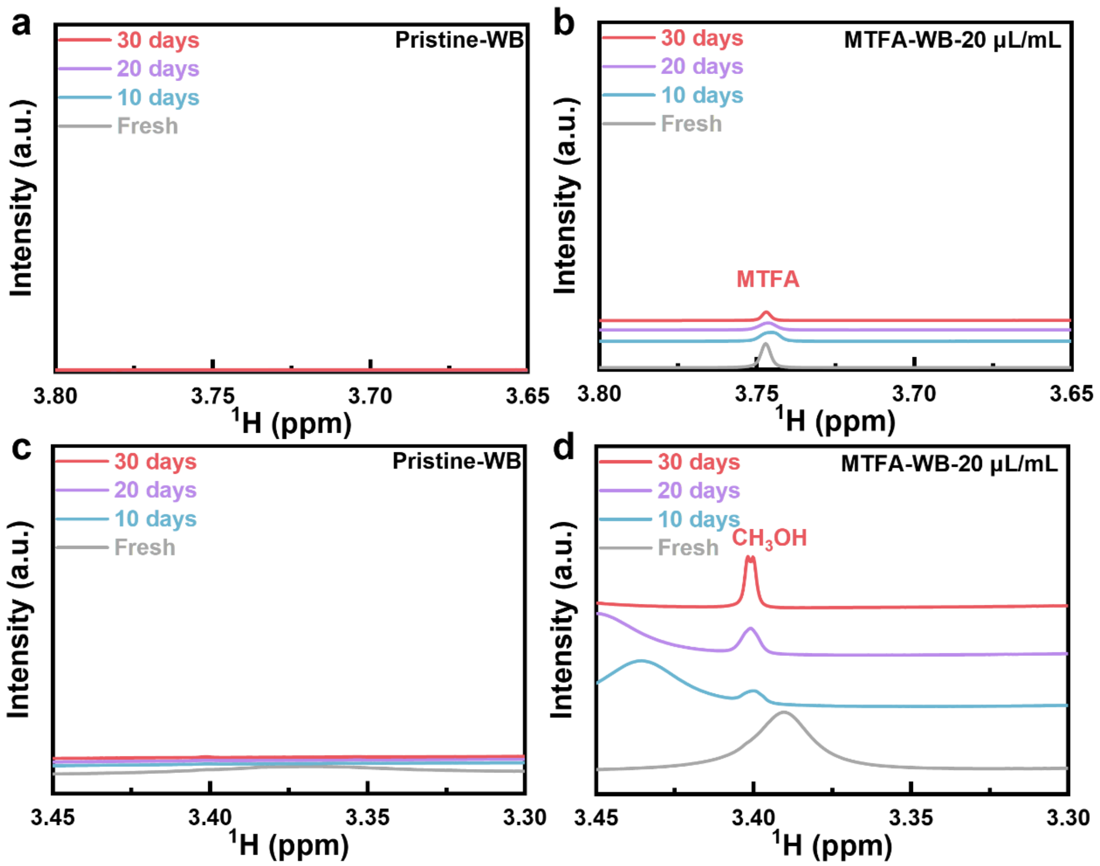

Supplementary Figure 20. The partial enlarged ^{1}H NMR spectra indexed to the MTFA of the **(a)** pristine and **(b)** 20 µL/mL MTFA-doped WB PPSs (DMSO-d$_6$). The partial enlarged ^{1}H NMR spectra indexed to the CH_3OH of the **(c)** pristine and **(d)** 20 µL/mL MTFA-doped WB PPSs (DMSO-d$_6$).

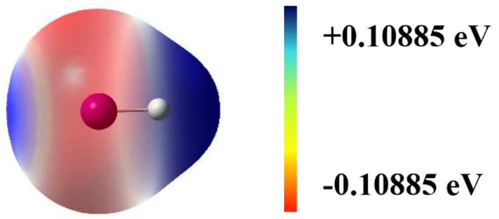

Supplementary Figure 21. Electrostatic potential distribution (ESP) of HI.

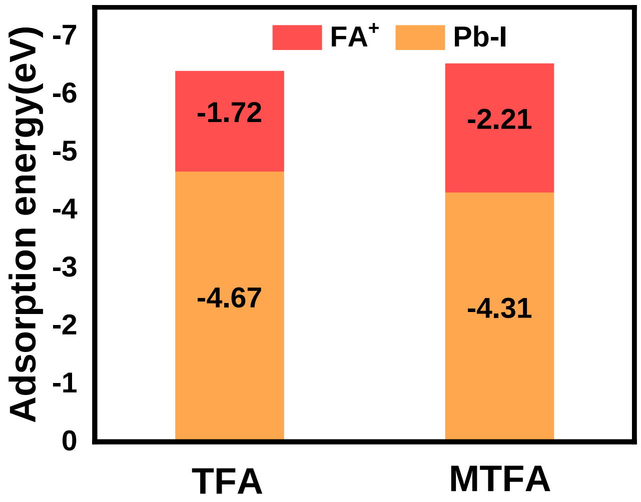

Supplementary Figure 22. The adsorption energy between TFA/MTFA and the Pb-I cluster structure or FA$^+$ calculated from DFT results.

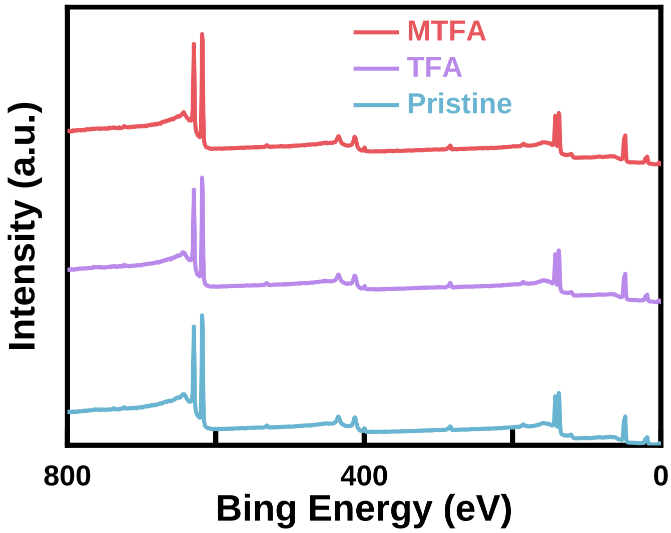

Supplementary Figure 23. Integrated XPS spectra of perovskite films with various corrosion inhibitors.

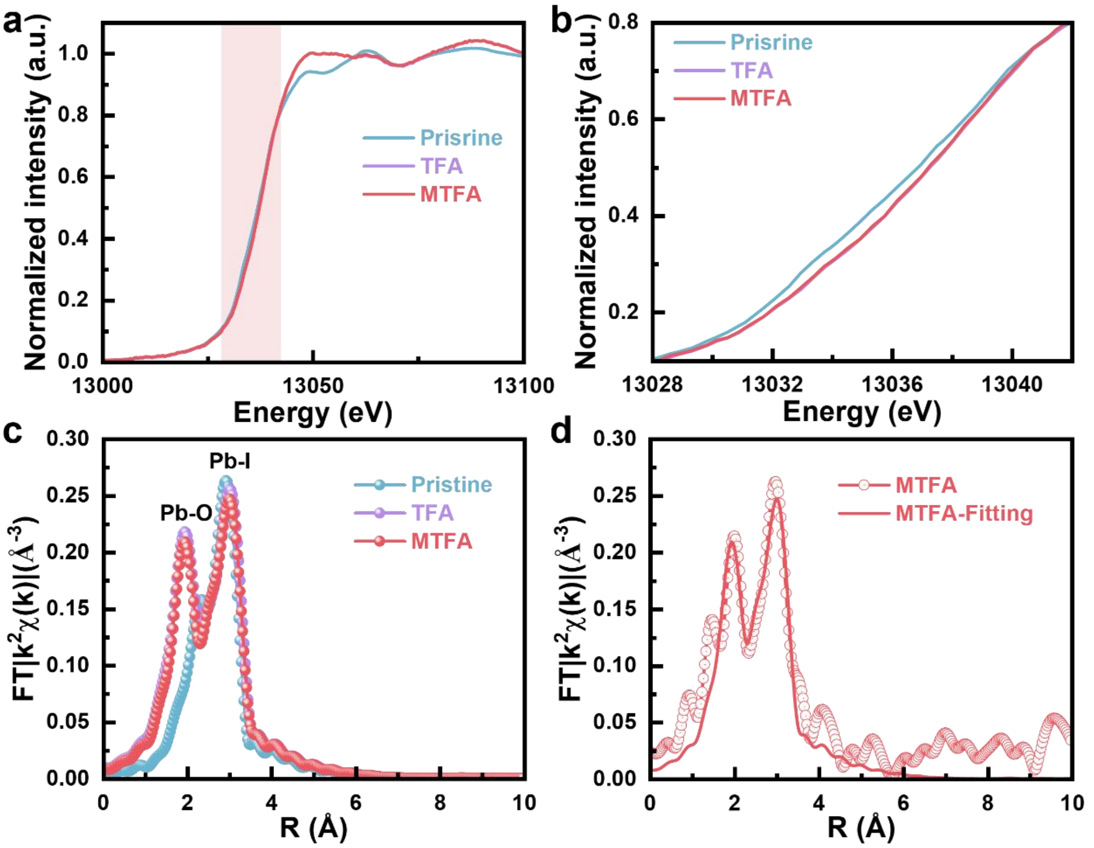

Supplementary Figure 24. XAS measurements of perovskite films with various corrosion inhibitors **(a)** Normalized Pb L_3-edged absorption coefficient, **(b)** The partial enlarged Normalized Pb L_3-edged absorption coefficient indexed to the energy range from 13028 eV to 13042 eV, **(c)** FT-EXAFS spectra in R-space for pristine, TFA and MTFA perovskite films and **(d)** EXAFS raw data and fitting spectrum of MTFA perovskite film.

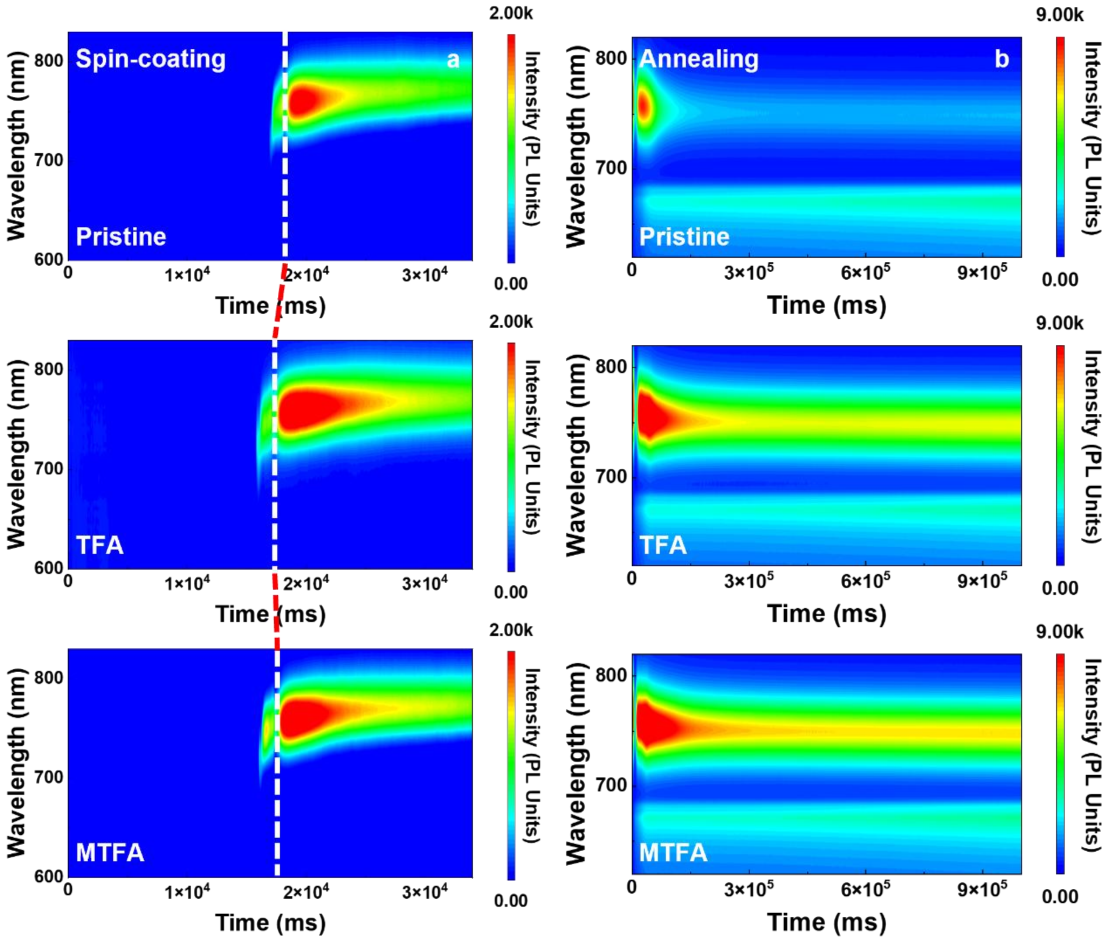

Supplementary Figure 25. *In situ* PL images of perovskite films during the **(a)** spin-coating and **(b)** thermal annealing process.

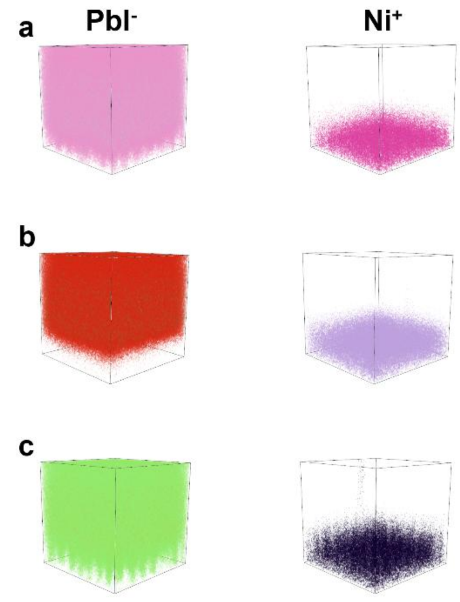

Supplementary Figure 26. The 3D spatial distribution of PbI^- and Ni^+ for the **(a)** pristine film, **(b)** TFA film and **(c)** MTFA film.

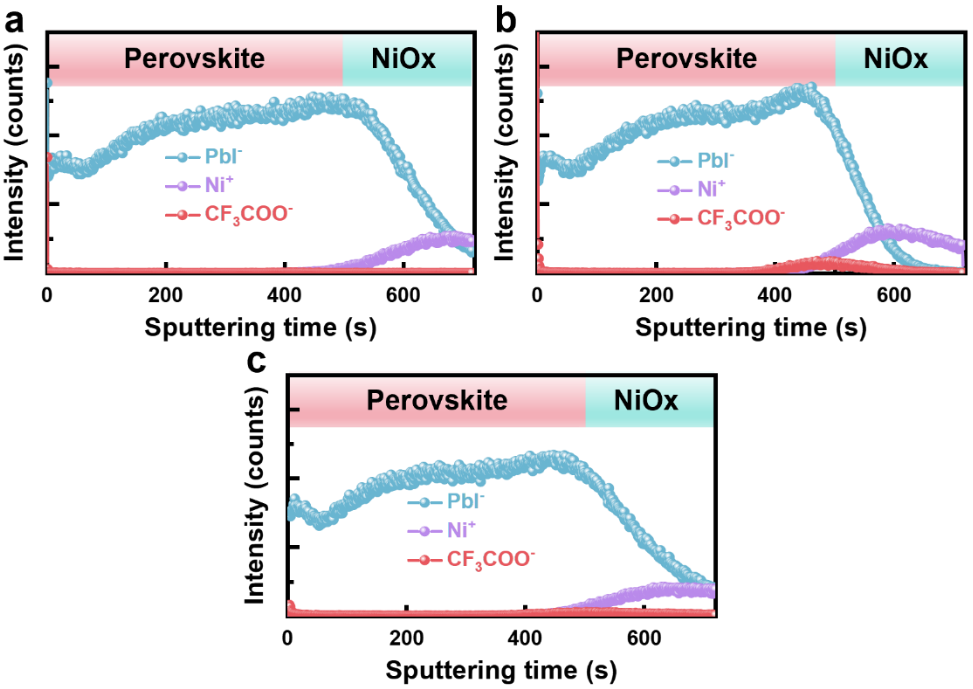

Supplementary Figure 27. ToF-SIMS depth-profile analysis of PbI^-, Ni^+ and CF3COO$^-$ species in the **(a)** pristine film, **(b)** TFA film and **(c)** MTFA film.

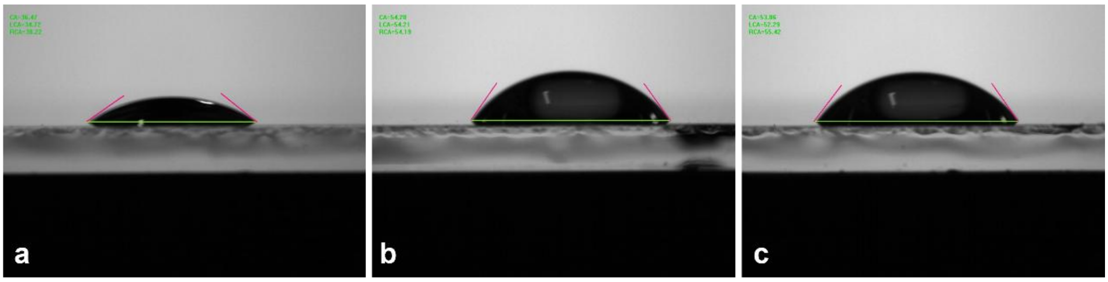

Supplementary Figure 28. Contact angle measurements of water on the **(a)** pristine film, **(b)** TFA film and **(c)** MTFA film.

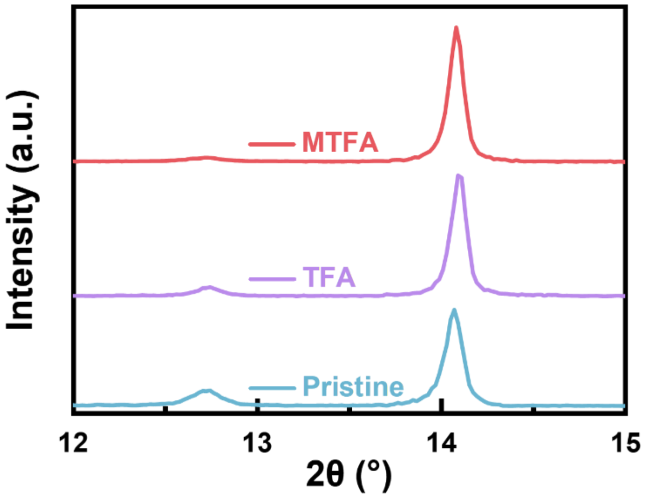

Supplementary Figure 29. Partially enlarged (100) diffraction peaks for perovskite films with various corrosion inhibitors.

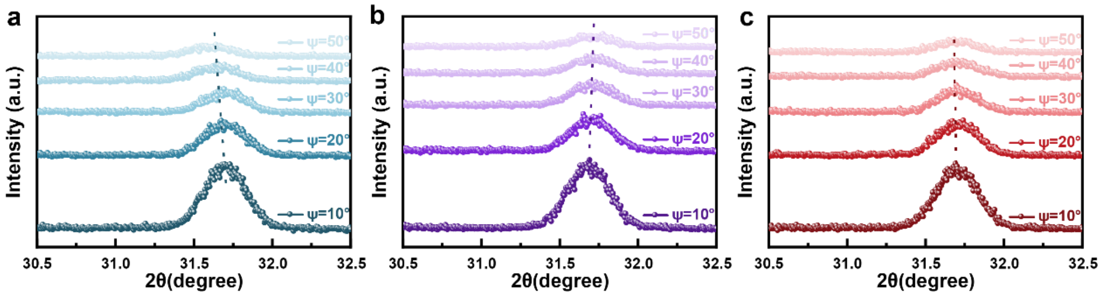

Supplementary Figure 30. GIXRD spectra at varied ψ angles of the **(a)** pristine film, **(b)** TFA film and **(c)** MTFA film. Where the (012) crystal facet positioned at a higher diffraction peak of 31.68° was deliberately chosen to validate the reliability of the structure information owing to its high multiplicity, which is typically performed to acquire elaborate grain information while effectively mitigating the crystal orientation effect on the linear relationship of 2θ-$\sin^2\psi$ in the GIXRD measurement.

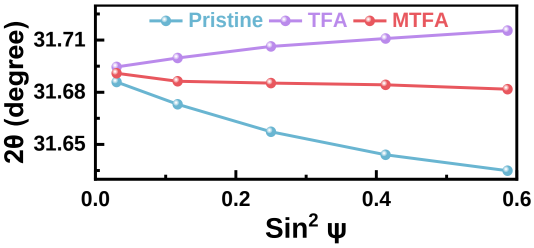

Supplementary Figure 31. Linear fit of 2θ-$\sin^2\psi$ curves for the different perovskite films.

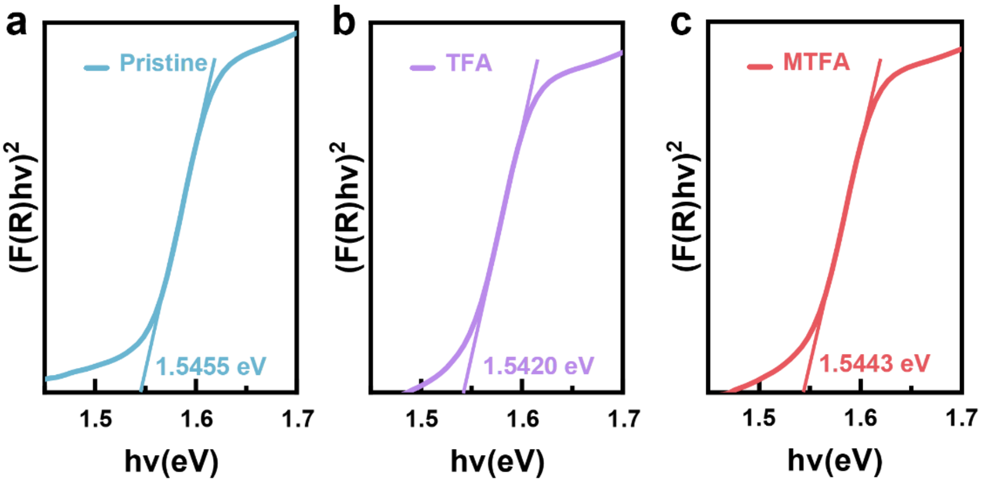

Supplementary Figure 32. Tauc plots extracted from the absorption spectral of **(a)** the pristine film, **(b)** the TFA film and **(c)** the MTFA film.

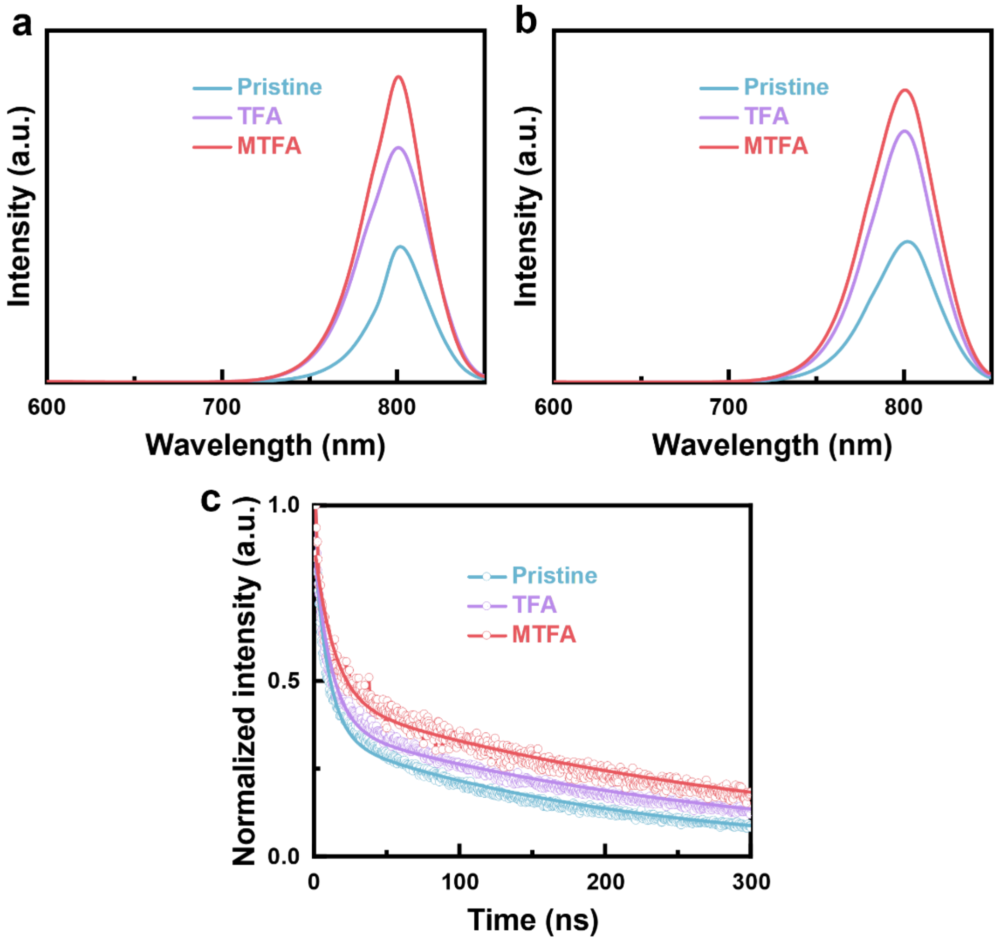

Supplementary Figure 33. (a) Front-side excitation and **(b)** back-side excitation PL spectra of perovskite films with different corrosion inhibitors, **(c)** TRPL spectra of perovskite films with different corrosion inhibitors.

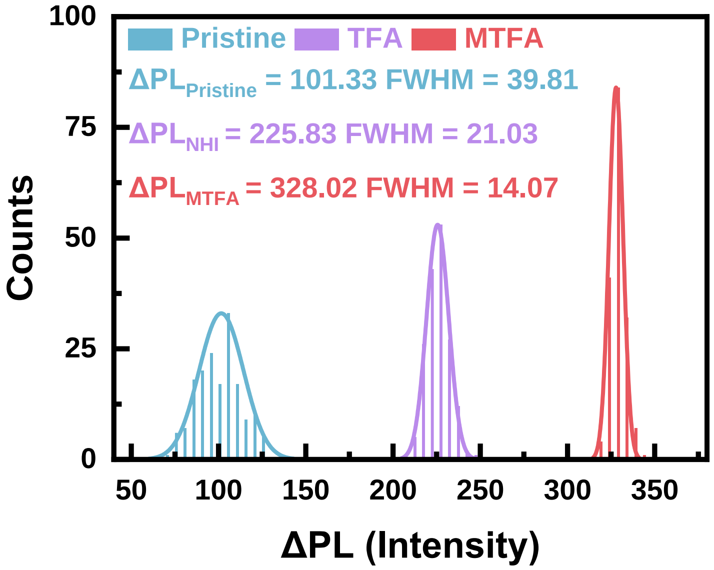

Supplementary Figure 34. Histogram of PL intensity extracted from the PL mapping of perovskite films with different corrosion inhibitors.

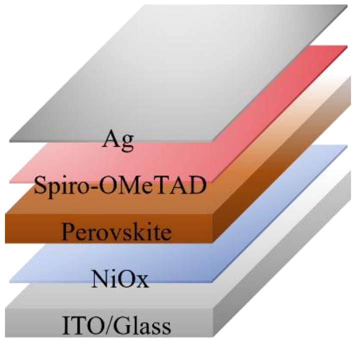

Supplementary Figure 35. Structure schematic diagram of the hole-only device for SCLC measurement.

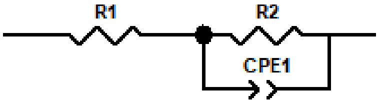

Supplementary Figure 36. Equivalent circuit model of the complete solar cells for EIS analysis.

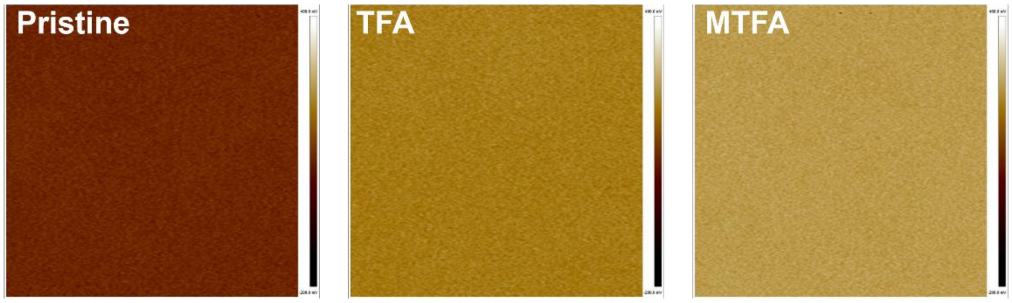

Supplementary Figure 37. KPFM images of perovskite films with different corrosion inhibitors.

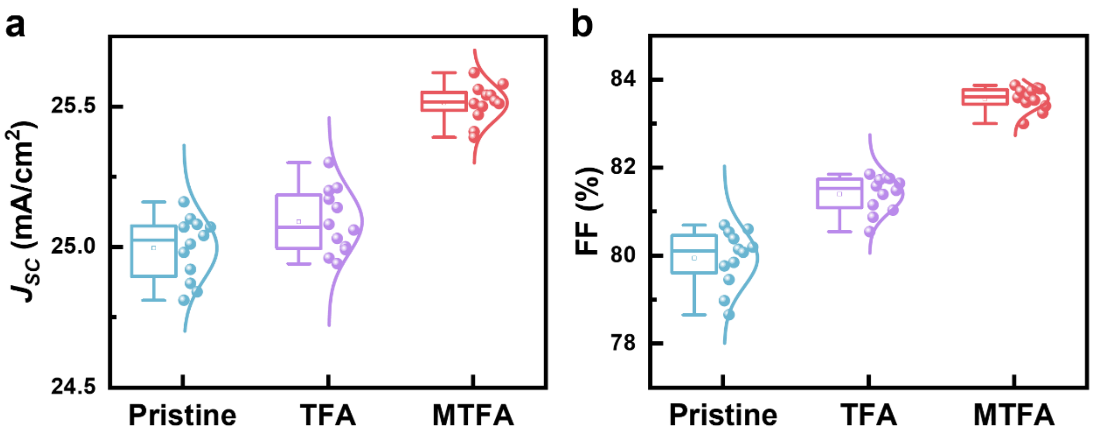

Supplementary Figure 38. (a) J_{SC} and **(b)** FF statistic distributions of 12 independent PSCs.

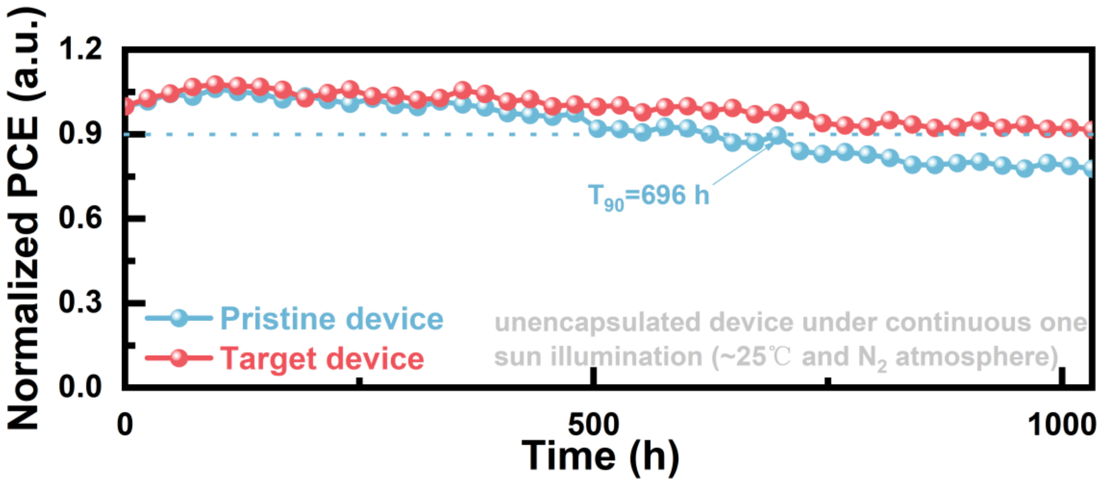

Supplementary Figure 39. MPP tracking test of unencapsulated PSCs under one sun irradiation in a ~25 ± 2 °C N_2 atmosphere.

Supplementary Table 1. Peak-area ratio of DoD=(A_{MF}+A_{DM})/(A_{MF}+A_{DM} +A_{MA}+A_{FA}) in the fresh and aged PPSs with and without TFA/MTFA. Where A_{MF}, A_{DM}, A_{MA} and A_{FA} are referred to the peak area of MFA^+, $DMFA^+$, MA^+, and FA in NMR spectra respectively.

	Pristine (%)	TFA (%)	10 μL/mL MTFA (%)	20 μL/mL MTFA (%)	30 μL/mL MTFA (%)
Fresh	6.12	2.32	2.79	2.57	2.30
10 days	7.39	2.98	2.56	2.41	2.22
20 days	8.92	3.71	2.40	2.33	2.17
30 days	9.05	4.39	2.83	2.53	2.34

Supplementary Table 2. The growth rate of DoD for PPSs with and without TFA/MTFA

	Pristine (%)	TFA (%)	10 μL/mL MTFA (%)	20 μL/mL MTFA (%)	30 μL/mL MTFA (%)
Fresh	-	-	-	-	-
10 days	20.75	28.45	-8.24	-6.22	-3.48
20 days	20.70	24.50	-6.25	-3.32	-2.25
30 days	14.57	18.33	17.92	8.58	7.83

Supplementary Table 3. The water content of polar aprotic solvents with different compositions before and after hygroscopic aging, as determined by Karl Fischer titration.

DMF: DMSO	1: 0 (%)	1: 1 (%)	0: 1 (%)
Fresh	0.03	0.02	0.02
30 days	1.11	0.95	1.09

Supplementary Table 4. Photoelectric performance parameters extracted from the *J-V* curves of PSCs assembled using freshly prepared PPSs.

Cells	J_{SC} (mA/cm^2)	V_{OC} (V)	FF (%)	PCE (%)
Pristine	25.27	1.20	82.47	24.95
TFA	25.45	1.22	83.55	25.91
MTFA-10 µL/mL	25.48	1.21	83.01	25.66
MTFA-20 µL/mL	25.59	1.22	83.81	26.24
MTFA-30 µL/mL	25.51	1.20	83.69	25.70

Supplementary Table 5. Photoelectric performance parameters extracted from the *J-V* curves of PSCs assembled using PPSs aged for 22 days.

Cells	J_{SC} (mA/cm^2)	V_{OC} (V)	FF (%)	PCE (%)
Pristine	25.16	1.19	80.69	24.15
TFA	25.30	1.20	81.85	24.79
MTFA-10 µL/mL	25.51	1.21	83.18	25.72
MTFA-20 µL/mL	25.62	1.23	83.87	26.35
MTFA-30 µL/mL	25.55	1.21	83.78	25.84

Supplementary Table 6. DoD value for the fresh and aged wide-band PPSs with and without MTFA.

	Pristine (%)	20 µL/mL MTFA (%)
Fresh	3.78	1.82
10 days	4.87	1.77
20 days	6.20	1.45
30 days	6.73	1.89

Supplementary Table 7. The *k* values of perovskite films extracted from their GIXRD spectral

	Pristine	TFA	MTFA
k	-0.092	0.037	-0.016

Supplementary Table 8. TRPL analysis result of the pristine and corrosion inhibitor samples.

Cells	τ_1 (ns)	τ_2 (ns)	τ_{avg} (ns)
Pristine	9.68	205.44	191.17
TFA	11.54	275.03	259.88
MTFA	13.56	315.01	301.90

Supplementary Table 9. Parameters derived from EIS measurements for the pristine and corrosion inhibitor samples.

	Pristine	TFA	MTFA
R_s (Ω)	38.78	36.81	30.85
R_{rec} (Ω)	2491	3127	3304

Supplementary Table 10. Photoelectric performance parameters extracted from the *J-V* curves of wide bandgap devices.

Cells	J_{SC} (mA/cm^2)	V_{OC} (V)	FF (%)	PCE (%)
Pristine	20.92	1.22	77.93	19.89
TFA	21.35	1.23	81.63	21.44
MTFA	21.58	1.26	82.90	22.49
