# Supplementary material for: Deprotonation suppressing via competitive proton transfer control for efficient perovskite solar cells
Source: Nat Commun. 2026 May 25;17:6829. doi: 10.1038/s41467-026-73620-0 (PMC13389051; doi:10.1038/s41467-026-73620-0)
Supplement: Supplementary file 2 — Reporting Summary [file 41467_2026_73620_MOESM2_ESM.pdf]

Corresponding author(s): NCOMMS-25-77085BC

Solar Cells Reporting Summary

Nature Portfolio wishes to improve the reproducibility of the work that we publish. This form is intended for publication with all accepted papers reporting the characterization of photovoltaic devices and provides structure for consistency and transparency in reporting. Some list items might not apply to an individual manuscript, but all fields must be completed for clarity.

For further information on Nature Research policies, including our data availability policy, see Authors & Referees.

Experimental design

Please check the following details are reported in the manuscript, and provide a brief description or explanation where applicable.

1. Dimensions

Area of the tested solar cells
- [x] Yes — The area of the tested solar cells were reported in the legend of Fig. 5a and Fig. 5b
- [ ] No — *Explain why this information is not reported/not relevant.*

Method used to determine the device area
- [x] Yes — The device area are determined by the shadow mask used in electron deposition
- [ ] No — *Explain why this information is not reported/not relevant.*

2. Current-voltage characterization

Current density-voltage (J-V) plots in both forward and backward direction
- [ ] Yes
- [x] No — This is not the research emphasis of this work

Voltage scan conditions
- [x] Yes — The scan range was set from 1.3 V to -0.2 V with a 0.02 V bisa step and a 20 ms daley time
- [ ] No — *Explain why this information is not reported/not relevant.*

Test environment
- [x] Yes — ambient air conditions??
- [ ] No — *Explain why this information is not reported/not relevant.*

Protocol for preconditioning of the device before its characterization
- [x] Yes — Operate the sunlight simulator under power for a stabilization period, typically 15 minutes
- [ ] No — *Explain why this information is not reported/not relevant.*

Stability of the J-V characteristic
- [x] Yes — All stability test conditions were deacripted in relevant Figure
- [ ] No — *Explain why this information is not reported/not relevant.*

3. Hysteresis or any other unusual behaviour

Description of the unusual behaviour observed during the characterization
- [ ] Yes — *Provide a description of hysteresis or any other unusual behaviour observed during the characterization.*
- [x] No — This is not the research emphasis of this work

Related experimental data
- [ ] Yes — *Provide a description of the related experimental data.*
- [x] No — This is not the reaearch emphasis of this work

4. Efficiency

External quantum efficiency (EQE) or incident photons to current efficiency (IPCE)
- [x] Yes — EQE spectra were characterized by a 150 W xenon lamp equipped with a monochromator
- [ ] No — *Explain why this information is not reported/not relevant.*

A comparison between the integrated response under the standard reference spectrum and the response measure under the simulator
- [x] Yes — In the description of Fig. 5e
- [ ] No — *Explain why this information is not reported/not relevant.*

For tandem solar cells, the bias illumination and bias voltage used for each subcell

☐ Yes
☒ No

Provide a description of the measurement conditions.

This work in un related to the tandem solar cells

5. Calibration

Light source and reference cell or sensor used for the characterization

☒ Yes
☐ No

light J-V curve were measured by a sunlight simulator (Oriel 92251A-1000) with a calibrated by a NREL-traceable KG5 filtered silicon reference cell

Explain why this information is not reported/not relevant.

Confirmation that the reference cell was calibrated and certified

☒ Yes
☐ No

NREL

Explain why this information is not reported/not relevant.

Calculation of spectral mismatch between the reference cell and the devices under test

☐ Yes
☒ No

Provide a value of the spectral mismatch and/or a description of how it has been taken into account in the measurements.

Spectral between the reference cell and the devices under test was self-corrected by our test system

6. Mask/aperture

Size of the mask/aperture used during testing

☒ Yes
☐ No

0.09 mm2

Explain why this information is not reported/not relevant.

Variation of the measured short-circuit current density with the mask/aperture area

☒ Yes
☐ No

basically consistent

Explain why this information is not reported/not relevant.

7. Performance certification

Identity of the independent certification laboratory that confirmed the photovoltaic performance

☐ Yes
☒ No

Identify the independent certification laboratory.

The efficiency of solar cell is not the lightspot of this work

A copy of any certificate(s)

☐ Yes
☒ No

Certificate copies should be provided in the Supplementary information. Please state the supplementary item number.

Solar cell in this work have not certified

8. Statistics

Number of solar cells tested

☒ Yes
☐ No

12

Explain why this information is not reported/not relevant.

Statistical analysis of the device performance

☒ Yes
☐ No

Fig. 5c, Fig. 5d and supplementary Figure 38

Explain why this information is not reported/not relevant.

9. Long-term stability analysis

Type of analysis, bias conditions and environmental conditions

☒ Yes
☐ No

All long-terms stability analysis were in accordance with the protocol of ISOS-L, ISOS-T or ISOS-D

Explain why this information is not reported/not relevant.
