# Supplementary material for: Deprotonation suppressing via competitive proton transfer control for efficient perovskite solar cells
Source: Nat Commun. 2026 May 25;17:6829. doi: 10.1038/s41467-026-73620-0 (PMC13389051; doi:10.1038/s41467-026-73620-0)
Supplement: Supplementary file 3 — Transparent Peer Review file [file 41467_2026_73620_MOESM3_ESM.pdf]

nature portfolio

Peer Review File

Deprotonation Suppressing via Competitive Proton Transfer Control for Efficient Perovskite Solar Cells

Corresponding Author: Professor Chunfu Zhang

This file contains all reviewer reports in order by version, followed by all author rebuttals in order by version.

Version 0:

Reviewer comments:

Reviewer #1

(Remarks to the Author)
The manuscript of "Deprotonation Suppressing via Competitive Proton Transfer Control for Efficient Perovskite Solar Cells" reported a corrosion inhibitor assessed strategy (methyl trifluoroacetate (MTFA)-assisted technique) to control competitive proton-transfer through its stepwise hydrolysis reaction and the gentle release of -COOH derived from trifluoroacetic acid (TFA) byproduct. Their findings indicate that MTFA stepwise hydrolysis play a key role in perovskite crystallization dynamic and halogen vacancies passivation. The optimized perovskite devices obtained a PCE of 26.24% and improved humidity stability. This work is meaningful and contributes to improving the perovskite solar cells humidity stability. However, there is no sufficient experimental data or analysis to support the research results. Thus, I think the paper is not suitable for publication in its current form, and major revision may be required:
1.This manuscript aims to "control competitive proton-transfer through its stepwise hydrolysis reaction to improve the humidity stability". The comparative NMR analysis of aged precursor solutions effectively demonstrates the efficacy of MTFA in suppressing deprotonation. To ensure the reproducibility and scientific rigor of this experiment, it is essential to specify the aging conditions in the manuscript, including duration, temperature, and atmospheric environment. Besides, undertaking a systematic study about how to control strategies for the hydrolysis kinetics of MTFA is important in manuscript.
2.The research of MTFA is currently limited to its solution-based anti-deprotonation effect. An important aspect for future consideration is whether the CH_3OH released by MTFA hydrolysis negatively impacts film quality and devices, for instance, by creating pinholes or defects as it escapes the film. The authors are suggested to explain the role of the film by MTFA.
3.In manuscript, the pristine film of O 1s peak shift shown in Figure 1(m) requires careful interpretation, as the oxygen signal can originate from multiple sources in a perovskite film
4.At the early stage of hydrolysis, both MTFA and its hydrolysis product, TFA, are present in the precursor solution. Owing to their distinct coordination chemistries and proton affinities, the authors should clarify whether they exhibit synergistic or competitive effects during the crystallization of the perovskite
5.The author investigates the influence of MTFA on the crystallization kinetics of the perovskite film, but the current discussion focuses solely on the nucleation stage. It is recommended that the authors provide additional analysis or discussion regarding the crystal growth rate.
6.Figure 4(e) is dark J-V curves rather than electrical impedance spectroscopy test. In addition, the dark J-V curves of MTFA-based device shown irregular points. The author should explain this phenomenon.
7.The authors should add X-ray absorption spectroscopy (XAS) to substantiate the proposed coordination environment
8.The steady-state PL spectra of TFA- and MTFA-treated films (Fig. S21) appear to contain more than one emission peak. Please provide a detailed discussion. In addition, please include back-side excitation PL to probe depth-dependent crystallization and the role of TFA/MTFA through the film thickness
9.Please discuss in detail the origin of strain changes in the perovskite films.

Reviewer #2

(Remarks to the Author)
The manuscript by Professor Zhang and colleagues presents an approach to enhance the stability of perovskite precursor solutions and the resulting perovskite solar cells through the incorporation of MTFA. While the work addresses an important challenge in perovskite device processing, the study lacks essential mechanistic clarity, quantitative evidence, and sufficient methodological detail to substantiate the claims. Several key experimental explanations, data analyses, and supporting

discussions are incomplete or insufficiently justified. Substantial revisions, additional experiments, and clearer presentation of results would be required to reach the level of rigor expected for publication in Nature Communications.
1. In Equation 1, water appears as a reactant. The authors must clarify the origin of this water molecule. If it originates from ambient moisture, then the precursor lifetime is highly dependent on humidity exposure. Additionally, the release kinetics of volatile CH3OH and the corrosion inhibitor TFA should be quantified or estimated to assess the precursor shelf life. Without this information, the stability mechanism remains speculative.
2. The effect of MTFA concentration on precursor shelf life and device performance is not sufficiently discussed. The authors should define the optimal concentration window and identify upper and lower limits that maintain stability and device efficiency. Further, it should be clarified whether this stabilization strategy is generalizable to different perovskite compositions (including bromide-rich wide-band-gap formulations) or is limited to the specific system studied.
3. The XPS peak assignments in Fig. 1k–m requires correction and clarification. The binding energy position of Pb 4f7/2 must appear at lower energy than Pb 4f5/2. Peak identification should be clearly labelled on the spectra, and the corresponding discussion revised accordingly.
4. Stability-testing conditions must be explicitly stated in the figures, including humidity level, temperature, illumination conditions, and whether the devices were encapsulated. Without these parameters, the stability data cannot be meaningfully interpreted.
5. All stability curves show an initial improvement in PCE before degradation. The authors should provide an explanation for this behaviour, supported by analysis or references.

6. Minor corrections and presentation issues:

(a) electrostatic surface potential (ESP) maps are presented in Figure 1d, whereas in text it is referred as Fig. 1c
(b) “Investigation on Perovskite Films Optoelectronic Characteristic” contains morphological analysis too hence the heading should be modified accordingly
(c) Figure 4d, in Nyquist plot, x- and y-axis values should be same making an square.
(d) Figure 4e, what are the values of voltage on X-axis?
(e) “As illustrated in Fig. 4d, the substantially reduced Jdark value of the MTFA device….” the dark J-V curves are illustrated in figure 4e. Similarly other figure numbering should be checked carefully.
(f) Figure 5a and b are giving the same information, one of them can be moved the supplementary file.
(g) Figure 5e, the EQE spectra are shown in percentage however the y-scale is only up to 1.

Reviewer #3

(Remarks to the Author)
The authors discussed the instability issue in FA-based perovskites caused by the irreversible deprotonation of the MA^+ cation inside the precursor solution. Thus, this deprotonation process limits the film quality and device performance. The study presents a MTFA–assisted strategy that avoids these limitations by slowly releasing –COOH groups via controlled hydrolysis, thereby reducing precursor deprotonation and enhancing the precursor shelf life. Additionally, authors reported that –COOCH3 and –COOH moieties modulate crystallization and passivate Pb2+ and halide vacancies, while CF3COO- species at the buried interface further improve carrier behaviour. This approach promises a more controlled and chemically stable route for FA-based perovskite fabrication.
Approaches aimed at inhibiting precursor ageing in FA-based perovskites are not new and should be acknowledged. Relevant recent reports such as https://www.nature.com/articles/s41467-025-63776-6, https://www.sciencedirect.com/science/article/pii/S0927024825001461. The authors should clarify how their approach fundamentally differs from prior works.
Can this MTFA-assisted strategy be extended to other FA-based compositions, including different bandgaps? Demonstrating broader applicability would significantly strengthen the study.
The table provided in the Supporting Information about DoD should be incorporated as a bar graph in Figure 1. As presented, the H-NMR spectra show minimal visible differences between TFA and MTFA, making quantitative comparison difficult.
The TRPL analysis is physically incorrect. The authors should revise this section and include excitation parameters (wavelength, fluence, repetition rate) directly in the figure caption. For guidance, see the reference papers below: https://advanced.onlinelibrary.wiley.com/doi/full/10.1002/aenm.202403279, https://www.nature.com/articles/ncomms13941
In Figure 2 discussion part, it is stated that the subsequent investigations are based on aging precursors (how long is missing). If MTFA slows precursor ageing, the manuscript should also include device performance and film characterization from fresh and aged precursor solutions. As written, the study resembles a comparison of two additives rather than a precursor-stability analysis. The long-term stability of devices fabricated from fresh and aged solutions is also missing.
Figure 5a and b are repetitive?
The mapping between pH values and sample conditions is unclear; please specify which reading corresponds to which solution.
In Figure 2, panels (a) and (b) mentioned in the caption are missing from the figure and must be corrected.
In general, Captions should be significantly more descriptive. For instance, Figure 5h should be labelled following ISOS-T conventions (including temperature, encapsulation, storage, and measurement conditions). T90 reference lines should also be included.

Reviewer #4

(Remarks to the Author)
The manuscript presents a well-motivated and timely study on stabilizing FA-based perovskite precursor solutions by introducing methyl trifluoroacetate (MTFA). The authors provide a detailed mechanistic analysis of deprotonation pathways and convincingly show that MTFA undergoes gradual hydrolysis to deliver controlled TFA generation while suppressing competitive proton transfer and byproduct formation. This precursor-solution stabilization concept is meaningful and has clear relevance to reproducible, high-quality perovskite film fabrication. Overall, this manuscript explores an important challenge in perovskite solar cell fabrication and proposes a promising solution-stabilization strategy. With clearer reporting of precursor aging conditions and the inclusion of time-dependent film and device data that directly reflect the anti-aging effect, the study will be significantly strengthened and provide compelling evidence for the advantages of MTFA in practical processing. Therefore, several clarifications and additions are needed before publication.

Although the manuscript emphasizes the critical importance of precursor aging, the actual aging conditions of the solutions used to fabricate the perovskite films and devices are not explicitly stated. It is unclear how long the pristine, TFA-treated, and MTFA-treated solutions were stored prior to use, or whether they were compared under equivalent storage durations. Since the central significance of MTFA lies in its ability to maintain stability during storage, presenting film and device characteristics as a function of controlled aging time for all three precursor solutions would strengthen the manuscript considerably. Such data would clearly demonstrate the practical advantage of MTFA and would allow the reader to assess how differences in deprotonation and byproduct formation directly influence film morphology, crystallization dynamics, defect states, and photovoltaic performance.

The paper also offers extensive analysis of solution-phase chemistry, including NMR quantification, HI detection, and simulation results, but the connection between these solution-level changes and the subsequent film-formation processes could be made more explicit. Clarifying how the extent of deprotonation or hydrolysis in the solution translates into the nucleation behavior, growth kinetics, and defect landscape of the resulting films would provide deeper mechanistic insight and better align with the overall message of the work.

In addition, the interpretation of the increased built-in potential would benefit from some form of direct energetic evidence, such as UPS or KPFM, to support the discussion of interfacial band bending.

The X-axis label in Figure S17 should be changed.

Version 1:

Reviewer comments:

Reviewer #1

(Remarks to the Author)
The authors have addressed some of the raised concerns in their revision. However, the science of the data is still concern to me. Hence, this manuscript is recommended for publication after minor revision, and the detailed comments are as follows:
1. The authors should add X-ray absorption spectroscopy (XAS) to substantiate the proposed coordination environment.

Reviewer #2

(Remarks to the Author)
The authors have responded comprehensively and convincingly to all reviewer comments. The revised manuscript shows a substantial improvement in scientific rigor, clarity, and presentation compared to the original submission. The work presents a meaningful conceptual advance in stabilizing perovskite precursor solutions and offers clear relevance for scalable and reproducible perovskite solar cell fabrication. I believe the manuscript is now suitable for publication in Nature Communications.

Reviewer #3

(Remarks to the Author)
The authors have improved the manuscript substantially by performing additional experiments and incorporating new discussions in both the main text and Supporting Information.

As a note regarding the TRPL analysis:

Under identical excitation and measurement conditions, TRPL decays can indeed be compared on a relative basis. However, fitting the decay curves with a bi-exponential function does not, by itself, provide direct physical insight into the underlying photophysical processes.

In particular, assigning the fast component (τ_1) to non-radiative recombination and the slow component (τ_2) to bulk

recombination is not physically justified without additional supporting evidence. Although many published papers follow a similar bi-exponential fitting approach and interpret τ_1 and τ_2 in this manner, the prevalence of this practice does not necessarily validate the physical interpretation. Therefore, the following discussion is not rigorously supported:

“Herein, A_1 and A_2 represent the respective amplitudes, while τ_1 and τ_2 denote the PL decay times. Since the rapidly decay component (τ_1) is assigned to the non-radiative recombination resulting from charge-trapping defects as well as the quenching of charge carriers at the interfaces. Meanwhile, the slower decay component (τ_2) is associated with the recombination processes occurring within the film bulk…”

Such mechanistic attribution goes beyond what can be concluded from biexponential fitting alone.

Instead, I recommend restricting the interpretation to a comparative analysis based on the average lifetime under identical measurement conditions, without assigning specific recombination channels to individual decay constants. This would make the TRPL discussion physically sound while still supporting the comparative conclusions of the study.

Reviewer #4

(Remarks to the Author)
The authors have carefully and comprehensively addressed all of the reviewer’s concerns in the revised manuscript. In particular, the explicit clarification of precursor aging conditions, the inclusion of time-dependent film and device data, and the additional energetic characterization significantly strengthen the scientific rigor and transparency of the study. The revised version now presents a clear and convincing link between solution stability, crystallization behavior, and device performance evolution. In my opinion, the manuscript has been substantially improved and now provides compelling evidence supporting the proposed MTFA-assisted precursor stabilization strategy. I therefore recommend publication of the revised manuscript in its present form.

Version 2:

Reviewer comments:

Reviewer #1

(Remarks to the Author)
The authors have addressed all the raised concerns in their revision. I therefore recommend that this manuscript be published in Nature Communication.

Open Access This Peer Review File is licensed under a Creative Commons Attribution 4.0 International License, which permits use, sharing, adaptation, distribution and reproduction in any medium or format, as long as you give appropriate credit to the original author(s) and the source, provide a link to the Creative Commons license, and indicate if changes were made.
In cases where reviewers are anonymous, credit should be given to 'Anonymous Referee' and the source.
The images or other third party material in this Peer Review File are included in the article’s Creative Commons license,

unless indicated otherwise in a credit line to the material. If material is not included in the article's Creative Commons license and your intended use is not permitted by statutory regulation or exceeds the permitted use, you will need to obtain permission directly from the copyright holder.
To view a copy of this license, visit https://creativecommons.org/licenses/by/4.0/

Reply to the Reviewer's Comments

Dear Editor,

Thank you very for your Letter dated 1th Dec. 2025 to inform us to revise our manuscript entitled "Deprotonation Suppressing via Competitive Proton Transfer Control for Efficient Perovskite Solar Cells". We thank you and the reviewers for constructive comments and suggestions on our manuscript as well as on our work. According to the comments, we have done our best to make revisions on our manuscripts. All the modifications are highlighted by ***yellow background*** in the revised manuscript for clarity.

Our point-by-point responses to the reviewers' Comments/Questions are provided as followings.

We hope this improved version has met the requirements for publication in Nature Communications.

Many thanks again to you and the reviewers.

Correspondence should be addressed to:

Prof. Chunfu Zhang, email address: cfzhang@xidian.edu.cn.

Sincerely yours,

Chunfu Zhang

School of Microelectronics, Xidian University

Xi'an 710071, P. R. China.

Reviewer #1: *The manuscript of "Deprotonation Suppressing via Competitive Proton Transfer Control for Efficient Perovskite Solar Cells" reported a corrosion inhibitor assessed strategy (methyl trifluoroacetate (MTFA)-assisted technique) to control competitive proton-transfer through its stepwise hydrolysis reaction and the gentle release of -COOH derived from trifluoroacetic acid (TFA) byproduct. Their findings indicate that MTFA stepwise hydrolysis play a key role in perovskite crystallization dynamic and halogen vacancies passivation. The optimized perovskite devices obtained a PCE of 26.24% and improved humidity stability. This work is meaningful and contributes to improving the perovskite solar cells humidity stability. However, there is no sufficient experimental data or analysis to support the research results. Thus, I think the paper is not suitable for publication in its current form, and major revision may be required:*

Response: We sincerely appreciate your recognition of the significance of our manuscript in improving the humidity stability of PSCs, as well as your constructive feedback emphasizing the need for more robust experimental evidence to further substantiate our claims. We fully acknowledge that rigorous experimental validation is paramount, and have therefore conducted a comprehensive revision of the manuscript. This includes performing additional controlled experiments, incorporating more in-depth analytical characterization and reinforcing the logical coherence across all sections. Our point-by-point responses are as follows:

Question 1: *This manuscript aims to "control competitive proton-transfer through its stepwise hydrolysis reaction to improve the humidity stability". The comparative NMR analysis of aged precursor solutions effectively demonstrates the efficacy of MTFA in suppressing deprotonation. To ensure the reproducibility and scientific rigor of this experiment, it is essential to specify the aging conditions in the manuscript, including duration, temperature, and atmospheric environment. Besides, undertaking a systematic study about how to control strategies for the hydrolysis kinetics of MTFA is important in manuscript.*

Response: We sincerely thank you for the positive feedback on our NMR analysis and for these crucial suggestions to enhance the experimental reproducibility and

mechanistic depth of our work. We have addressed both points thoroughly in the revised manuscript as follow:

Aging conditions of PPSs:

The evolution of chemical species in MTFA-assisted PPSs was initially elucidated in this study through 30 days of continuous ^{1}H nuclear magnetic resonance (NMR) spectroscopy detecting, with data extraction interval of 10 days and all samples were stored in the atmospheric environment (~25 °C and ~35% RH).

Hydrolysis kinetics of MTFA:

Ultimately, the superior deprotonation inhibition state is acquired by the MTFA-doped PPS sample as aging time extended capitalizing on its gradually released corrosion inhibitor of TFA via hydrolysis reaction in chemical formula (5). Therein, the trace amounts of water essential for MTFA hydrolysis are predominantly introduced through the highly hygroscopic nature of polar aprotic solvents (Dimethyl Sulfoxide (DMSO) and N,N-dimethylformamide (DMF)) as depicted in **Table S3**, both the DMF and DMSO declared inelegant hygroscopicity after 10 days atmospheric storage (~25 °C and ~35% RH).

$$CF_3COOCH_3 + H_2O \rightarrow CF_3COOH + CH_3OH \tag{5}$$

This could facilitate the suppressed deprotonation of PSS while simultaneously preventing the undesirable competitive proton-transfer induced by superfluous TFA$^-$, as evidenced by the progressively increasing CH_3OH signal over aging duration in **Fig. S10**, the correspondingly mirrored decreasing MTFA signals in **Fig. S11**, and the reducing PH values in **Fig. S8b**. Notably, an upward reversal in DoD values for all MTFA-doped PPS samples were occurred between the 20th and 30th days derived from the approaching depletion of MTFA, as confirmed by the partial enlarged ^{1}H NMR spectra in **Fig. S11**. Overall, these finding determined that despite the TFA initially achieved a conspicuous deprotonation inhibition, the competitive proton-transfer from HI to superfluous TFA$^-$ remains a critical factor in prolonging the duration and violence of deprotonation process, thereby resulting in a relatively higher DoD in relevant aging PPS. Whereas, replacing TFA with MTFA has effectively mitigated this unacceptable prolongation of deprotonation by enabling the gradual released TFA, which avoiding

the unpopular competitive proton-transfer induced by superfluous TFA^- as well as extending the deprotonation suppression period of MTFA-doped PPS to over 20 days.

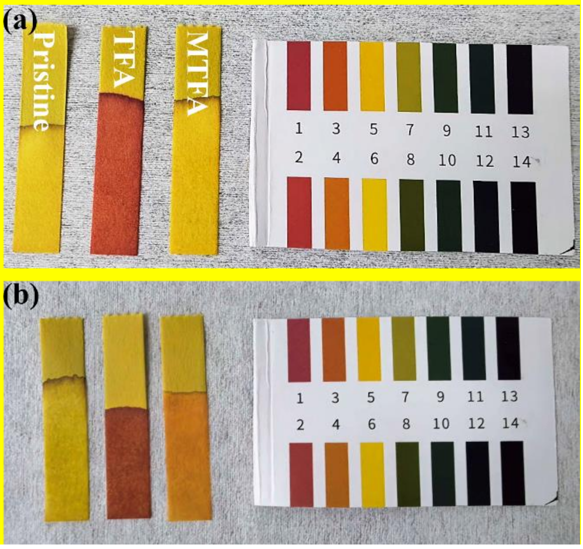

Figure S8. The pH tests of the **(a)** fresh and **(b)** aged pristine/TFA/MTFA-doped PPS, with both TFA and MTFA doped at a concentration of 20 μL/mL.

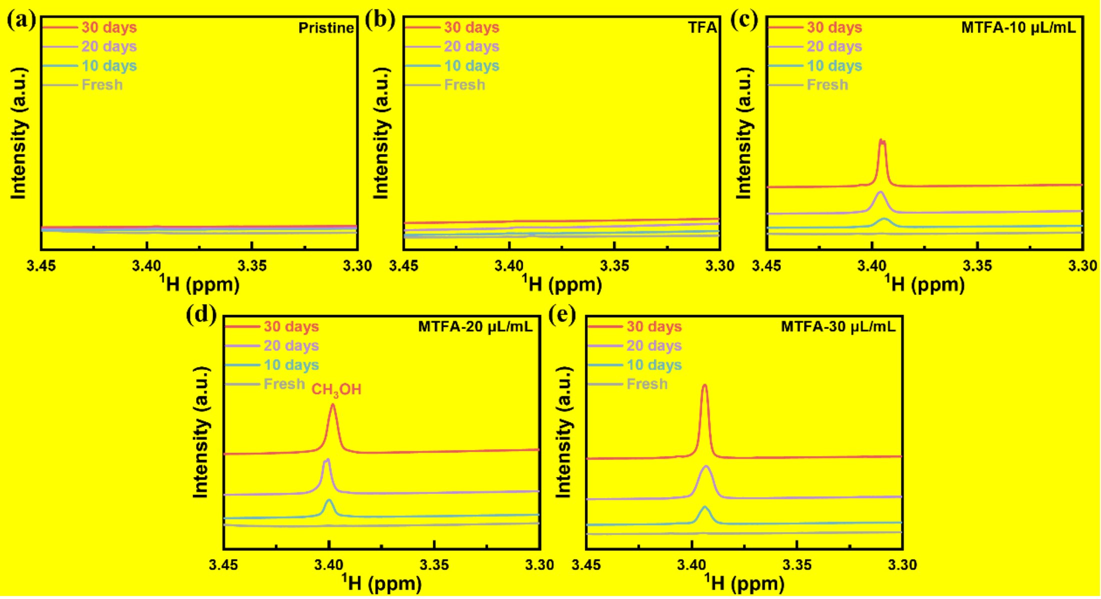

Figure S10. The partial enlarged ^{1}H NMR spectra indexed to the CH_3OH of the pristine/TFA/MTFA-doped PPS (DMSO-d_6).

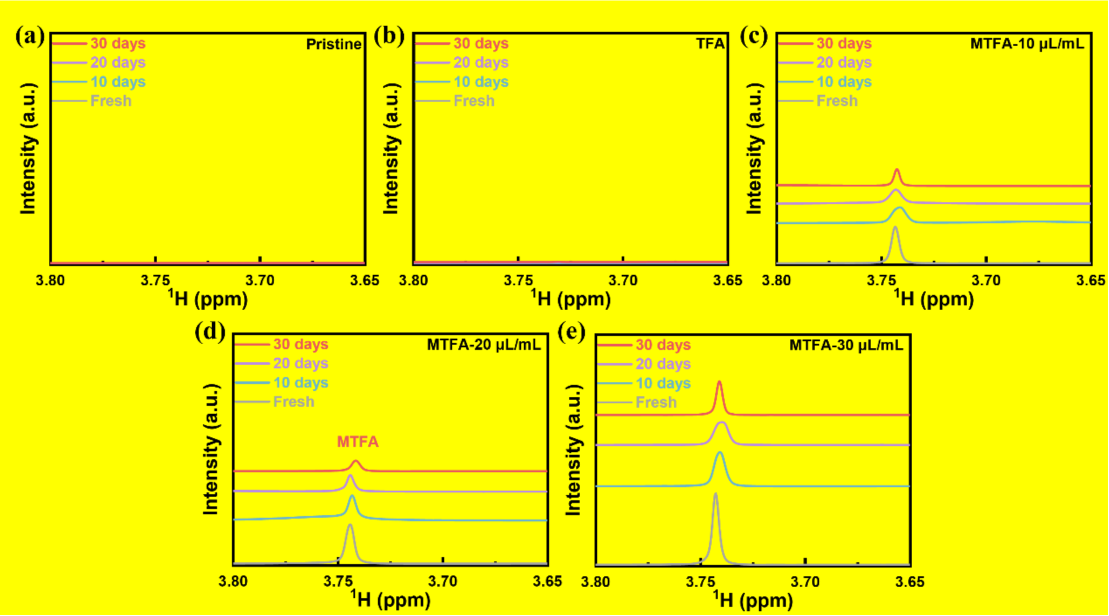

Figure S11. The partial enlarged ^{1}H NMR spectra indexed to the MTFA signal of the pristine/TFA/MTFA-doped PPS (DMSO-d$_6$).

Table S3. The water content of polar aprotic solvents with different compositions before and after hygroscopic aging, as determined by Karl Fischer titration.

DMF: DMSO	1: 0 (%)	1: 1 (%)	0: 1 (%)
Fresh	0.03	0.02	0.02
30 days	1.11	0.95	1.09

Question 2: *The research of MTFA is currently limited to its solution-based anti-deprotonation effect. An important aspect for future consideration is whether the CH_3OH released by MTFA hydrolysis negatively impacts film quality and devices, for instance, by creating pinholes or defects as it escapes the film. The authors are suggested to explain the role of the film by MTFA.*

Response: We sincerely appreciate your thoughtful suggestion regarding the potential impact of released CH_3OH on film formation and fully agree that the impacts of hydrolysis byproducts are crucial for a complete mechanistic understanding. Our comprehensive data indicated that the release of CH_3OH has no negative impact for the following reasons:

(1) Optimized film quality in MTFA assisted samples: Our SEM and AFM images provided direct evidence that the MTFA sample exhibits a significantly more compact

and pinhole-free surface compared to the pristine sample, along with a marked reduction in grain boundary cracks and a lower RMS roughness, revealing the escaped CH_3OH has no intention of deteriorate the film quality.

(2) Early and rapid volatilization: The relatively lower boiling point of CH_3OH (64.7 °C) compared to the DMF (153 °C) and DMSO (189 °C) implied that the hydrolysis byproduct CH_3OH was volatilized rapidly during the thermal annealing process (100 °C, 30 min), concurrently with the evaporation of the primary solvents (DMF/DMSO). This feature minimizes the potential for disruptive bubbling or pinhole formation caused by the release of CH_3OH from the wet perovskite film.

Question 3: *In manuscript, the pristine film of O 1s peak shift shown in Figure 1(m) requires careful interpretation, as the oxygen signal can originate from multiple sources in a perovskite film.*

Response: We sincerely thank the reviewer for this critical and insightful comment. We fully acknowledge the reviewer's concern and agree that a definitive assignment of the O 1s signal in perovskite films is challenging due to its potential composite origin. However, in this manuscript, the blueward shifted diffraction peaks of the O 1s is chemically consisted with the opposing shifted diffraction peak of Pb 4f and I 3d core levels. This correlated behavior, combining with the direct evidence extracted from FTIR and DFT results, supported the interpretation that the shifted O 1s peak is primarily induced by the interaction between C=O groups of additives and the Pb-I lattice, rather than to other oxygen-containing species within the perovskite film. Accordingly, we have integrated this analytical discussion into our revised manuscript as follows:

In contract, diffraction peaks of the O 1s in **Fig. 1m** were red shifted toward lower binding energy upon the mixing of TFA and MTFA, which is chemically correlated with the opposing shifts observed for the Pb 4f and I 3d core levels. Especially, combined with analysis from DFT simulations and FTIR spectroscopy, this red-ward shift is specifically attributed to the C=O group of corrosion inhibitors upon coordinating with the Pb-I lattice, rather than potential ambient adsorbates or impurities in perovskite films. This provided compelling evidence for the formation of $C{=}O{\rightarrow}Pb^{2+}$ coordination

motifs between the inhibitors and Pb-I lattice, underscoring the superior chemical engagement exhibited by the TFA-based corrosion inhibitor.

Question 4: *At the early stage of hydrolysis, both MTFA and its hydrolysis product, TFA, are present in the precursor solution. Owing to their distinct coordination chemistries and proton affinities, the authors should clarify whether they exhibit synergistic or competitive effects during the crystallization of the perovskite.*

Response: We are grateful to the reviewer for highlighting this crucial point regarding the dynamic interplay between MTFA and its hydrolysis product (TFA) during the crystallization of the perovskite, which was already concerned at the initial stage of this research. Notably, FTIR spectra of the MTFA/PbI_2 mixture powder directly declared that both MTFA and its hydrolysis product TFA exhibited synergistic effects during the crystallization of the perovskite through concurrently coordinating with the Pb-I framework, where two distinct and well-resolved vibrational peaks (1679.35 and 1634.96 cm^{-1}) was observed. Thereinto, vibrational peak at 1679.35 cm^{-1} was assigned to the C=O→Pb^{2+} coordination from the parent MTFA, while the vibrational peaks at 1634.96 cm^{-1} was indexed to the C=O→Pb^{2+} coordination from its hydrolysis product TFA. Significantly, both vibrational peaks were blue shifted from the free C=O peak at 1712.37 cm^{-1}, indicting simultaneous coordination affinity between MTFA/TFA and the Pb-I framework as well as synergistic effects during the crystallization of the perovskite. Accordingly, we have revised our manuscript to clearly emphasize this key aspect as follows:

Notably, two distinct vibrational peaks (1679.35 and 1634.96 cm^{-1}) emerged prominently in the MTFA + PbI_2 spectrum, which were assigned with C=O→Pb^{2+} coordination modes originating from MTFA and TFA respectively. This provides direct evidence for the coexistence of both MTFA and its hydrolysis product TFA, as well as their simultaneous coordination to the Pb-I framework, laterally confirming the occurrence of its unique hydrolysis reaction.

Question 5: *The author investigates the influence of MTFA on the crystallization kinetics of the perovskite film, but the current discussion focuses solely on the nucleation stage. It is recommended that the authors provide additional analysis or*

discussion regarding the crystal growth rate.

Response: We sincerely thank your valuable suggestion and fully agree that a comprehensive analysis should encompass both nucleation and growth stages. Accordingly, we have revised our manuscript and emphasized the virtue of MTFA and TFA regarding the crystal growth rate regulation particularly as follows.

Furthermore, *in-situ* UV-Vis and *in-situ* PL spectrograms during the annealing process were further proposed after transferring the precast films to a hotplate, with the aim of gaining insights into the crystal growth behaviors of perovskite films containing various corrosion inhibitors. As depicted in **Fig. 2b**, two sequential absorption phase evolutions were acquired across all samples throughout the whole monitoring period: an initial absorption enhancement stage and the subsequent plateau stage. Relevant absorbance evolutions extracted at the peak wavelength of 500 nm were illustrated in **Fig. 2d**, thereby comprehensively inquiring into the crystal growth behaviors of perovskite films during the annealing process. During the ascending stage, both the TFA and MTFA samples demonstrated the highest initial absorbance coupled with a skyrocket enhanced in absorbance compared to the pristine sample, with the TFA sample showing the most pronounced effects. This compelling observation aligns remarkably well with the nucleation density ranking presented in **Fig. 2a**, indicating a potential functionality of TFA/MTFA in modulating the crystal growth of perovskite films owe to the enhanced interaction between the TFA/MTFA and Pb-I lattice as well as the resulting drastic nucleation aggregation. Subsequently, all samples were transitioned into a plateau stage as the annealing duration extended, while the champion absorption intensity in this region is belonged to the MTFA sample, thereby underscoring its superior crystalline characteristic induced by the synergistic action of suitable crystallization dynamic regulation and balanced stoichiometric ratio within PPS derived from suppressing cation deprotonation. A similar tendency was also detected in the *in-situ* PL spectrograms as shown in **Fig. S24b** and **Fig. 2f**, substantiating the credibility of the aforementioned analysis. Synthesizing these *in-situ* images, it is concluded that the crystallization behavior regulation of both the TFA and MTFA samples are not only

function in the adjusting initial absorbance, but also the crystal growth rate of relevant perovskite films.

Question 6: *Figure 4(e) is dark J-V curves rather than electrical impedance spectroscopy test. In addition, the dark J-V curves of MTFA-based device shown irregular points. The author should explain this phenomenon.*

Response: We sincerely thank your insightful question concerning the incorrect figure labeling and for bringing to our attention the irregular points of the dark J-V curves of MTFA-based device. We have already corrected the figure labeling error in the revised manuscript: the dark J-V curves are now presented in **Fig. 4d** and the Nyquist plots (EIS) are correctly shown in **Fig. 4e**. Additionally, the irregular points in dark J-V curves of MTFA-based device perhaps triggered by transient measurement noise and We have re-measured all dark J-V characteristics under optimized conditions, and the new curve (revised **Fig. 4e**) of MTFA device was smooth and exhibiting the lowest dark current, robustly confirming its superior suppression of trap-assisted recombination.

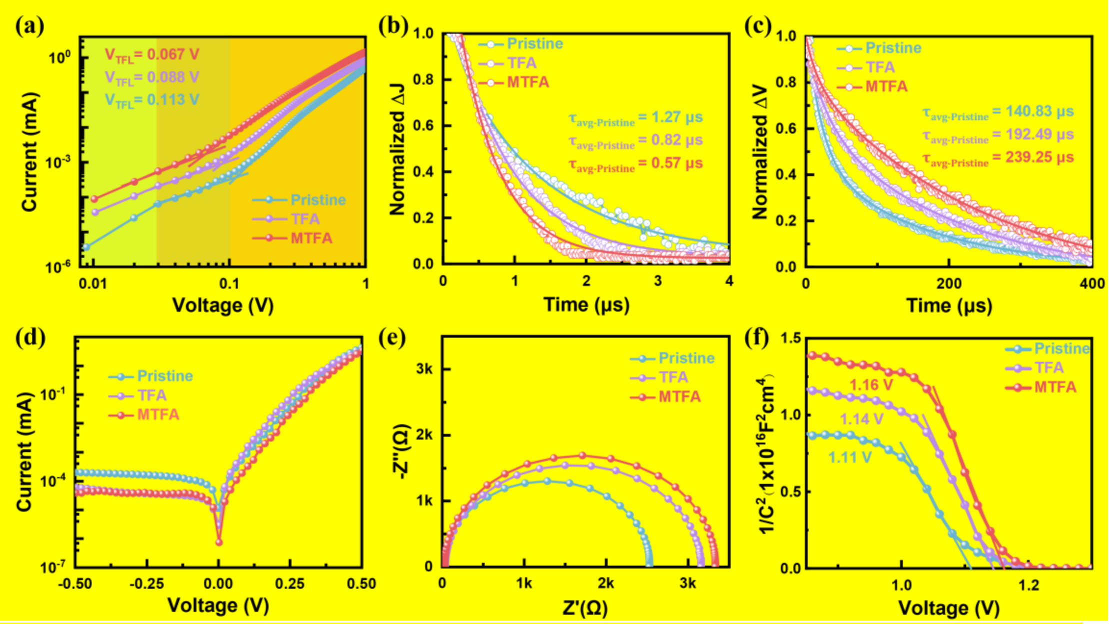

Figure 4. (a) SCLC analyses, **(b)** TPC curves, **(c)** TPV curves, **(d)** dark *J-V* curves, **(e)** Nyquist plots and **(f)** M-S plots of final PSCs.

Question 7: *The authors should add X-ray absorption spectroscopy (XAS) to substantiate the proposed coordination environment.*

Response: We sincerely thank you for your constructive comments and for the opportunity to improve our manuscript. We appreciate your suggestion regarding the

addition of XAS (X-ray Absorption Spectroscopy) data for further probe the coordination environment within corrosion inhibitors containing perovskite films.

However, limited by our current facility access constraints, we are unable to perform this specific experiment at this stage. Importantly, we would like to emphasize that in our initial manuscript, the coordination interaction between the carbonyl groups of TFA/MTFA and the Pb-I framework was robustly supported by multiple complementary characterization techniques (FTIR, XPS and DFT). Concretely, our DFT calculations quantitatively confirmed the E_{ads} between TFA/MTFA and either the Pb-I framework or FA^+, declaring the superior E_{ads} regarding TFA/MTFA interaction with the Pb-I framework than that with FA^+. This result was further confirmed by the significant red shifted C=O stretching vibration in the TFA/MTFA + PbI_2 FTIR spectra, along with correlated shifts in core level binding energies, where both the Pb 4f and the I 3d were shifted to higher binding energy while the O 1s was shifted to lower binding energy.

We believe that the combination of these techniques has provided a robust and consistent understanding of the proposed coordination environment and has sufficiently supported the claims made in the manuscript. We also hope that the above analysis adequately addresses your concerns.

Question 8: *The steady-state PL spectra of TFA- and MTFA-treated films (Fig. S21) appear to contain more than one emission peak. Please provide a detailed discussion. In addition, please include back-side excitation PL to probe depth-dependent crystallization and the role of TFA/MTFA through the film thickness*

Response: We thank you for your excellent and insightful suggestions regarding the PL analysis aimed at elevating the overall quality of our work. As you pointed out, the shoulder peaks in the steady-state PL spectra are present across all samples in our initial manuscript, probably resulting from the light/moisture-induced phase segregation in perovskite films as demonstrated in previous work, which is caused by our inadequate film encapsulation technology during the outsourced PL testing. Herein, steady-state PL excited from both the front side (perovskite film) and back side (glass) were retested in our revised manuscript and the shoulder peaks were eliminated. Corresponding

updated analysis is as follows:

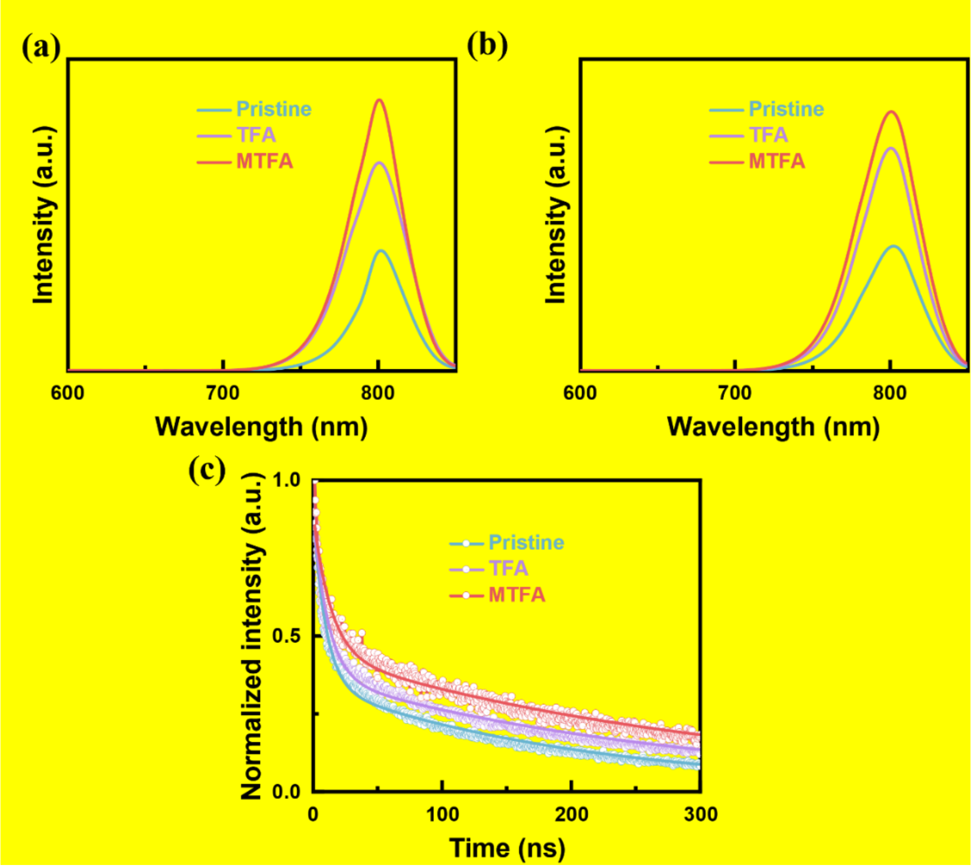

Figure S32. (a) Front-side excitation and **(b)** back-side excitation PL spectra of perovskite films with different corrosion inhibitors, **(c)** TRPL spectra of perovskite films with different corrosion inhibitors.

Subsequently, steady-state PL excited from the front side (perovskite film) and back side (glass), PL mapping images and time-resolved photoluminescence (TRPL) decay profiles were proposed successively to evaluate the carrier dynamic of the three samples. As illustrated in **Fig. S32a-b**, the incorporation of corrosion inhibitors directly caused dramatically enhanced emission peaks in the PL spectra acquired from both the front-side and back-side of relevant samples compared to the pristine one, declaring their optimized crystalline characteristic and reduced carrier recombination centers. Notably, a more pronounced peak intensity gap between the MTFA and TFA samples was emphasized in the front-side excited PL spectra, indicating superior suppression of non-radiative recombination at the buried interface of the TFA sample. This improvement is likely attributed to the effective passivation of deep-level defects at its buried interface triggered by the aggregated TFA, as shown in **Fig. 2g**. Additionally, microscopically

red-shifted emission peaks (especially for the TFA sample) were also observed for corrosion inhibitor samples. This phenomenon is mainly ascribed to their effective compensation of tensile strain as well as the elimination of non-radiation carrier recombination centers.

Question 9: *Please discuss in detail the origin of strain changes in the perovskite films.*

Response: Thank you for this insightful suggestion regarding the origin of strain changes in our perovskite films. The observed strain variations arise from a complex interplay between deprotonation suppression and crystallization dynamic, which we have already elaborately elaborate in our revised manuscript as follows:

These results underscored the potential efficacy of corrosion inhibitors in alleviating undesirable residual strain, especially evident in the case of the MTFA sample, whose residual strain was drastically diminished to +7.71 MPa, compared to +44.30 MPa for the pristine sample and −17.82 MPa for the TFA sample. This favorable strain relaxation is likely triggered by synergistic effects, particularly suppressed protonation, which helps maintain the stoichiometric balance of PPS and reduce defect-induced lattice distortion, along with moderated crystallization dynamics that promote a more homogeneous and strain-balanced perovskite lattice[69,71,75,76]. Notably, the near-strain-relaxed state acquired within the MTFA sample warrants special attention owing to its progress in optimizing carrier dynamic, preserving an undistorted lattice structure and boosting intrinsic stability.

Reviewer #2: *The manuscript by Professor Zhang and colleagues presents an approach to enhance the stability of perovskite precursor solutions and the resulting perovskite solar cells through the incorporation of MTFA. While the work addresses an important challenge in perovskite device processing, the study lacks essential mechanistic clarity, quantitative evidence, and sufficient methodological detail to substantiate the claims. Several key experimental explanations, data analyses, and supporting discussions are incomplete or insufficiently justified. Substantial revisions, additional experiments, and clearer presentation of results would be required to reach the level of rigor expected for publication in Nature Communications.*

Response: Thank you for your comprehensive and critical assessment of our work. We acknowledge your concerns regarding mechanistic clarity and quantitative evidence. In our point-by-point response below and the substantially revised manuscript, we have undertaken extensive revisions, performed key additional experiments, and provided deeper analyses to address these concerns and meet the high standards expected for publication.

Question 1: *In Equation 1, water appears as a reactant. The authors must clarify the origin of this water molecule. If it originates from ambient moisture, then the precursor lifetime is highly dependent on humidity exposure. Additionally, the release kinetics of volatile CH_3OH and the corrosion inhibitor TFA should be quantified or estimated to assess the precursor shelf life. Without this information, the stability mechanism remains speculative.*

Response: Thank you for your kind suggestions for further improving our manuscript regarding the origin of water molecule and the hydrolysis kinetics of MTFA additive. We fully agree that addressing these points is crucial to move the mechanism from conceptual to quantitatively substantiated. We have thoroughly addressed these concerns by providing additional experimental data in **Fig.S10-11** and **Table S3**. Declaring that the water molecule in hydrolysis reaction is mainly originated from the ambient moisture due to the highly hygroscopic nature of polar aprotic solvents, while the release kinetics of volatile CH3OH and the corrosion inhibitor TFA was also revealed in our revised manuscript via time-dependent NMR spectra. The specific

discussion in our revised manuscript is as follow:

Ultimately, the superior deprotonation inhibition state is acquired by the MTFA-doped PPS sample as aging time extended capitalizing on its gradually released corrosion inhibitor of TFA via hydrolysis reaction in chemical formula (5). Therein, the trace amounts of water essential for MTFA hydrolysis are predominantly introduced through the highly hygroscopic nature of polar aprotic solvents (Dimethyl Sulfoxide (DMSO) and N,N-dimethylformamide (DMF)) as depicted in **Table S3**, both the DMF and DMSO declared inelegant hygroscopicity after 10 days atmospheric storage (~25 °C and ~35% RH).

$$CF_3COOCH_3 + H_2O \rightarrow CF_3COOH + CH_3OH \quad (5)$$

This could facilitate the suppressed deprotonation of PSS while simultaneously preventing the undesirable competitive proton-transfer induced by superfluous TFA^-, as evidenced by the progressively increasing CH_3OH signal over aging duration in **Fig. S10**, the correspondingly mirrored decreasing MTFA signals in **Fig. S11**, and the reducing PH values in **Fig. S8b**. Notably, an upward reversal in DoD values for all MTFA-doped PPS samples were occurred between the 20th and 30th days derived from the approaching depletion of MTFA, as confirmed by the partial enlarged ^1H NMR spectra in **Fig. S11**. Overall, these finding determined that despite the TFA initially achieved a conspicuous deprotonation inhibition, the competitive proton-transfer from HI to superfluous TFA^- remains a critical factor in prolonging the duration and violence of deprotonation process, thereby resulting in a relatively higher DoD in relevant aging PPS. Whereas, replacing TFA with MTFA has effectively mitigated this unacceptable prolongation of deprotonation by enabling the gradual released TFA, which avoiding the unpopular competitive proton-transfer induced by superfluous TFA^- as well as extending the deprotonation suppression period of MTFA-doped PPS to over 20 days.

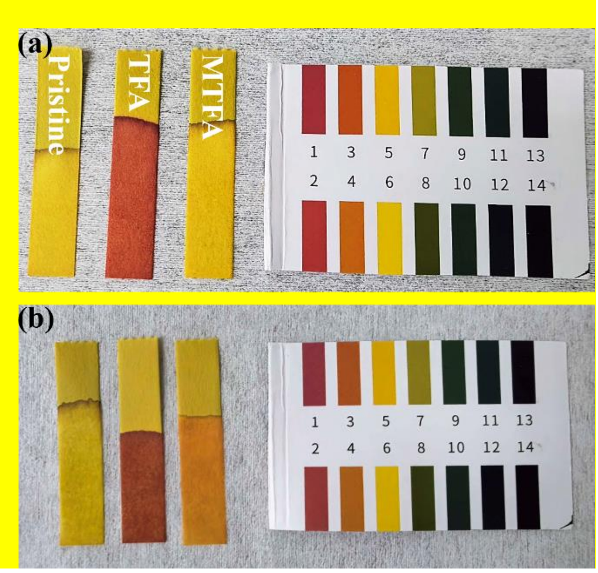

Figure S8. The pH tests of the **(a)** fresh and **(b)** aged pristine/TFA/MTFA-doped PPS, with both TFA and MTFA doped at a concentration of 20 μL/mL.

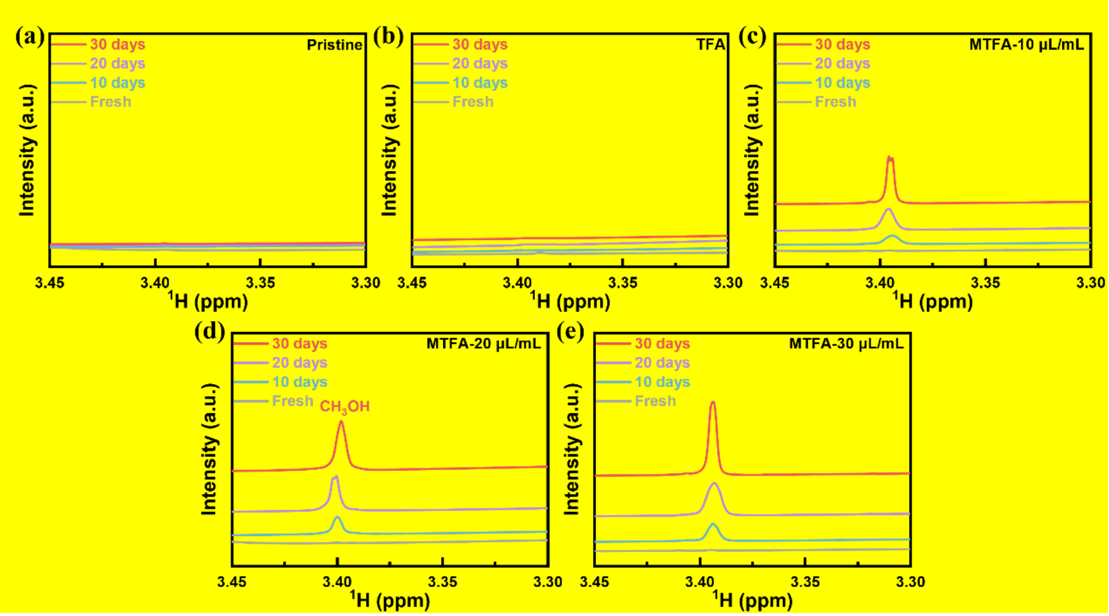

Figure S10. The partial enlarged ^{1}H NMR spectra indexed to the CH_3OH of the pristine/TFA/MTFA-doped PPS (DMSO-d_6).

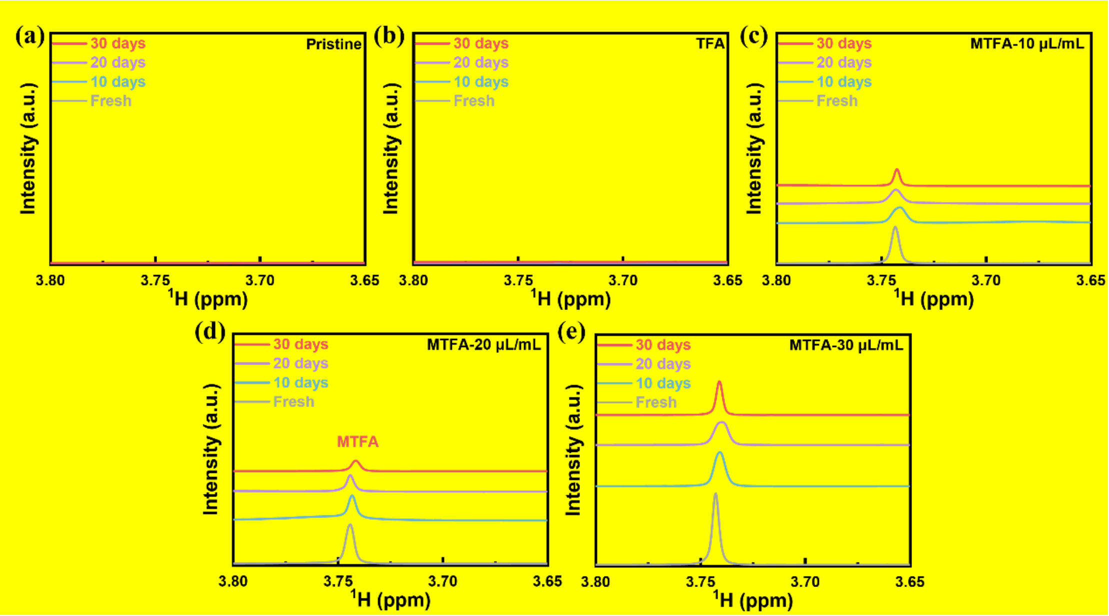

Figure S11. The partial enlarged ^{1}H NMR spectra indexed to the MTFA signal of the pristine/TFA/MTFA-doped PPS (DMSO-d_6).

Table S3. The water content of polar aprotic solvents with different compositions before and after hygroscopic aging, as determined by Karl Fischer titration.

DMF: DMSO	1: 0 (%)	1: 1 (%)	0: 1 (%)
Fresh	0.03	0.02	0.02
30 days	1.11	0.95	1.09

Question 2: *The effect of MTFA concentration on precursor shelf life and device performance is not sufficiently discussed. The authors should define the optimal concentration window and identify upper and lower limits that maintain stability and device efficiency. Further, it should be clarified whether this stabilization strategy is generalizable to different perovskite compositions (including bromide-rich wide-band-gap formulations) or is limited to the specific system studied.*

Response: We sincerely thank the reviewer for proposing these two crucial points regarding the concentration dependence and universality of our MTFA deprotonation suppression strategy. These aspects are essential for evaluating the practical utility and broader applicability of our approach. In our revised manuscript, we have conducted systematic experiments to address both points, and the results are now comprehensively

discussion in the revised manuscript section of "**Evolution of Perovskite Precursor Solutions Deprotonation**" and "**Photovoltaic Performance Evaluation of PSCs**".

Point 1: Optimal MTFA Concentration Window:

The concerns regarding the definition of the optimal MTFA concentration proposed by the reviewer are indeed essential and we have systematically evaluated three MTFA concentrations (10, 20, and 30 μL/mL) in our revised manuscript. As shown in **Table S1** and **Fig. S3-S6**, the deprotonation suppression efficacy is positively correlated with MTFA concentration during the entire aging period, while the strongest deprotonation suppression was observed in the 30 μL/mL MTFA-doped PPS. However, optimal PPSs stability must coincide with superior device performance. The champion PCEs of freshly prepared MTFA-doped devices (10, 20, and 30 μL/mL) were 25.66%, 26.24%, and 25.70% (**Table S4**) respectively. Obviously, although the 30 μL/mL MTFA-doping PPS declared a superior deprotonation suppression efficacy, the 20 μL/mL MTFA-doping device have achieved the highest photoelectric performance parameters. Therefore, 20 μL/mL is identified as the optimal concentration, not only extending the shelf life of PPS (evidenced by the 22-day-aged PPS still delivering a champion PCE of 26.35%, **Table S5**), but also avoiding the disordered crystallization associated with higher additive loads.

Point 2: Universality to Different Perovskite Compositions:

To verify the universality of the MTFA doping strategy in PPS deprotonation suppressing, we applied the MTFA assisted deprotonation suppression strategy to a bromide-rich WB perovskite PPS with the composition of $Cs_{0.15}FA_{0.65}MA_{0.20}Pb(I_{0.80}Br_{0.20})_3$ (Eg ≈ 1.68 eV). Concretely, NMR spectra in **Fig. S17-S20** demonstrated that the DoD values of the MTFA-doped WB PPS decreased initially and then subsequently increased with day 20th as the dividing line, accompanied by the mirror ascended and descended signals of MTFA and CH_3OH, confirming the same hydrolysis-controlled TFA release mechanism. Therefore, demonstrating that the MTFA assisted deprotonation suppression strategy is not only limited to a specific composition, but also applicable to all FA-based perovskite precursors where

deprotonation is a concern in deteriorating the shelf life of PPS, thereby declining the performance of relevant PSC, including those with different halide ratios and bandgaps. Overall, in our revised manuscript, we have established the optimal MTFA doping concentration as 20 μL/mL and verified the universality of our approach.

Question 3: *The XPS peak assignments in Fig. 1k–m requires correction and clarification. The binding energy position of Pb 4f7/2 must appear at lower energy than Pb 4f5/2. Peak identification should be clearly labelled on the spectra, and the corresponding discussion revised accordingly.*

Response: We sincerely thank the reviewer for catching this critical error in our original XPS figure presentation. We deeply apologize for the mistake and completely corrected the mistake as follows:

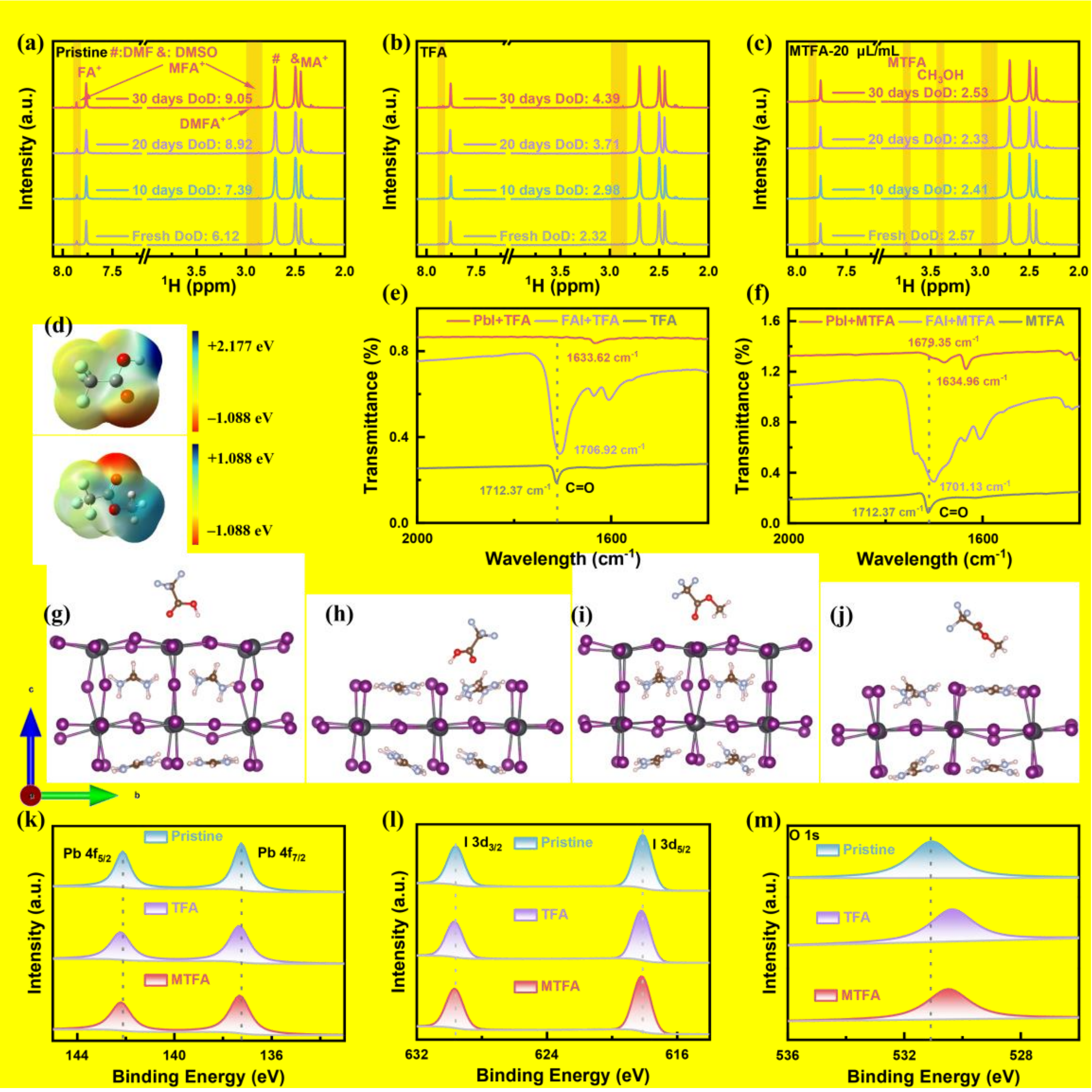

Figure 1. ^{1}H NMR spectra of **a)** pristine, **b)** TFA and **c)** 20 μL/mL MTFA PPSs at

different aging periods, with all spectra normalized to the intensity of the DMSO-d_6 signal as an internal reference. **d)** Electrostatic potential distribution (ESP) of the TFA (above) and MTFA (below). FTIR spectra of **e)** pure TFA, TFA+FAI and TFA+PbI_2 powders; **f)** pure MTFA, MTFA+FAI and MTFA+PbI_2 powders. Cell structure of TFA interacted with **g)** Pb-I framework and **h)** FA as well as MTFA interacted with **i)** Pb-I framework and **j)** FA. XPS spectra of **k)** Pb 4f, **l)** I 3d and **m)** O1s signals for perovskite films with different corrosion inhibitors.

The intricate chemical interplay between corrosion inhibitors and perovskite was further inquired through X-ray photoelectron spectroscopy (XPS) analysis. As depicted in **Fig. 1k-l** and **Fig. S23**, two dominant diffraction peaks positioned at 142.20 and 137.33 eV were associated with the Pb $4f_{5/2}$ and Pb $4f_{7/2}$ signals of pristine films respectively.

Question 4: *Stability-testing conditions must be explicitly stated in the figures, including humidity level, temperature, illumination conditions, and whether the devices were encapsulated. Without these parameters, the stability data cannot be meaningfully interpreted.*

Response: We sincerely thank you for this critical comment regarding the reporting of stability testing conditions. We fully agree that precise and complete environmental parameters are essential for the meaningful interpretation and reproducibility of stability data. We apologize for the omission of these details in the original manuscript and stated all relevant stability-testing conditions in the respective figure captions of the revised manuscript as follows:

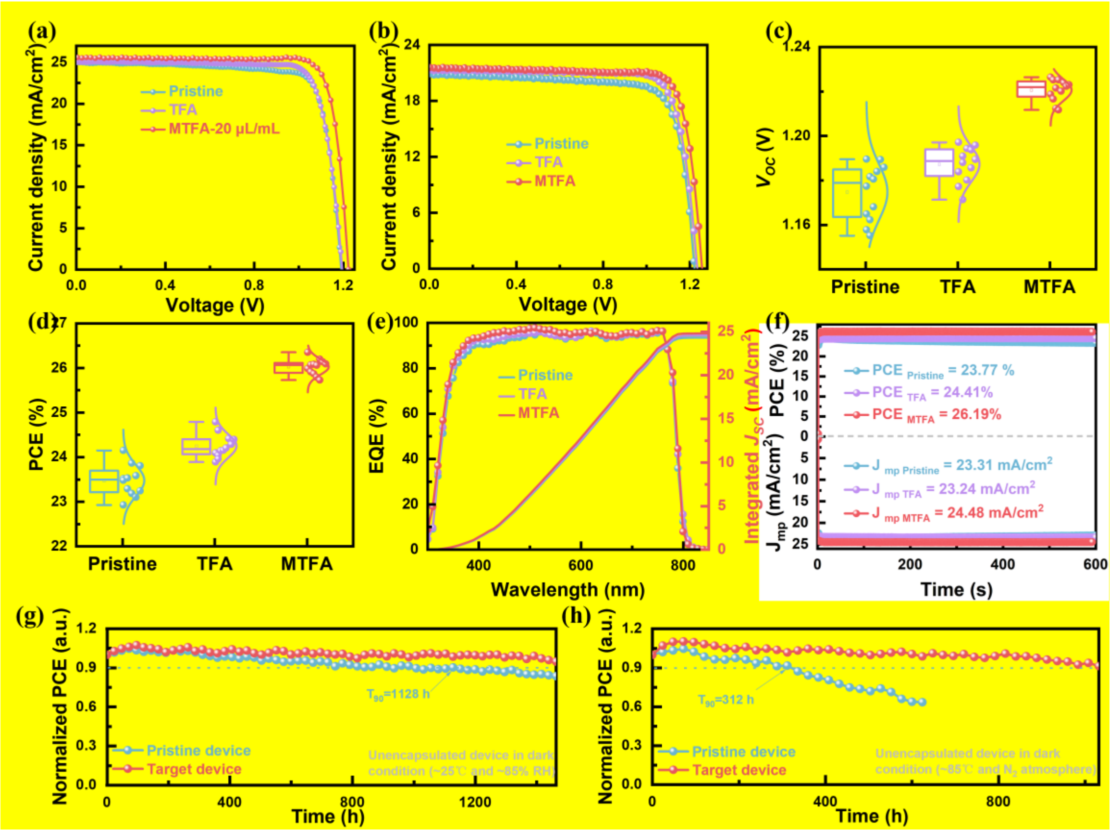

Figure 5 (a) *J*-*V* curves of champion PSCs with active area of 0.09 cm^2. **(b)** *J*-*V* curves of champion WB PSCs with active area of 0.09 cm^2. **(c)** PCE and **(d)** V_{oc} statistic distributions of 12 independent pristine device and corrosion inhibitor devices. **(e)** EQE spectrum and **(f)** Steady-state output performance of the pristine device and corrosion inhibitor device. **(g)** moisture stability and **(h)** thermal stability test result of the pristine device and MTFA device.

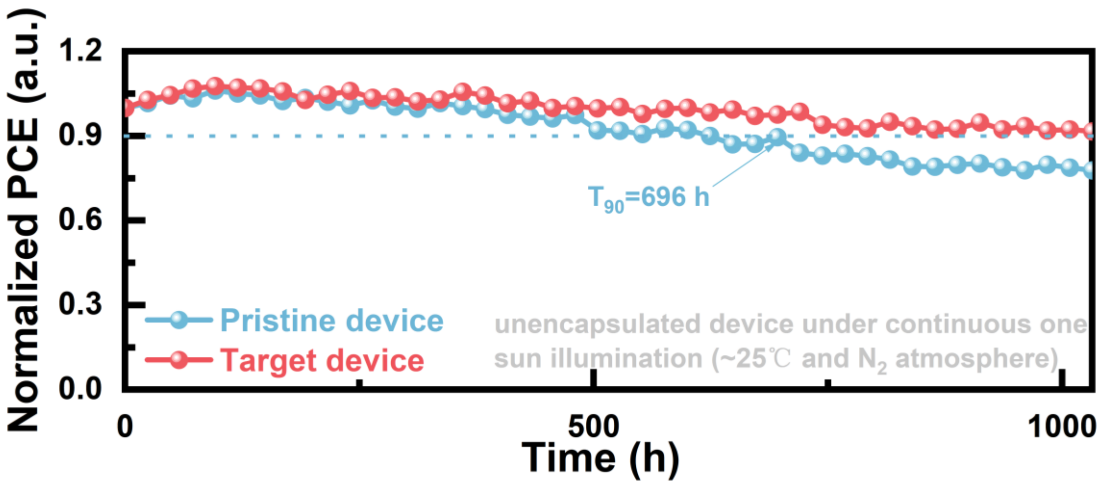

Figure S38. MPP tracking test of unencapsulated PSCs under one sun irradiation in a

~25 ± 2 °C N_2 atmosphere.

Question 5: *All stability curves show an initial improvement in PCE before degradation. The authors should provide an explanation for this behaviour, supported by analysis or references.*

Response: Thanks for your professional comment of our manuscript as well as kind suggestions to further improve our work. The PCE of PSCs exhibits a modest increase during the initial testing stage of all stability testing, followed by a sustained decline. The origins of this phenomenon are intricate and multifaceted, involving factors such as light/thermal induced variation of stresses, ion migration, structural rearrangements, the emergence of light-induced traps, and interfacial charge accumulation. In response to your feedback, we have conducted a detailed analysis in the revised manuscript as follows:

Additionally, it is worth emphasizing that all stability curves exhibited an initial improvement in PCE during the initial stage of stability testing, followed by a gradual and sustained decline. The underlying mechanisms of this tendency are complex and multifaceted, potentially involving strain relaxation, light/thermal field-induced ion migration, and structural rearrangements[78,79]. Anyway, these findings collectively affirmed the efficacy of MTFA in enhancing device stability through improved crystal quality and increased hydrophobicity.

Question 6: *Minor corrections and presentation issues:*

(a) electrostatic surface potential (ESP) maps are presented in Figure 1d, whereas in text it is referred as Fig. 1c

(b) "Investigation on Perovskite Films Optoelectronic Characteristic" contains morphological analysis too hence the heading should be modified accordingly

(c) Figure 4d, in Nyquist plot, x- and y-axis values should be same making an square.

(d) Figure 4e, what are the values of voltage on X-axis?

(e) "As illustrated in Fig. 4d, the substantially reduced Jdark value of the MTFA device...." the dark J-V curves are illustrated in figure 4e. Similarly other figure numbering should be checked carefully.

(f) Figure 5a and b are giving the same information, one of them can be moved the supplementary file.

(g) Figure 5e, the EQE spectra are shown in percentage however the y-scale is only up to 1.

Response: Thank you for your meticulous review and for identifying these presentation issues. We have carefully addressed each point in the revised manuscript and figures as follows:

(a) Sequentially, the coordination affinity between two corrosion inhibitors and perovskite films was investigated comprehensive. Molecular dynamic calculation was initially implemented to shedding light on the intricate charge distributions of TFA, MTFA and HI through electrostatic surface potential (ESP) maps in **Fig. 1d** and **Fig. S21**.

(b) Morphology, Structural and Optoelectronic Properties of Perovskite Films

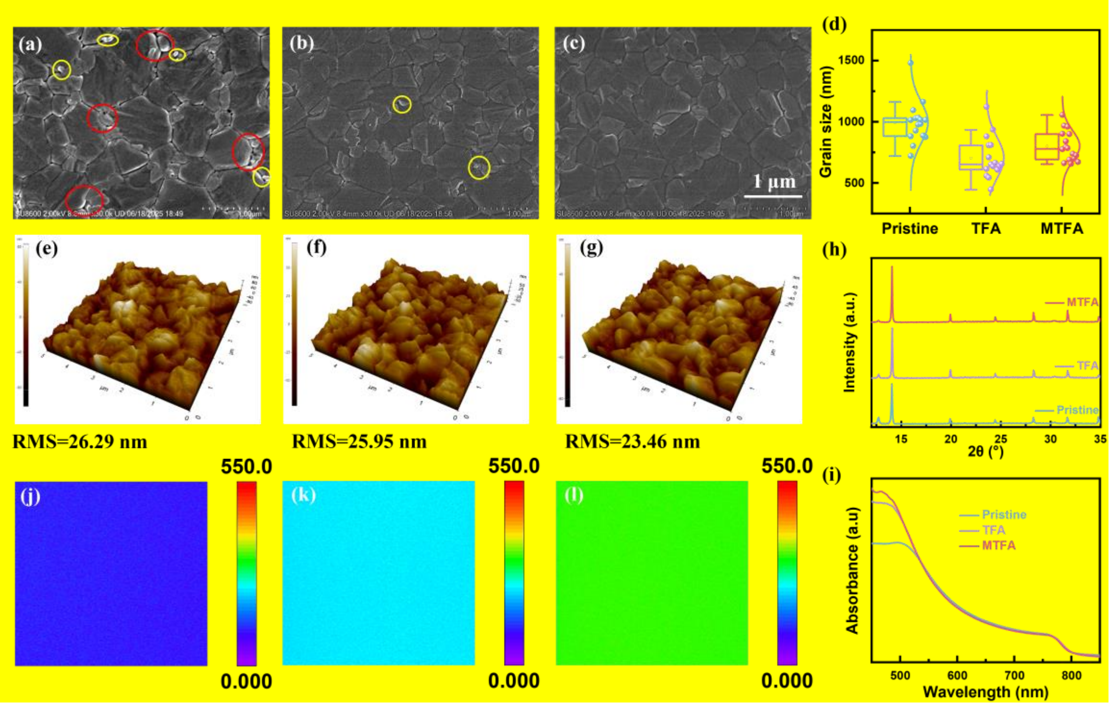

Figure 3. Top view SEM images of **(a)** the pristine sample, **(b)** the TFA sample and **(c)** the MTFA sample. **(d)** Statistical grain sizes of final perovskite films. AFM images and corresponding RMS values of **(e)** the pristine sample, **(f)** the TFA sample and **(g)** the MTFA sample. **(h)** XRD patterns and **(i)** UV–vis absorption spectrum of final

perovskite films. PL lifetime in PL mapping images of **(j)** the pristine sample, **(k)** the TFA sample and **(l)** the MTFA sample.

(c-e)

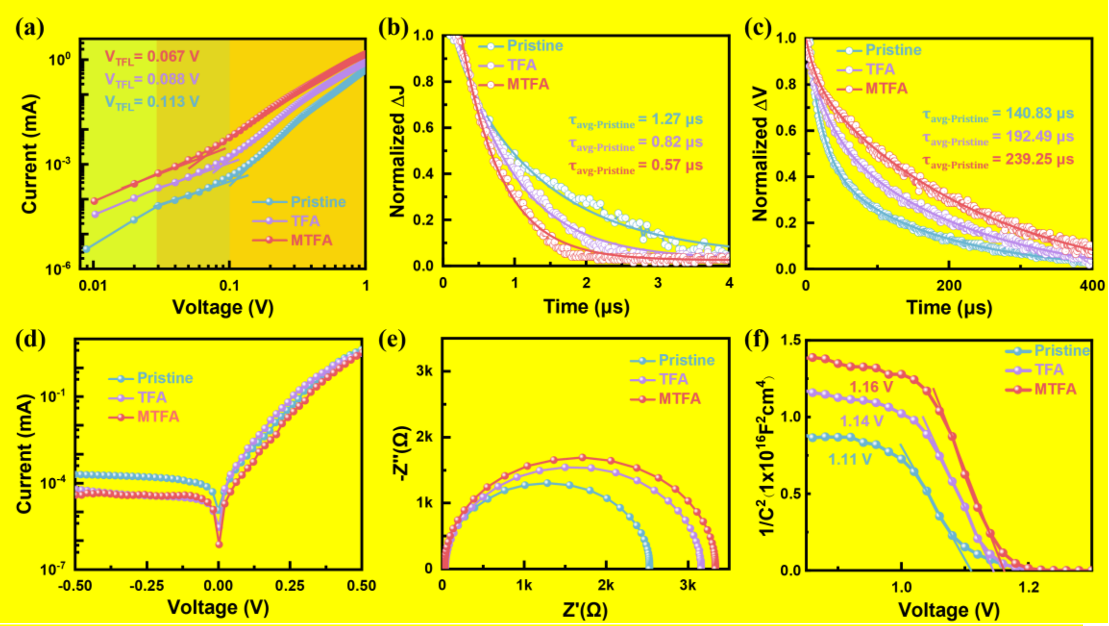

Figure 4. (a) SCLC analyses, **(b)** TPC curves, **(c)** TPV curves, **(d)** dark *J-V* curves, **(e)** Nyquist plots and **(f)** M-S plots of final PSCs.

(f-g)

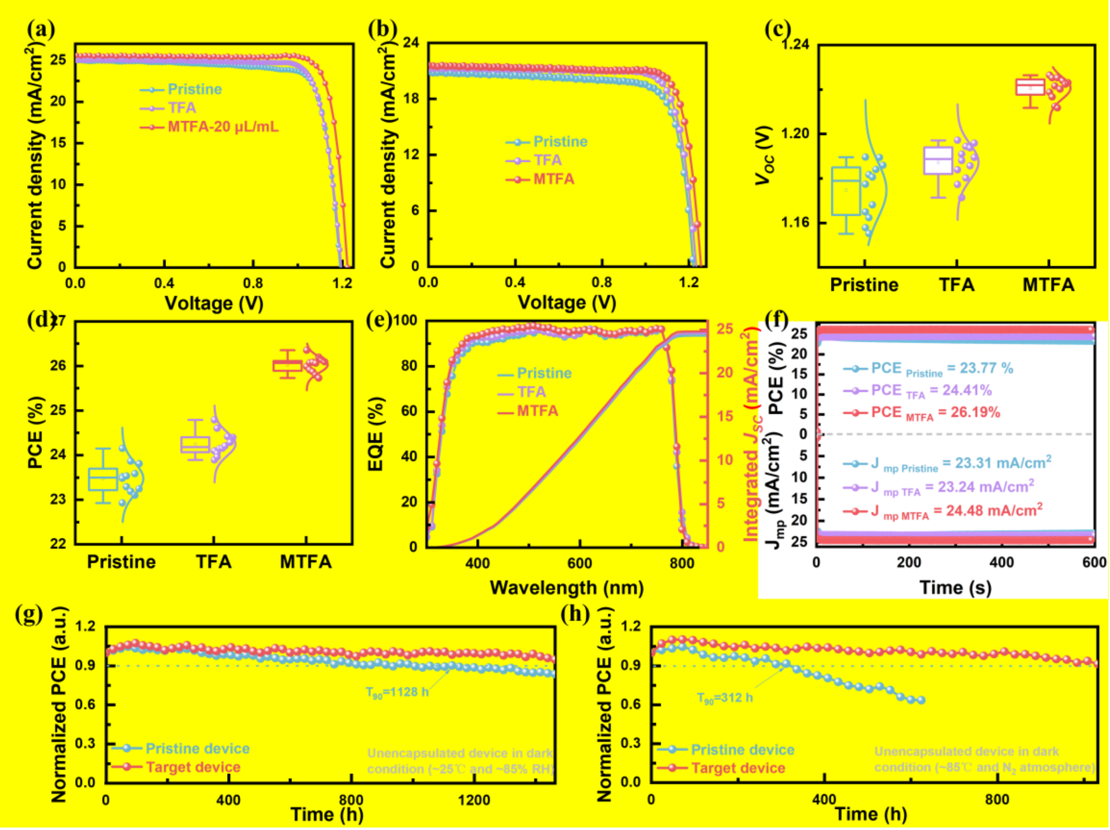

Figure 5 (a) *J*-*V* curves of champion PSCs with active area of 0.09 cm^2. **(b)** *J*-*V* curves of champion WB PSCs with active area of 0.09 cm^2. **(c)** PCE and **(d)** V_{oc} statistic distributions of 12 independent pristine device and corrosion inhibitor devices. **(e)** EQE spectrum and **(f)** Steady-state output performance of the pristine device and corrosion inhibitor device. **(g)** moisture stability and **(h)** thermal stability test result of the pristine device and MTFA device.

Reviewer #3: *The authors discussed the instability issue in FA-based perovskites caused by the irreversible deprotonation of the MA^+ cation inside the precursor solution. Thus, this deprotonation process limits the film quality and device performance. The study presents a MTFA–assisted strategy that avoids these limitations by slowly releasing –COOH groups via controlled hydrolysis, thereby reducing precursor deprotonation and enhancing the precursor shelf life. Additionally, authors reported that –COOCH3 and –COOH moieties modulate crystallization and passivate Pb2+ and halide vacancies, while CF3COO- species at the buried interface further improve carrier behaviour. This approach promises a more controlled and chemically stable route for FA-based perovskite fabrication.*

Response: We appreciate your positive comment on our manuscript of "*This approach promises a more controlled and chemically stable route for FA-based perovskite fabrication.*" as well as kind suggestions to further improve our work. We have revised our manuscript by responding your comments point-by-point as followings:

Question 1: *Approaches aimed at inhibiting precursor ageing in FA-based perovskites are not new and should be acknowledged. Relevant recent reports such as https://www.nature.com/articles/s41467-025-63776-6, https://www.sciencedirect.com/science/article/pii/S0927024825001461. The authors should clarify how their approach fundamentally differs from prior works.*

Response: We sincerely appreciate your constructive feedback and have carefully perused the two recommended references. We clarify that despite both recommended references exhibited efficiently deprotonation suppression efficacy in PPSs, their underlying mechanistic and aging kinetics remain totally different from our work. Concretely, deprotonation suppression of the two references was acquired depend on the directly incorporation of acidic additives (TFPH and SMP) and their proton donation capability, while the dynamics regulation of proton donation and the potential issue of competitive proton transfer still unclaimed. In contrast, our study had proposed an ingenious MTFA assisted deprotonation suppression strategy, which could acquire a eliminated competitive proton-transfer within PPS through its stepwise hydrolysis reaction and the gentle release of -COOH derived from trifluoroacetic acid (TFA)

byproduct. This approach effectively suppressed the deprotonation of relevant PPS and extended its shelf life to over 22 days Moreover, the robust affinity of $-COOCH_3$ or byproduct -COOH groups toward uncoordinated Pb^{2+} enabled the precise tuning of perovskite crystallization dynamic, while the residual CF_3COO^- species distributed at the buried interface of perovskite were considered to offer an additional improvement in film carrier behavior via the halogen vacancies passivation. In summary, our work presents a kinetically controlled PPS anti-aging strategy that fundamentally diverged from earlier approaches and references you recommended were already cited (reference 37 and 38) in the revised manuscript.

Question 2: *Can this MTFA-assisted strategy be extended to other FA-based compositions, including different bandgaps? Demonstrating broader applicability would significantly strengthen the study.*

Response: We thank you for raising this important point regarding the broader applicability of our MTFA-assisted deprotonation suppression strategy and realize the significance of this feature in strengthening the research value of this study. From this, we have explicitly tested the MTFA-assisted deprotonation suppression strategy on a bromide-rich WB FA-based perovskite with the composition of $Cs_{0.15}FA_{0.65}MA_{0.20}Pb(I_{0.80}Br_{0.20})_3$. Demonstrating that the MTFA assisted deprotonation suppression strategy is not only limited to a specific composition, but also applicable to all FA-based perovskite precursors where deprotonation is a concern in deteriorating the shelf life of PPS, thereby declining the performance of relevant PSC, including those with different halide ratios and bandgaps. Corresponding revision in our manuscript are as follow:

Therefore, chemical species evolution tests were further conducted on a bromide-rich wide-bandgap (WB) PPS with the composition of $Cs_{0.15}FA_{0.65}MA_{0.20}Pb(I_{0.80}Br_{0.20})_3$ to further verify the universality of the MTFA (20 µL/mL) doping strategy in PPS deprotonation suppressing, the bandgap of this perovskite materials is approximately 1.68 eV[58]. As depicted in **Fig. S17-19** and **Table S6**, DoD values of both the pristine and MTFA-doped WB PPS were strictly adhered to an evolutionary history analogous to that of the normal-bandgap PPS. Concretely, DoD values of the pristine WB PPS

exhibited a monotonic gradual increase across the entire aging period while those of the MTFA-doped WB PPS decreased initially and then subsequently increased with day 20th as the dividing line. The mirror ascending and descending signals of MTFA and CH_3OH in **Fig. S20** have further confirming the hydrolysis reaction of MTFA within PPS and affirming the superiority of MTFA-doping strategy in deprotonation suppression via controlled TFA release.

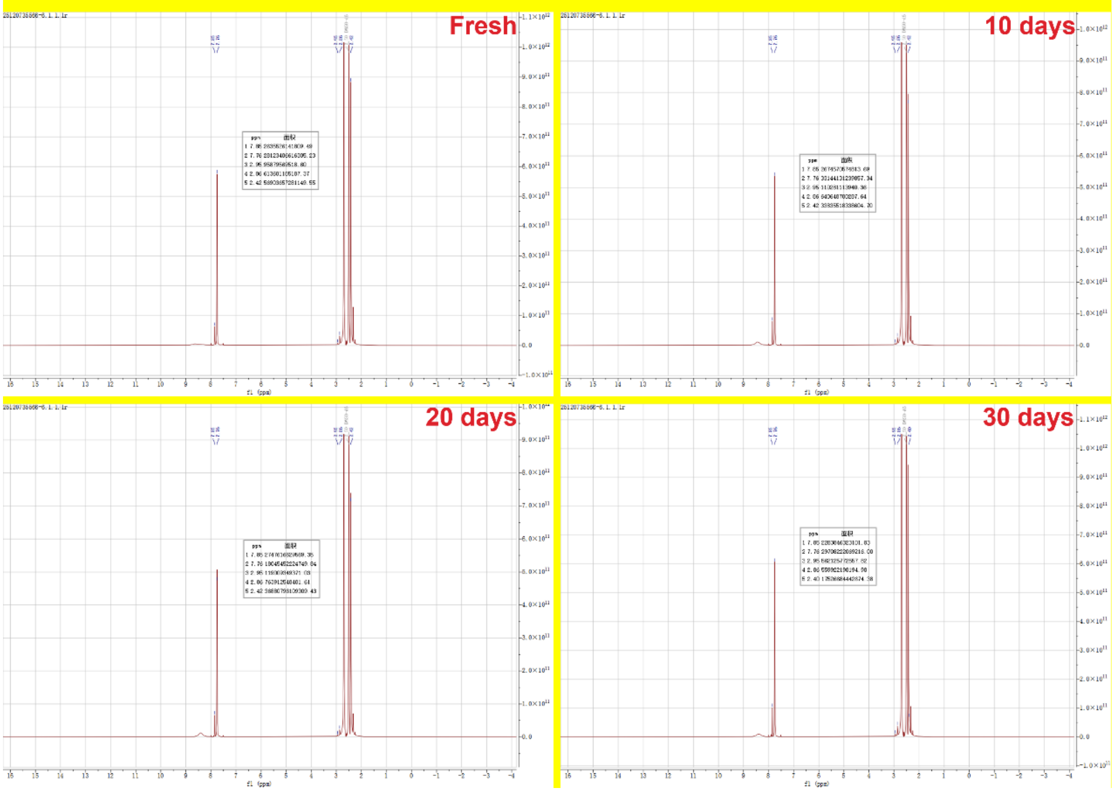

Figure S17. ^{1}H NMR spectra of the fresh and aged pristine WB PPS (DMSO-d_6) at different aging periods.

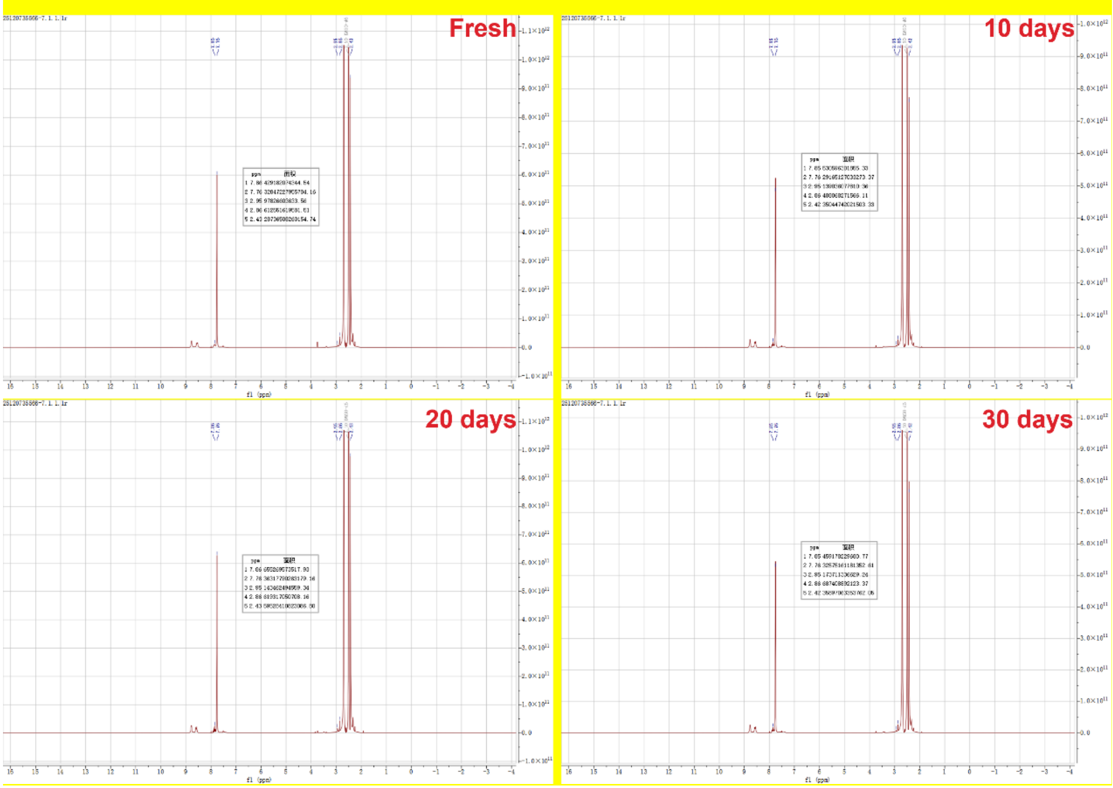

Figure S18. ^{1}H NMR spectra of the fresh and aged 20 μL/mL MTFA-doped WB PPS (DMSO-d_6) at different aging periods.

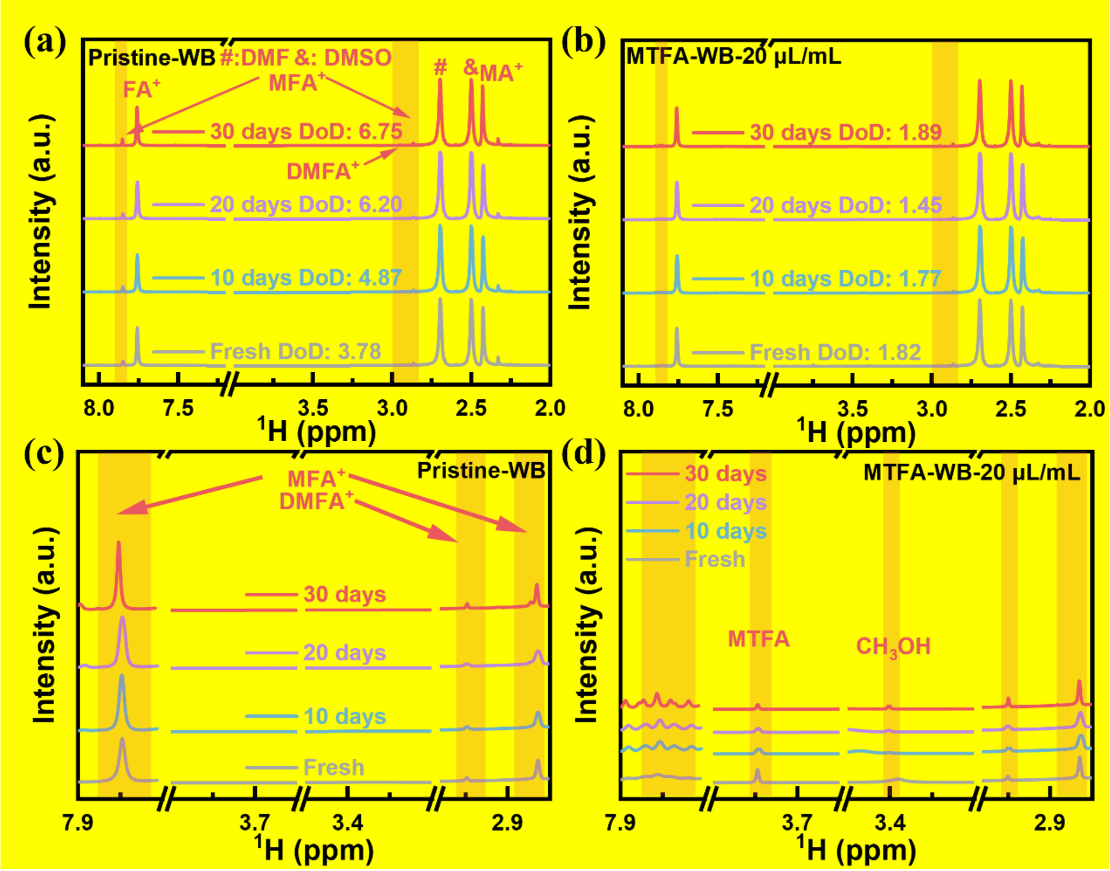

Figure S19. ^{1}H NMR spectra of **a)** pristine and **b)** 20 µL/mL MTFA-doped WB PPSs at different aging periods, with all spectra normalized to the intensity of the DMSO-d_6 signal as an internal reference. The partial enlarged ^{1}H NMR spectra indexed to MFA^+, $DMFA^+$, MTFA and CH_3OH signals of the fresh and aged **c)** pristine and **d)** 20 µL/mL MTFA-doped WB PPSs (DMSO-d_6), with all spectra normalized to the intensity of the DMSO-d_6 signal as an internal reference.

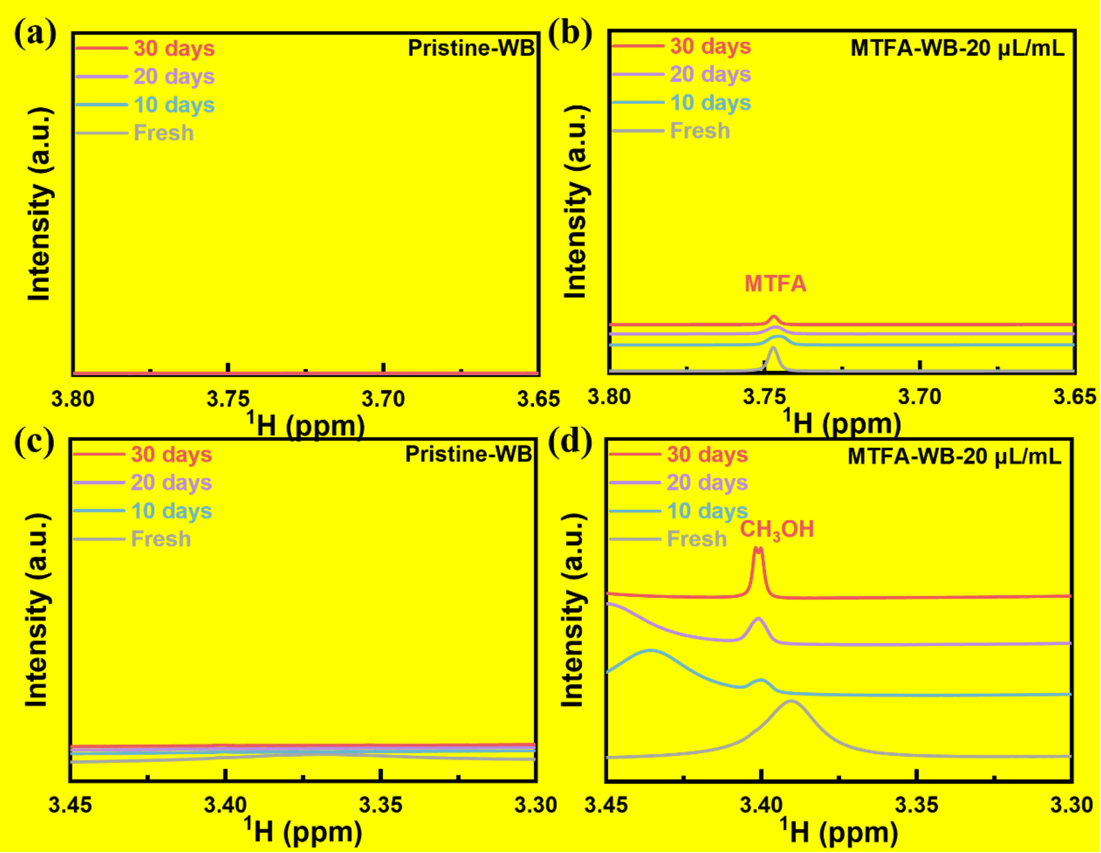

Figure S20. The partial enlarged ^{1}H NMR spectra indexed to the MTFA of the **a)** pristine and **b)** 20 µL/mL MTFA-doped WB PPSs (DMSO-d_6). The partial enlarged ^{1}H NMR spectra indexed to the CH_3OH of the **c)** pristine and **d)** 20 µL/mL MTFA-doped WB PPSs (DMSO-d_6).

Table S6. DoD value for the fresh and aged wide-band PPSs with and without MTFA.

	Pristine (%)	20 µL/mL MTFA (%)
Fresh	3.78	1.82
10 days	4.87	1.77

20 days	6.20	1.45
30 days	6.73	1.89

Additionally, the MTFA-assisted strategy for optimizing PSC performance is also applicable to the WB device. As illustrated in **Fig. 5b** and **Table S10**, a satisfactory PCE of 22.49% was acquired by the MTFA assisted WB PSCs, surpassing that to both the pristine (19.89%) and TFA assisted (21.44%) WB PSCs, confirming the universality of MTFA-assisted strategy in PPS deprotonation suppression and perovskite film crystallization dynamics regulation.

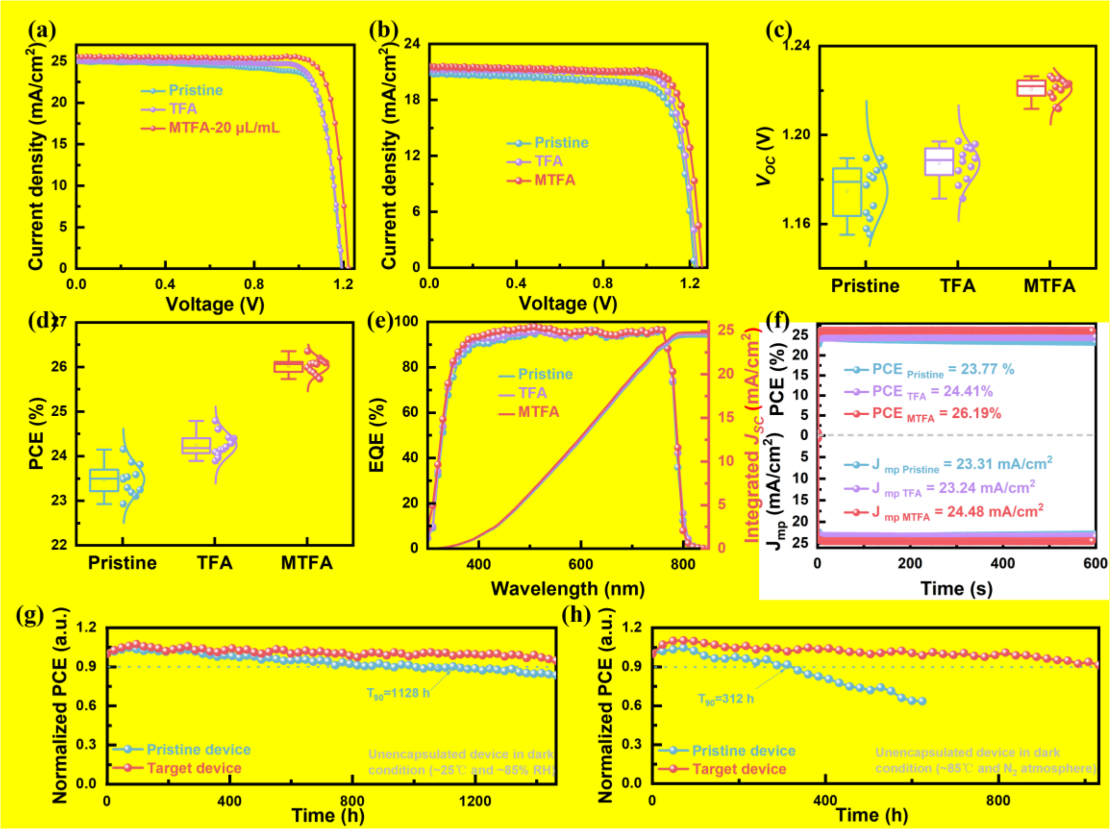

Figure 5 (a) *J-V* curves of champion PSCs with active area of 0.09 cm^2. **(b)** *J-V* curves of champion WB PSCs with active area of 0.09 cm^2. **(c)** PCE and **(d)** V_{oc} statistic distributions of 12 independent pristine device and corrosion inhibitor devices. **(e)** EQE spectrum and **(f)** Steady-state output performance of the pristine device and corrosion inhibitor device. **(g)** moisture stability and **(h)** thermal stability test result of the pristine device and MTFA device.

Table S10. Photoelectric performance parameters extracted from the *J-V* curves of wide bandgap devices.

Cells	J_{SC} (mA/cm^2)	V_{OC} (V)	FF (%)	PCE (%)
Pristine	20.92	1.22	77.93	19.89
TFA	21.35	1.23	81.63	21.44
MTFA	21.58	1.26	82.90	22.49

Question 3: *The table provided in the Supporting Information about DoD should be incorporated as a bar graph in Figure 1. As presented, the H-NMR spectra show minimal visible differences between TFA and MTFA, making quantitative comparison difficult.*

Response: We sincerely appreciate your insightful suggestion and fully concur that a quantitative comparison of the Degree of Deprotonation (DoD) is essential to vividly highlight the superior long-term deprotonation suppression efficacy of MTFA-based PPS relative to both TFA-treated and pristine PPSs. However, due to the spatial constraints imposed by the densely packed layout of **Fig. 1a–c**, integrating DoD values into a standalone bar graph proved impractical. As an elegant and informative alternative, we have directly annotated all DoD values above their respective NMR spectra. The corresponding revisions are presented as follows:

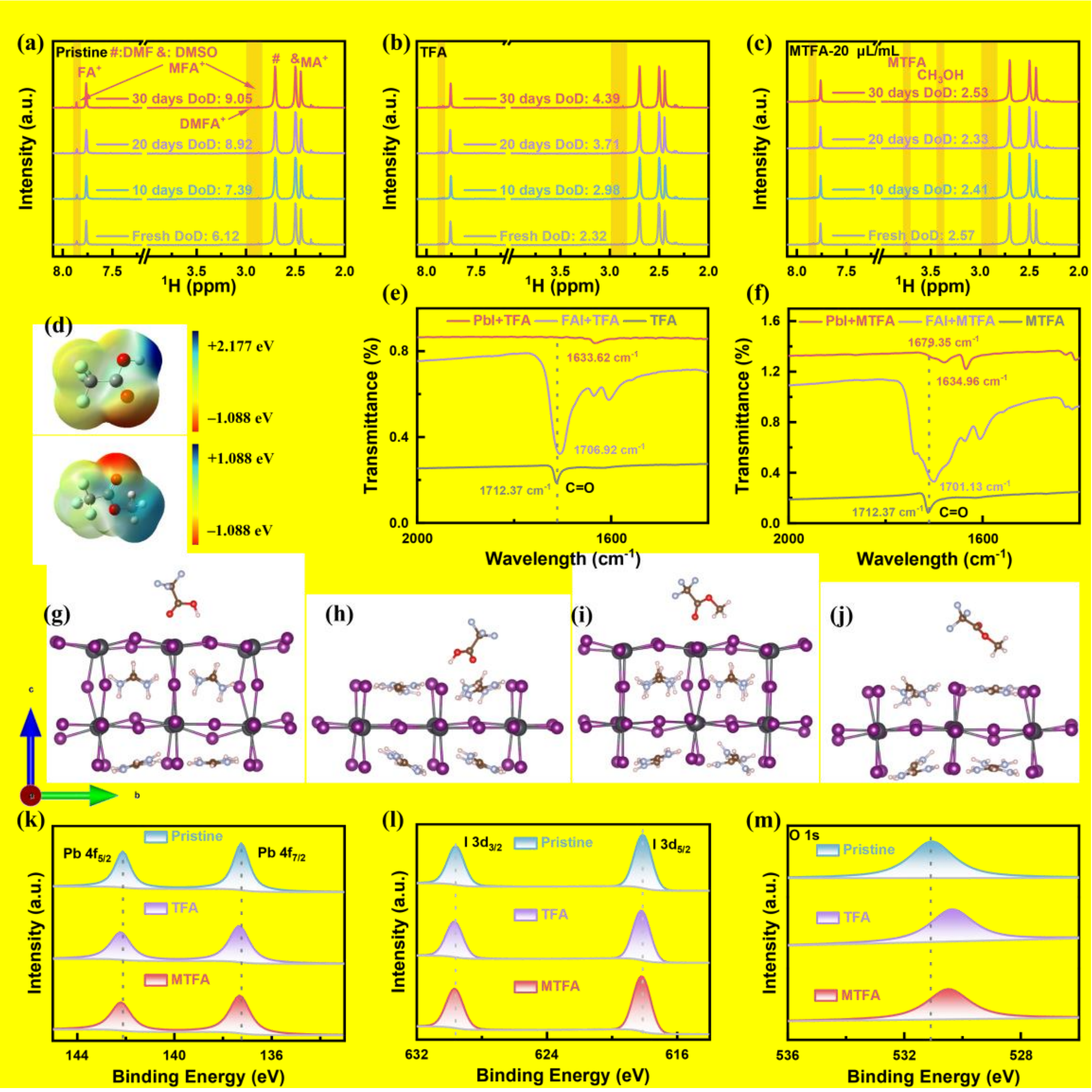

Figure 1. ^{1}H NMR spectra of **a)** pristine, **b)** TFA and **c)** 20 μL/mL MTFA PPSs at different aging periods, with all spectra normalized to the intensity of the DMSO-d_6 signal as an internal reference. **d)** Electrostatic potential distribution (ESP) of the TFA (above) and MTFA (below). FTIR spectra of **e)** pure TFA, TFA+FAI and TFA+PbI_2 powders; **f)** pure MTFA, MTFA+FAI and MTFA+PbI_2 powders. Cell structure of TFA interacted with **g)** Pb-I framework and **h)** FA as well as MTFA interacted with **i)** Pb-I framework and **j)** FA. XPS spectra of **k)** Pb 4f, **l)** I 3d and **m)** O1s signals for perovskite films with different corrosion inhibitors.

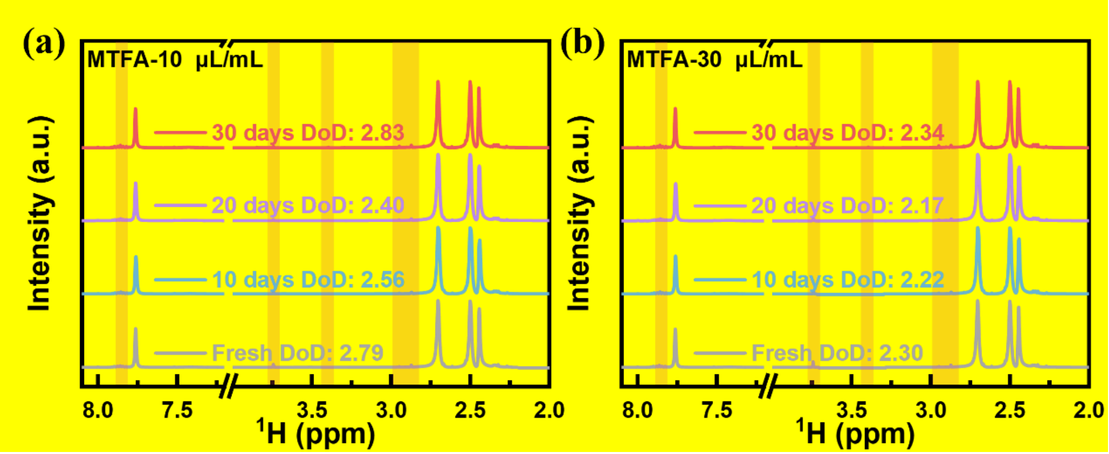

Figure S6. ^{1}H NMR spectra of **a)** 10 μL/mL MTFA and **b)** 30 μL/mL MTFA PPSs at different aging periods, with all spectra normalized to the intensity of the DMSO-d_6 signal as an internal reference.

Question 4: *The TRPL analysis is physically incorrect. The authors should revise this section and include excitation parameters (wavelength, fluence, repetition rate) directly in the figure caption. For guidance, see the reference papers below: https://advanced.onlinelibrary.wiley.com/doi/full/10.1002/aenm.202403279,* https://www.nature.com/articles/ncomms13941

Response: We sincerely thank you for your professional suggestion regarding our TRPL analysis and approve that providing complete experimental parameters is essential for accuracy the TRPL result in our manuscript, concurrently added excitation parameters in our revised manuscript as follows:

Steady-state PL and TRPL curves were measured using a FluoTime 300 spectrometer (PicoQuant, German) with wavelength of 532 nm, fluence of 2.38 μJ/cm^2 and repetition rate of 80 MHz.

Additionally, we have carefully studied both insightful references provided by the reviewer which offered a comprehensive discussion on power-law decays and photon recycling during the TRPL analysis process. We fully acknowledge the profound importance of these concepts for the accurate physical interpretation of TRPL data in PSCs. Whereas, on the one hand, all samples in our study were fabricated, measured, and analyzed consecutively under perfectly identical conditions. Any systematic effects such as photon recycling or instrumental response would affect all three datasets equally.

On the other hand, TRPL curves of our samples have not declared a definite power-law decay ($\phi_{PL} \propto t^{-2}$) as illustrated in **Fig. R1**, which is a hallmark of shallow-trap-dominated bimolecular recombination as discussed by Yuan et al. Instead, our TRPL curves was particularly accord with the bi-exponential decay trend (verified by the highly overlapping bi-exponential fitting curves and primitive curves of all samples) for the purpose of extracting an accurate τ_{avg}. Which was served as a widely accepted and well-established parameter for such comparative studies, as demonstrated in numerous high-impact papers such as: (https://www.nature.com/articles/s41467-025-67350-y), (https://www.nature.com/articles/s41467-025-66414-3), (https://www.nature.com/articles/s41467-024-51703-0) and (https://www.nature.com/articles/s41560-024-01471-4)

Notably, in our work, the primary objective of the TRPL measurement was to perform a semi-quantitative assessment of the relative non-radiative recombination losses among the pristine, TFA-doped, and MTFA-doped perovskite films under identical measurement conditions. Thus, although we approve the principles proposed in the references, we still respectfully argue that the comparative analysis presented in our initial manuscript remains physically feasible for its intended purpose.

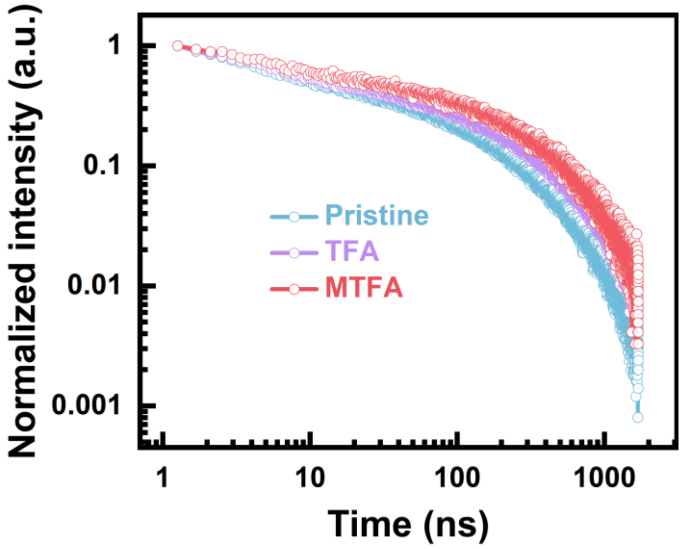

Figure R1. Normalized PL decays of perovskite films on a double-logarithmic scale measured using fluences of 2.38 μJ/cm^2.

Question 5: *In Figure 2 discussion part, it is stated that the subsequent investigations*

are based on aging precursors (how long is missing). If MTFA slows precursor ageing, the manuscript should also include device performance and film characterization from fresh and aged precursor solutions. As written, the study resembles a comparison of two additives rather than a precursor-stability analysis. The long-term stability of devices fabricated from fresh and aged solutions is also missing.

Response: We sincerely thank the reviewer for this incisive and profoundly insightful remark and acknowledge the reviewer is absolutely accurate. To unequivocally establish that our study is a comprehensive PPS stability investigation, rather than a superficial comparison of additives. It is essential to present a time-related, holistic framework that seamlessly integrates the evolution of solution aging, dynamic film characteristics, and resultant device performance. In direct response to this invaluable feedback, we have carried out an extensive series of new experiments and implemented sweeping revisions throughout the manuscript:

Furthermore, X-ray diffraction (XRD) characterization of perovskite films prepared with fresh or aged PPSs was also performed under the same sampling schedule to inquire into the influence of PPSs deprotonation degree on film crystalline characteristics, as well as confirming the accuracy of our above analysis. As illustrated in **Fig. S12-13**, the dominant diffraction signal observed at approximately 14.06° across all patterns is widely recognized as the (100) facet of the α-phase perovskite, serving as a key indicator for assessing the crystallinity of perovskite films. In contrast, the diffraction peak emerging at 12.70° is generally assigned to the unreacted PbI_2 within the perovskite films. Notably, both the pristine and TFA samples exhibited a steady decline in the (100) diffraction intensity, accompanied by a gradual yet unmistakable increase in the PbI_2 signal throughout the entire aging period, consistent with their time-dependent increased deprotonation degree as well as stepwise depletion of organic cation as revealed by our NMR analysis. However, all MTFA-doped samples established a steady ascend in the (100) peak intensity over the initial 20 days, along with a mirror decline in the PbI_2 signal, followed by a reversal thereafter, according with their deprotonation degree evolutionary trend. Especially, upon comprehensive screening of all XRD patterns, the perovskite film fabricated from MTFA-doped PPS

that underwent 20 days of aging has ultimately attained optimal crystalline, exhibiting the highest (100) peak intensity and a barely perceptible PbI_2 residue derived from the effective suppression of deprotonation and exquisite modulation of crystallization dynamics, which collectively contribute to superior device performance.

Subsequently, the evolution of PCE extracted from the current density-voltage (*J-V*) curves of PSCs assembled with aforementioned perovskite films were plotted in **Fig. S14**. This analysis validated both the aging time-dependent deprotonation extent of PPSs incorporating different corrosion inhibitors and crystalline of relevant perovskite films, corroborating the findings without any contradiction as well as precisely pinpointed the peak performance of MTFA-doped PSCs was achieved on the 20th, 22nd, and 24th days as the MTFA doping concentration of 10/20/30 μL/mL. Furthermore, *J-V* curves of PSCs fabricated with freshly prepared and 22 days aging PPSs were displayed in **Fig. S15-16**, with corresponding photoelectric performance parameters enumerated in **Table S4-5**, indicating that the champion PCE for both the fresh and aged PSCs were achieved by the 20 μL/mL MTFA-doped device with values 26.24% and 26.35% respectively.

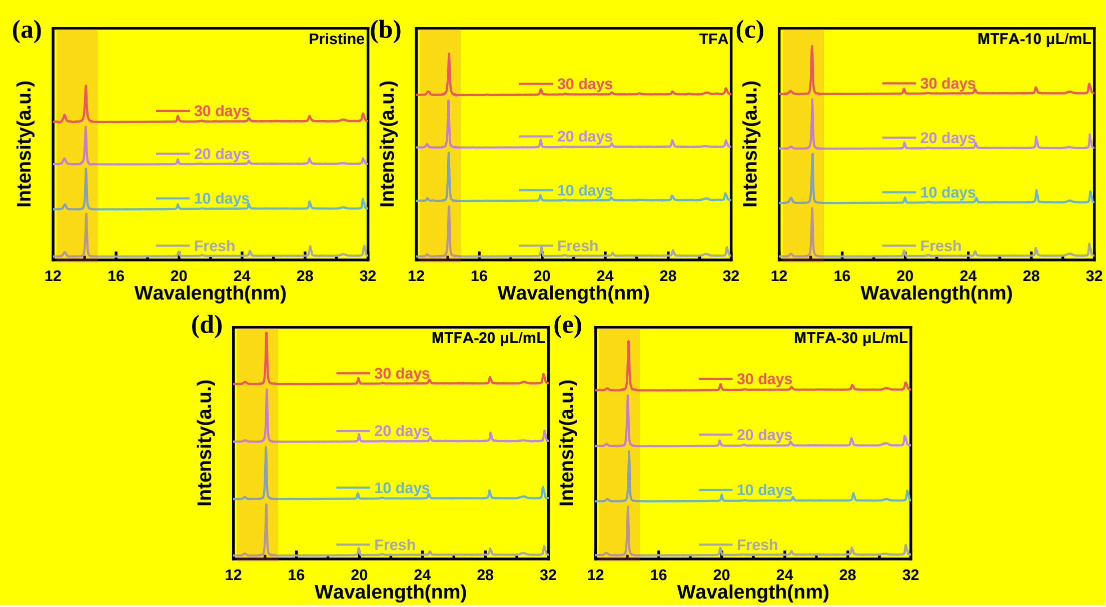

Figure S12. XRD patterns evolution of perovskite films prepared from fresh or aged PPS with and without TFA/MTFA as a function of aging time.

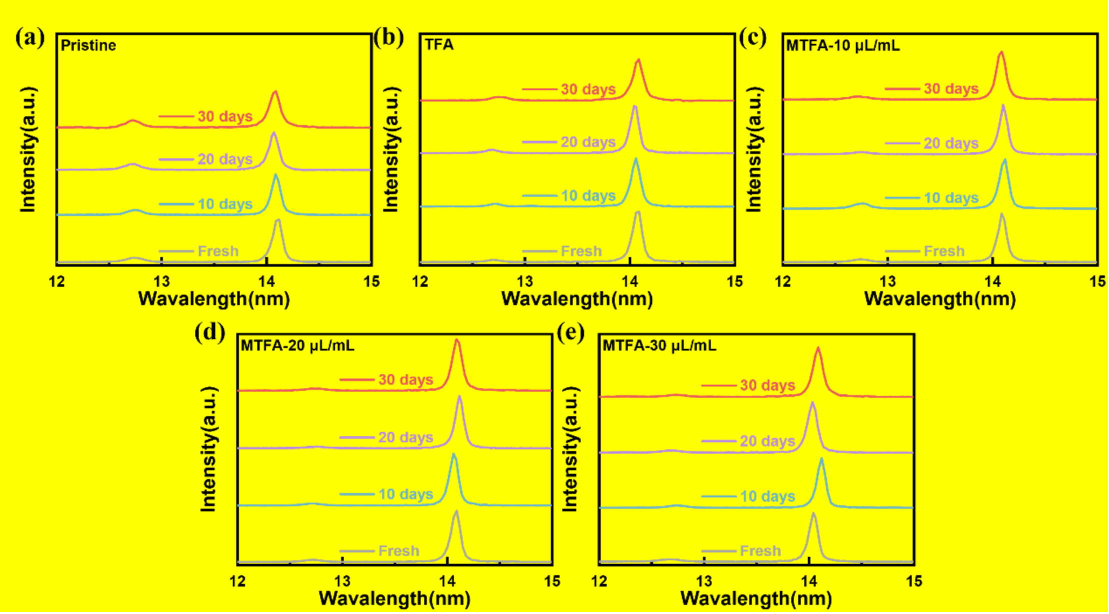

Figure S13. Partially enlarged PbI_2 and (100) diffraction peaks of perovskite films prepared from fresh or aged PPS with and without TFA/MTFA as a function of aging time.

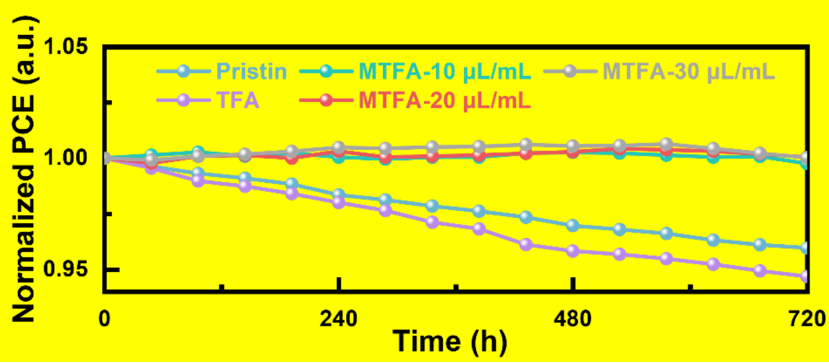

Figure S14. The PCE evolution trend of PSCs with the extend of aging time.

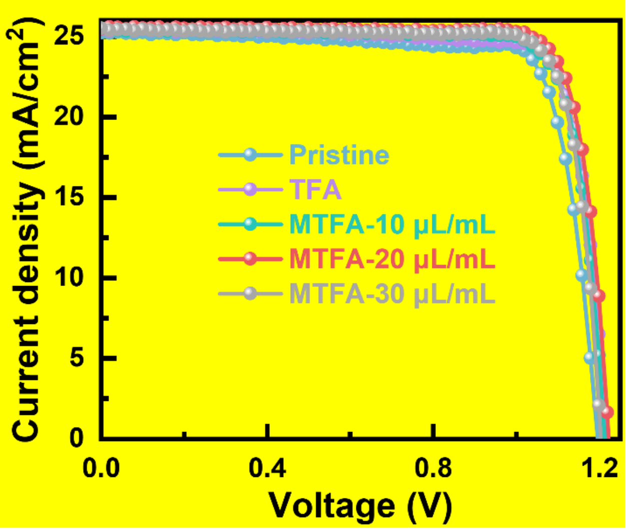

Figure S15. *J-V* curves of champion PSCs with freshly prepared PPSs.

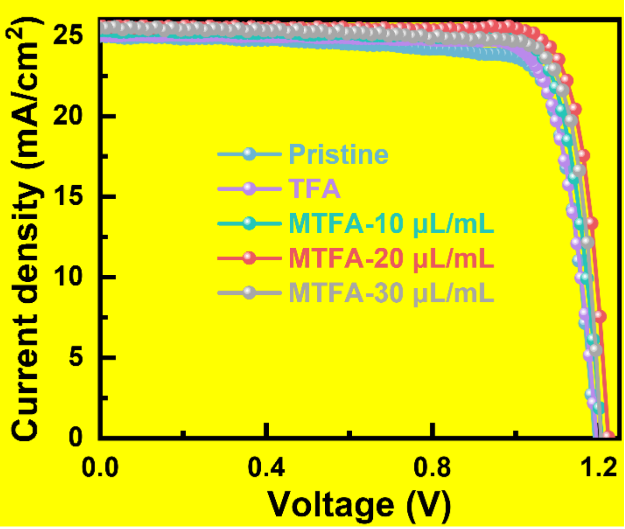

Figure S16. *J-V* curves of champion PSCs with aging PPSs.

Table S4. Photoelectric performance parameters extracted from the *J-V* curves of PSCs assembled using freshly prepared PPSs.

Cells	J_{SC} (mA/cm^2)	V_{OC} (V)	FF (%)	PCE (%)
Pristine	25.27	1.20	82.47	24.95
TFA	25.45	1.22	83.55	25.91
MTFA-10 µL/mL	25.48	1.21	83.01	25.66
MTFA-20 µL/mL	25.59	1.22	83.81	26.24
MTFA-30 µL/mL	25.51	1.20	83.69	25.70

Table S5. Photoelectric performance parameters extracted from the *J-V* curves of PSCs assembled using PPSs aged for 22 days.

Cells	J_{SC} (mA/cm^2)	V_{OC} (V)	FF (%)	PCE (%)
Pristine	25.16	1.19	80.69	24.15
TFA	25.30	1.20	81.85	24.79
MTFA-10 µL/mL	25.51	1.21	83.18	25.72
MTFA-20 µL/mL	25.62	1.23	83.87	26.35
MTFA-30 µL/mL	25.55	1.21	83.78	25.84

Table S6. DoD value for the fresh and aged wide-band PPSs with and without MTFA.

***Question 6:** Figure 5a and b are repetitive?*

Response: We sincerely appreciate your keen observation concerning the visual similarity between Figure 5a and 5b in our original manuscript. In response to this valuable feedback, we have thoughtfully restructured Figure 5 in the revised manuscript. The updated Figure 5a now exclusively showcases the *J-V* curves of the champion narrow-bandgap devices and the original Figure 5b has been replaced by the *J-V* curves of the champion wide-bandgap devices. Corresponding revision are as following:

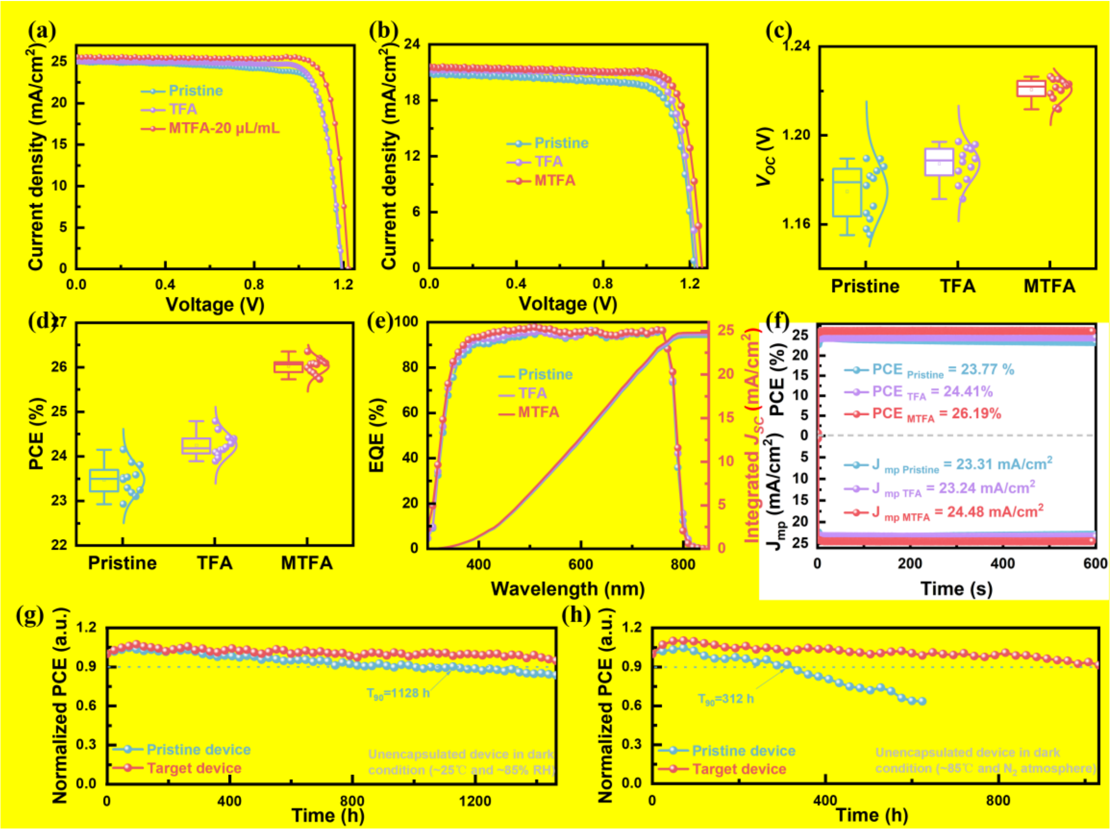

Figure 5 (a) *J-V* curves of champion PSCs with active area of 0.09 cm^2. **(b)** *J-V* curves of champion WB PSCs with active area of 0.09 cm^2. **(c)** PCE and **(d)** V_{oc} statistic distributions of 12 independent pristine device and corrosion inhibitor devices. **(e)** EQE spectrum and **(f)** Steady-state output performance of the pristine device and corrosion inhibitor device. **(g)** moisture stability and **(h)** thermal stability test result of the pristine device and MTFA device.

***Question 7:** The mapping between pH values and sample conditions is unclear; please*

specify which reading corresponds to which solution.

Response: We thank the reviewer for pointing out this lack of clarity. We have revised the corresponding Figure (**Fig. S8** in the Supplementary Information) as follows:

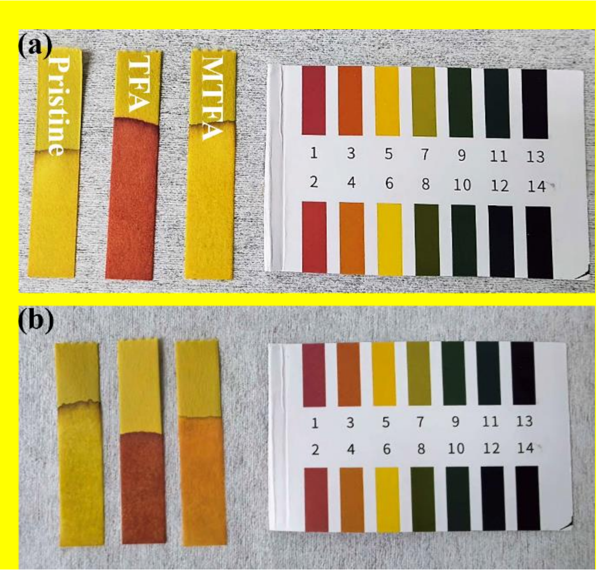

Figure S8. The pH tests of the **(a)** fresh and **(b)** aged pristine/TFA/MTFA-doped PPS, with both TFA and MTFA doped at a concentration of 20 μL/mL.

Question 8: *In Figure 2, panels (a) and (b) mentioned in the caption are missing from the figure and must be corrected.*

Response: We sincerely thank you for identifying this critical error in our figure assembly. And we have thoroughly rearrangement Figure 2 in our revised manuscript as follows:

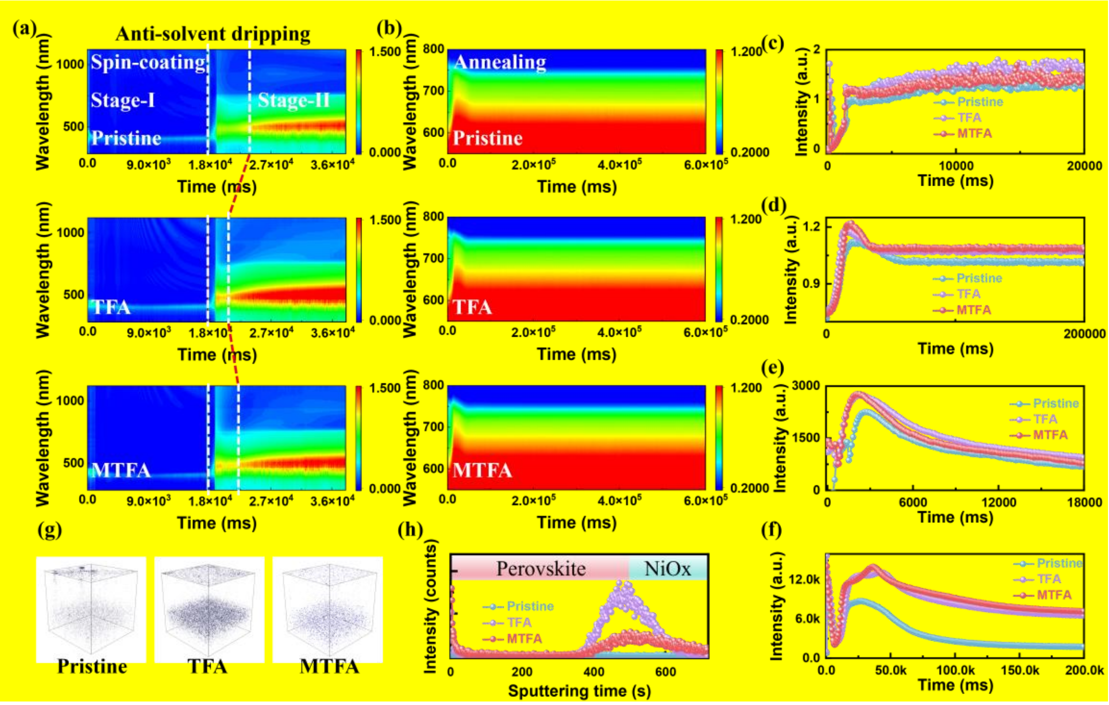

Figure 2. *In situ* UV-Vis images of the perovskite films during the **a)** spin-coating and **b)** annealing process. Evolution of absorption peak intensity at 480 nm for perovskite films during **c)** spin-coating and **d)** annealing process. Evolution of PL peak intensity at 760 nm for perovskite films during **e)** spin-coating and **f)** annealing process. **g)** The 3D spatial distribution of CF_3COO^- for the pristine/TFA/MTFA films. **h)** ToF-SIMS depth-profile analysis of CF_3COO^- in the pristine/TFA/MTFA films.

Question 9: *In general, Captions should be significantly more descriptive. For instance, Figure 5h should be labelled following ISOS-T conventions (including temperature, encapsulation, storage, and measurement conditions). T90 reference lines should also be included.*

Response: We sincerely thank the reviewer for this essential critique regarding device aging standards. We fully agree that comprehensive and standardized Figure captions are crucial for reproducibility and clarity, especially for stability tests. We apologize for this oversight in our initial submission manuscript and now thoroughly revised the captions for all stability tests as follows:

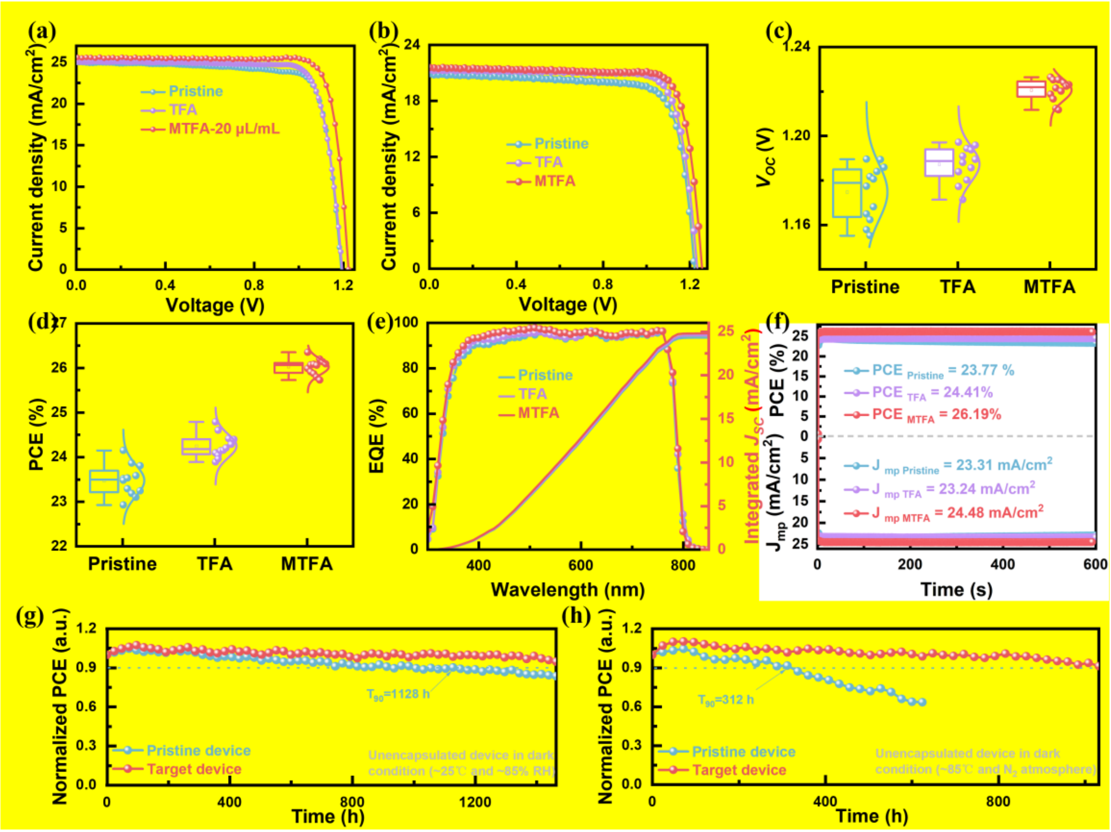

Figure 5 (a) *J*-*V* curves of champion PSCs with active area of 0.09 cm^2. **(b)** *J*-*V* curves of champion WB PSCs with active area of 0.09 cm^2. **(c)** PCE and **(d)** V_{oc} statistic distributions of 12 independent pristine device and corrosion inhibitor devices. **(e)** EQE spectrum and **(f)** Steady-state output performance of the pristine device and corrosion inhibitor device. **(g)** moisture stability and **(h)** thermal stability test result of the pristine device and MTFA device.

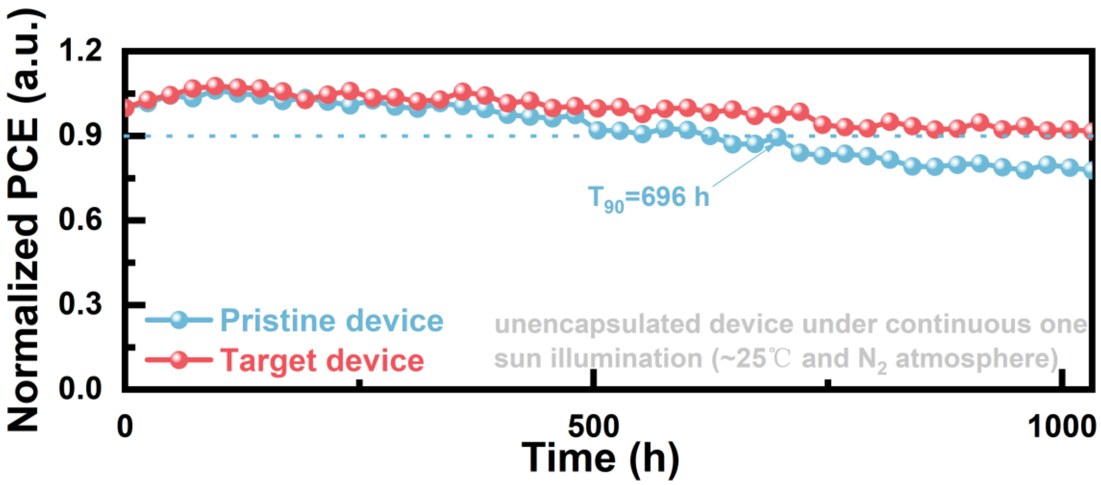

Figure S38. MPP tracking test of unencapsulated PSCs under one sun irradiation in a

~25 ± 2 °C N_2 atmosphere.

Reviewer #4: *The manuscript presents a well-motivated and timely study on stabilizing FA-based perovskite precursor solutions by introducing methyl trifluoroacetate (MTFA). The authors provide a detailed mechanistic analysis of deprotonation pathways and convincingly show that MTFA undergoes gradual hydrolysis to deliver controlled TFA generation while suppressing competitive proton transfer and byproduct formation. This precursor-solution stabilization concept is meaningful and has clear relevance to reproducible, high-quality perovskite film fabrication. Overall, this manuscript explores an important challenge in perovskite solar cell fabrication and proposes a promising solution-stabilization strategy. With clearer reporting of precursor aging conditions and the inclusion of time-dependent film and device data that directly reflect the anti-aging effect, the study will be significantly strengthened and provide compelling evidence for the advantages of MTFA in practical processing. Therefore, several clarifications and additions are needed before publication.*

Response: We are deeply and sincerely grateful for your positive and encouraging evaluation of our work. We particularly value the acknowledgment that the precursor-stabilization strategy holds significant promise for enabling reproducible and scalable fabrication. We fully concur with your insight that to elevate this study from a promising approach to a compelling and practically impactful demonstration, it is imperative to deliver more transparent reporting of the aging dynamics and to present time-resolved evidence that firmly links solution stability to the evolution of device performance. In direct response to this pivotal recommendation, we have undertaken a comprehensive revision, expanding the experimental framework, enriching the analytical depth in our revised manuscript and looking forward to your approval.

Question 1: *Although the manuscript emphasizes the critical importance of precursor aging, the actual aging conditions of the solutions used to fabricate the perovskite films and devices are not explicitly stated. It is unclear how long the pristine, TFA-treated, and MTFA-treated solutions were stored prior to use, or whether they were compared under equivalent storage durations. Since the central significance of MTFA lies in its ability to maintain stability during storage, presenting film and device characteristics as a function of controlled aging time for all three precursor solutions would strengthen*

the manuscript considerably. Such data would clearly demonstrate the practical advantage of MTFA and would allow the reader to assess how differences in deprotonation and byproduct formation directly influence film morphology, crystallization dynamics, defect states, and photovoltaic performance.

Response: Thank you for your expert and invaluable suggestions toward further improving our manuscript. It is absolutely right that without a clear and time-dependent comparison, the asserted advantage of MTFA-assisted deprotonation suppression strategy remains a conceptual rather than empirically substantiated. In direct and comprehensive response to this essential feedback, we have conducted a series of rigorous experiments to establish a precisely controlled aging study, systematically tracking the evolution of solution chemistry, film quality, and device performance over time. These comprehensive revisions have now been seamlessly integrated in our revised manuscript of section "**Evolution of Perovskite Precursor Solutions Deprotonation**".

Explicit Aging Protocol: In our revised manuscript, we have clearly defined that all PPSs were stored under identical ambient conditions (~25 °C, ~35% RH), and the entire aging period was defined as 30 days with data extraction interval of 10 days.

Solution Stability as a Function of Time: Deprotonation extent of PPSs doped with various corrosion inhibitors were firstly clarified in our revised manuscript (**Fig. 1a-c**, **Fig. S1-7** and **Table S1-2**). All of them declared that both the pristine and TFA PPSs have acquired climbing DoD values across the entirely aging period, while all MTFA-assisted PPSs with various doping concentration (10/20/30 μL/mL) stated a marked DoD values decline during the aging period from 0th to 20th days followed by an upturn, obtaining the minimum DoD values on the 20th days before rebounded due to the controllable hydrolysis reaction as evidence by **Fig. S10-11**.

Film Characteristics as a Function of Time: XRD characterization were conducted on perovskite films fabricated from each PPSs at each aging time point, corresponding patterns were illustrated in **Fig. S12-13**. Which revealed that films prepared from MTFA-doped solutions maintain superior crystallinity over 20 days, while those from pristine and TFA-doped solutions degrade progressively.

Device Performance as a Function of Time: Fig. S14-16 and **Table S4-5** presented evolution of PCE for devices fabricated with different PPSs as a function of the aging period. Which unequivocally declared the trend of device performance degradation were consistent well with the evolution process of PPSs deprotonation extent and crystalline of relevant perovskite films. While the champion PCE for both the fresh and aged PSCs were achieved by the 20 μL/mL MTFA-doped device with values 26.24% and 26.35% respectively.

Question 2: *The paper also offers extensive analysis of solution-phase chemistry, including NMR quantification, HI detection, and simulation results, but the connection between these solution-level changes and the subsequent film-formation processes could be made more explicit. Clarifying how the extent of deprotonation or hydrolysis in the solution translates into the nucleation behavior, growth kinetics, and defect landscape of the resulting films would provide deeper mechanistic insight and better align with the overall message of the work.*

Response: Thank you sincerely for your approval of our extensive analysis and thoughtful suggestion to strengthen the connection between the solution-level changes and the subsequent film-formation processes. In response, we have reconstructed the theoretical analysis in our manuscript, typical update contents are as follows:

Part 1: An extraordinary and striking observation that demanded emphatic attention in aforementioned analysis is the apparent conflict between the superior deprotonation suppression capability of the 30 μL/mL MTFA-doped PPS and the exceptional crystallinity as well as higher photovoltaic performance exhibited by the 20 μL/mL MTFA-doped precursor or corresponding device. Which is probably derived from the more standardized crystallization dynamics belonged to the moderate MTFA-doping concentration. Consequently, the coordination affinity between two corrosion inhibitors and perovskite films was firstly investigated comprehensive.

Part 2: Subsequently, all samples were transitioned into a plateau stage as the annealing duration extended, while the champion absorption intensity in this region is belonged to the MTFA sample, thereby underscoring its superior crystalline characteristic induced by the synergistic action of suitable crystallization dynamic regulation and

balanced stoichiometric ratio within PPS derived from suppressing cation deprotonation.

Part 3: Additionally, it is widely recognized that white flakes (highlighted by yellow ovals) adorning the surface of the pristine and TFA samples are classified as unreacted PbI_2 species, originating from the disruptive stoichiometric ratio within the PPS induced by the uncrossed cation deprotonation[66,67].

Question 3: *In addition, the interpretation of the increased built-in potential would benefit from some form of direct energetic evidence, such as UPS or KPFM, to support the discussion of interfacial band bending.*

Response: We sincerely thank you for your constructive suggestion to strengthen the evidence for the enhanced built-in potential and fully agree that direct measurement of the interfacial energetics would provide more robust support for our discussion on band bending. From this, we have performed Kelvin Probe Force Microscopy (KPFM) measurements to directly probe the surface potential and work function changes induced by the TFA and MTFA additives as follow:

This elevated V_{bi} in corrosion inhibitor devices is primarily attributed to the superior surface potential of the relevant perovskite films as declared by Kelvin probe force microscope (KPFM) images in **Fig. S36**. While the surface potential of TFA (287.34 mV) and MTFA (346.17 mV) films were significantly outclass that of the pristine film (2.93 mV). Indicating a lower effective work function at the perovskite surface as well as a more pronounced upward band bending at the perovskite/electron transport layer interface. Thereby, providing a greater driving force for charge carrier separation and extraction, ultimately leading to the observed increase in V_{bi}.

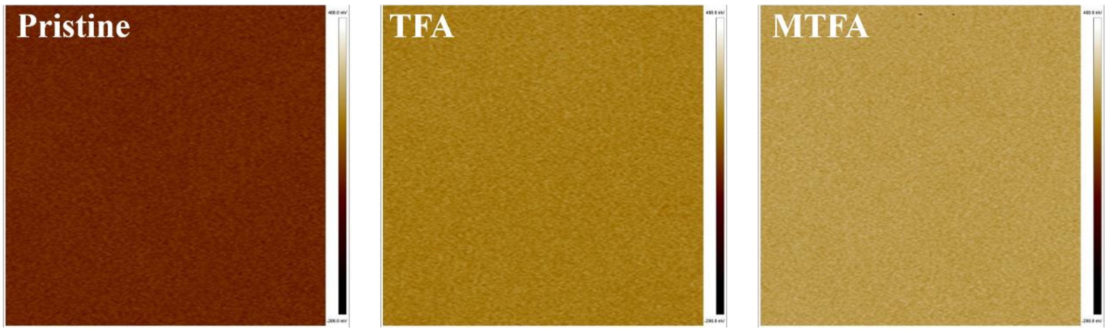

Figure S36. KPFM images of perovskite films with different corrosion inhibitors.

__Question 4:__ The X-axis label in Figure S17 should be changed.

Response: We sincerely thank you for identifying this critical error and we have already corrected the X-axis label in both **Fig. 3h** and **Fig. S17** in our revised manuscript as follows:

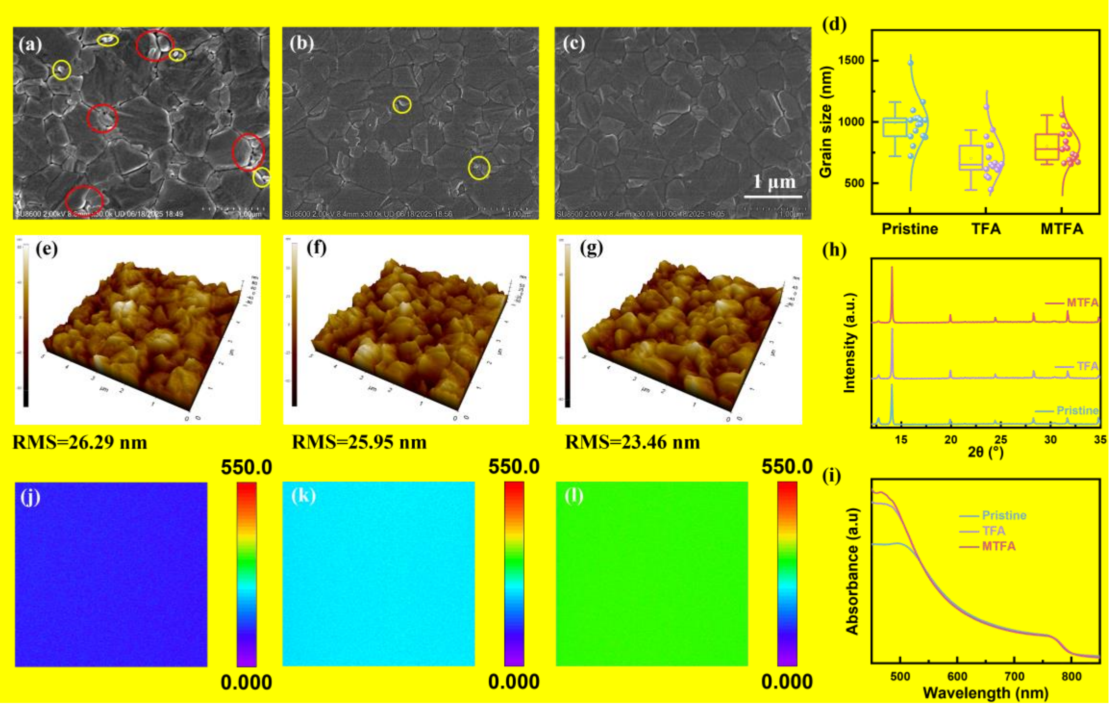

Figure 3. Top view SEM images of **(a)** the pristine sample, **(b)** the TFA sample and **(c)** the MTFA sample. **(d)** Statistical grain sizes of final perovskite films. AFM images and corresponding RMS values of **(e)** the pristine sample, **(f)** the TFA sample and **(g)** the MTFA sample. **(h)** XRD patterns and **(i)** UV–vis absorption spectrum of final perovskite films. PL lifetime in PL mapping images of **(j)** the pristine sample, **(k)** the TFA sample and **(l)** the MTFA sample.

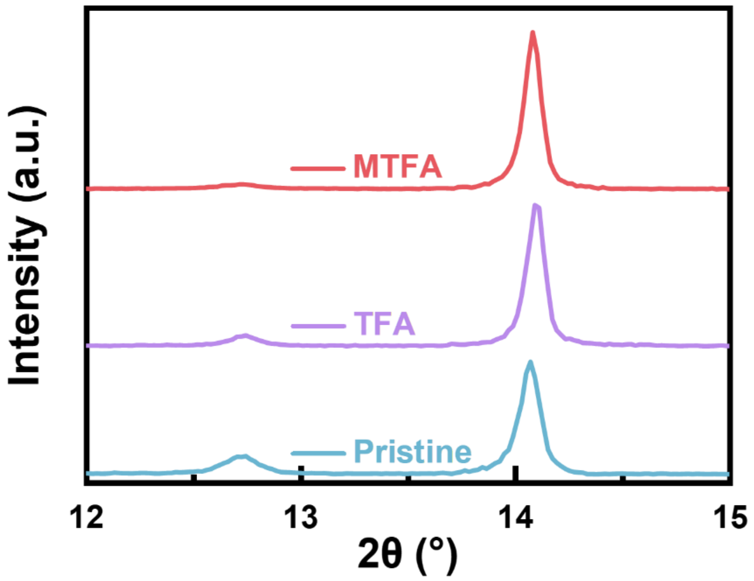

Figure S28. Partially enlarged (100) diffraction peaks for perovskite films with various corrosion inhibitors.

Reply to the Reviewer's Comments

Dear Editor,

Thank you very for your Letter dated 3rd Mar. 2026 to inform us to revise our manuscript entitled "Deprotonation Suppressing via Competitive Proton Transfer Control for Efficient Perovskite Solar Cells". We thank you and the reviewers for constructive comments and suggestions on our manuscript as well as on our work. According to the comments, we have done our best to make revisions on our manuscripts. All the modifications are highlighted by ***yellow background*** in the revised manuscript for clarity.

Our point-by-point responses to the reviewers' Comments/Questions are provided as followings.

We hope this improved version has met the requirements for publication in Nature Communications.

Many thanks again to you and the reviewers.

Correspondence should be addressed to:

Prof. Chunfu Zhang, email address: cfzhang@xidian.edu.cn.

Sincerely yours,

Chunfu Zhang

School of Microelectronics, Xidian University

Xi'an 710071, P. R. China.

Reviewer #1:

The authors have addressed some of the raised concerns in their revision. However, the science of the data is still concern to me. Hence, this manuscript is recommended for publication after minor revision, and the detailed comments are as follows:

Comment 1: The authors should add X-ray absorption spectroscopy (XAS) to substantiate the proposed coordination environment.

Response: Thank you for your kind suggestions of adding XAS for further improving our manuscript. We fully agree that XAS is a powerful and direct technique to probe the local coordination environment around Pb^{2+}, which would provide definitive evidence for the formation of C=O→Pb^{2+} bonding between TFA/MTFA and uncoordinated Pb^{2+}. Therefore, we have added the XAS measurements of perovskite films with various corrosion inhibitors in our revision manuscript as follows:

Additionally, X-ray absorption spectroscopy (XAS) was further conducted to inquire the atomic environment around Pb^{2+} and to confirm the molecular interaction mechanisms elucidated by FTIR, XPS, and DFT calculations. Therein, X-ray absorption near-edge structure (XANES) spectra of perovskite films with various corrosion inhibitors were illustrated in **Fig. S24a-b**, revealing a clear blue shifted Pb L_3-edge absorption edge for both the TFA and MTFA samples compared to the pristine one, with the most pronounced blueshift observed for the TFA sample. This blue shift indicated a reduction in electron density around Pb^{2+}, arising from coordination interactions between TFA/MTFA and Pb^{2+} sites and further amplified by the higher electronegativity of oxygen. Notably, the local bonding environment was further acquired using Fourier transform extended X-ray-absorption fine structure (FT-EXAFS) spectra in **Fig. S24c-d**. Two dominant signals appeared at ≈1.91 Å and ≈2.97 Å in R-space for all samples are related to the Pb-O band and Pb-I band respectively. All corrosion inhibitors samples assumed a markedly attenuated Pb-I peak intensity accompanied by an enhanced Pb-O signal, especially for the TFA sample.

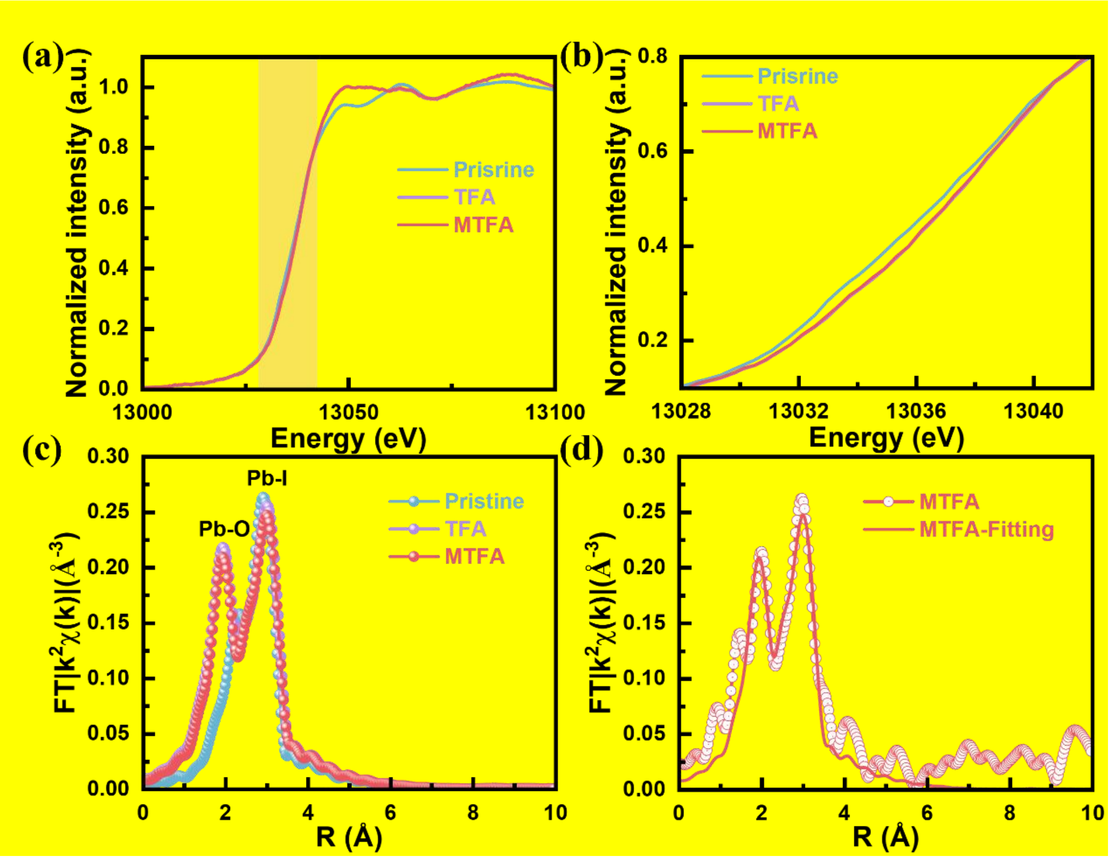

Figure S24. XAS measurements of perovskite films with various corrosion inhibitors **(a)** Normalized Pb L_3-edged absorption coefficient, **(b)** The partial enlarged Normalized Pb L_3-edged absorption coefficient indexed to the energy range from 13028 eV to 13042 eV, **(c)** FT-EXAFS spectra in R-space for pristine, TFA and MTFA perovskite films and **(d)** EXAFS raw data and fitting spectrum of MTFA perovskite film.

Reviewer #2:

Comment 1: The authors have responded comprehensively and convincingly to all reviewer comments. The revised manuscript shows a substantial improvement in scientific rigor, clarity, and presentation compared to the original submission. The work presents a meaningful conceptual advance in stabilizing perovskite precursor solutions and offers clear relevance for scalable and reproducible perovskite solar cell fabrication. I believe the manuscript is now suitable for publication in Nature Communications.

Response: Thank you very much for your positive decision on our manuscript. We are delighted to learn that our revisions have fully addressed your concerns and that the

manuscript is now deemed suitable for publication in *Nature Communications*.

Reviewer #3: The authors have improved the manuscript substantially by performing additional experiments and incorporating new discussions in both the main text and Supporting Information.

Comment 1: As a note regarding the TRPL analysis:

Under identical excitation and measurement conditions, TRPL decays can indeed be compared on a relative basis. However, fitting the decay curves with a bi-exponential function does not, by itself, provide direct physical insight into the underlying photophysical processes.

In particular, assigning the fast component (τ_1) to non-radiative recombination and the slow component (τ_2) to bulk recombination is not physically justified without additional supporting evidence. Although many published papers follow a similar bi-exponential fitting approach and interpret τ_1 and τ_2 in this manner, the prevalence of this practice does not necessarily validate the physical interpretation. Therefore, the following discussion is not rigorously supported:

"Herein, A_1 and A_2 represent the respective amplitudes, while τ_1 and τ_2 denote the PL decay times. Since the rapidly decay component (τ_1) is assigned to the non-radiative recombination resulting from charge-trapping defects as well as the quenching of charge carriers at the interfaces. Meanwhile, the slower decay component (τ_2) is associated with the recombination processes occurring within the film bulk…"

Such mechanistic attribution goes beyond what can be concluded from biexponential fitting alone.

Instead, I recommend restricting the interpretation to a comparative analysis based on the average lifetime under identical measurement conditions, without assigning specific recombination channels to individual decay constants. This would make the TRPL discussion physically sound while still supporting the comparative conclusions of the study.

Response: We sincerely appreciate your thoughtful suggestion regarding the oversimplified analysis of the TRPL decay curves. In response, we have revised the relevant discussion in the manuscript as follows:

TRPL decay profiles illustrated in **Fig. S32c** was fitted by a bi-exponential decay equation to elucidate the average photo-generation carrier lifetime (τ_{avg}) within perovskite films:

$$\tau_{avg} = \frac{A_1\tau_1^2 + A_2\tau_2^2}{A_1\tau_1 + A_2\tau_2} \tag{7}$$

Herein, A_1 and A_2 represent the respective amplitudes, while τ_1 and τ_2 denote the PL decay times. As enumerated in **Table S8**, the average lifetime indexed to the TFA and MTFA samples was extremely extended to 259.88 ns and 301.90 ns respectively compared to 191.17 ns for the pristine sample. This indicates the capacity of the corrosion inhibitor samples in promoting charge extraction and transfer behavior, with the MTFA sample achieving the most pronounced improvement. Collectively, these results compellingly imply the extraordinary efficacy of MTFA in suppressing cation deprotonation, passivating halogen vacancy as well as orchestrating crystallization dynamic, which are indispensable for the fabrication of high-quality perovskite film with conspicuous crystalline characteristic and surface texture characteristic, accompanied by satisfactory carrier dynamic.

Reviewer #4:

Comment 1: The authors have carefully and comprehensively addressed all of the reviewer's concerns in the revised manuscript. In particular, the explicit clarification of precursor aging conditions, the inclusion of time-dependent film and device data, and the additional energetic characterization significantly strengthen the scientific rigor and transparency of the study. The revised version now presents a clear and convincing link between solution stability, crystallization behavior, and device performance evolution. In my opinion, the manuscript has been substantially improved and now provides compelling evidence supporting the proposed MTFA-assisted precursor stabilization strategy. I therefore recommend publication of the revised manuscript in its present form.

Response: We sincerely thank you for your favorable decision regarding our manuscript. We greatly appreciate both your approval of the revised version and your constructive feedback for strengthened the rigor and clarity of our work.
